# Strain-level metagenomic profiling using pangenome graphs with PanTax

**DOI:** 10.1101/gr.280858.125

**Published:** 2026-02

**Authors:** Wenhai Zhang, Yuansheng Liu, Guangyi Li, Jialu Xu, Enlian Chen, Alexander Schönhuth, Xiao Luo

**Affiliations:** 1 Hunan Research Center of the Basic Discipline for Cell Signaling, Hunan University, Changsha, Hunan 410082, China;; 2 College of Biology, Hunan University, Changsha, Hunan 410082, China;; 3 College of Computer Science and Electronic Engineering, Hunan University, Changsha, Hunan 410082, China;; 4 Faculty of Technology, Bielefeld University, Bielefeld 33615, Germany

## Abstract

Microbes are omnipresent, thriving in a range of habitats, from oceans to soils, and even within our gastrointestinal tracts. They play a vital role in maintaining ecological equilibrium and promoting the health of their hosts. Consequently, understanding the diversity in terms of strains in microbial communities is crucial, as variations between strains can lead to different phenotypic expressions or diverse biological functions. However, current methods for taxonomic classification from metagenomic sequencing data have several limitations, including their reliance solely on species resolution, support for either short or long reads, or their confinement to a given single species. Most notably, most existing strain-level taxonomic classifiers rely on the sequence representation of multiple linear reference genomes, which fails to capture the sequence correlations among these genomes, potentially introducing ambiguity and biases in metagenomic profiling. Here, we present PanTax, a pangenome graph-based taxonomic profiler that overcomes the shortcomings of sequence-based approaches, because pangenome graphs possess the capability to depict the full range of genetic variability present across multiple evolutionarily or environmentally related genomes. PanTax provides a comprehensive solution to taxonomic classification for strain resolution, compatibility with both short and long reads, and compatibility with single or multiple species. Extensive benchmarking results demonstrate that PanTax drastically outperforms state-of-the-art approaches, primarily evidenced by its significantly higher F1 score at the strain level, while maintaining comparable or better performance in other aspects across various data sets.

Microorganisms are ubiquitous on Earth, inhabiting diverse environments such as oceans, soils, and gastrointestinal tracts, where they play indispensable roles in maintaining ecological balance and host health. Microbial communities are often composed of multiple types of microorganisms, with uneven species abundance and high diversity. Rapid mutation and horizontal gene transfer in microorganisms may result in different strains of the same species, which further increases the biological diversity and complexity of microbial communities. Diversity of microbial communities at the level of strains is a key factor in microbiome-related research, as it does not only reflect the evolution and adaptation of microorganisms, but also their interactions with the environment and the functions of their host. Previous studies have shown that different strains may exhibit different phenotypes or perform different biological functions in the environment (Marx 2016; Van Rossum et al. 2020). Moreover, recent works have revealed that strain-level genome variations in the gut microbiota are closely related to host health and disease (Zeevi et al. 2019; Chen et al. 2022; Zhernakova et al. 2024). This means that determining the composition of gut microbiota in terms of their strains can accurately predict intestinal diseases such as inflammatory bowel disease (Jiang et al. 2022) and is important for understanding the biological characteristics of microorganisms in general and their involvement in scenarios of clinical interest in particular (Bickhart et al. 2022; Fedarko et al. 2022; Zheng et al. 2022). Therefore, to reveal the structure and function of microbial communities, we need to estimate their composition and abundance at finest resolution possible, that is, not only at the level of species, but also at the level of strains.

For this study, we define a “strain” as a microbial genome with sufficient genomic variation to be distinguished from other genomes within the same species. This definition aligns with previous studies (Vicedomini et al. 2021; Liao et al. 2023b), although we acknowledge that the term “strain” may be interpreted differently in traditional taxonomy and other areas of microbial evolution.

Metagenomic sequencing preserves the majority of the genetic information and enables a strain-level analysis of microbial communities. This establishes significant advantages over amplicon sequencing and culture-based methods, because these two approaches, by design, inevitably miss large parts of the genetic material one seeks to analyze. High-throughput sequencing or short-read sequencing (SRS), such as Illumina sequencing, has been routinely used in reference-based taxonomic classification of metagenomic data, which explains the development of many related tools. The fundamental tasks are to classify or group sequencing reads into distinct genomic bins, each representing a different taxon (e.g., species or strain) within a metagenomic sample (taxonomic binning), and to identify the taxa present while estimating their relative abundances in the sample (taxonomic profiling) (Simon et al. 2019; Sun et al. 2021). Despite their differences, these tools are frequently used interchangeably in the metagenomic data analysis.

Various tools have been developed that operate at the level of species alone. However, strain-level analysis has still remained a tough challenge. All of the species-level tools receive the sequenced reads of a metagenome as input, and output the spectrum of species that make part of the metagenome, and also possibly their relative sequence abundances (the proportion of reads assigned to a given taxon relative to the total reads) or relative taxonomic abundances (the proportion of genomes of a given taxon to the total genomes detected) (Simon et al. 2019). Note that none of these tools aims to assemble the individual genomes prior to raising the relevant estimates. Rather, they put the reads of a metagenome into context with existing reference sequences and derive the corresponding conclusions from the resulting read-to-reference sequence alignments.

Existing tools can be categorized into three types. The first are marker-based methods, such as MetaPhlAn4 (Blanco-Míguez et al. 2023) and mOTUs2 (Milanese et al. 2019). These methods utilize a subset of marker genes, typically gene families that are indicative of species. For example, whereas the 16S rRNA sequence is commonly used in some microbiome analysis methods due to its high conservation (Edgar 2018), tools like MetaPhlAn4 and mOTUs rely on a broader set of specific taxon-specific markers. Although these methods are computationally efficient, the classification can be biased if the marker genes in the microbial sequences of interest do not follow a uniform distribution (D’Amore et al. 2016). The second are DNA-to-protein methods, where DIAMOND (Buchfink et al. 2015), Kaiju (Menzel et al. 2016), and MMseqs2 (Steinegger and Söding 2017) are prominent examples. DNA-to-protein methods focus on the protein-coding sequences of the genomes, while discarding all noncoding sequences from further consideration. This prevents the monitoring of differences that occur in the noncoding portions of the bacterial genomes. The third are DNA-to-DNA methods, such as Kraken2 (Wood et al. 2019), KrakenUniq (Breitwieser et al. 2018), Bracken (Lu et al. 2017), CLARK (Ounit et al. 2015), Centrifuge (Kim et al. 2016), Centrifuger (Song and Langmead 2024), CAMMiQ (Zhu et al. 2022), KMCP (Shen et al. 2023), Ganon (Piro and Reinert 2025), and sylph (Shaw and Yu 2024) characterized by considering whole-genome information for classification.

However, most of these methods were originally designed for taxonomic classification at the species level and do not perform as effectively at the strain level. Strain-level microbiome composition analysis tools that have been suggested recently, such as StrainScan (Liao et al. 2023b), StrainGE (van Dijk et al. 2022), or StrainEst (Albanese and Donati 2017), can only handle one species/genus specified by the user as input. They can only handle one species/genus that preassigned by users.

These tools are specifically designed to address the unique characteristics of metagenomes, distinguishing them from tools used for classifying isolated genomes, such as those derived from individual organisms grown on Petri dishes or from environments that do not contain whole microbial communities. Approaches that address the classification of isolates, but not metagenomes, are ProPhyle (Břinda et al. 2017) and Phylign (Břinda et al. 2025), for example.

Short-read sequencing, characterized by reads of a few hundred base pairs (bp) in length, has been the preferred method for metagenomics due to its ubiquitous availability and its low cost. In the meantime, long-read sequencing (LRS) technologies such as Pacific Biosciences (PacBio) and Oxford Nanopore Technologies (ONT), have become more affordable. Long-read sequencing has three advantages over short-read sequencing when dealing with metagenomic assignment and estimating the bacterial composition. First, long reads, of length tens of kbp to Mbp, retain much longer-range genomic information. Therefore, they can more easily distinguish intra-genomic repeats or highly similar strains, which supports the disambiguation of reads when determining the strain they stem from. Second, as per the basic properties of single-molecule sequencing technologies, long-read sequencing (PacBio and ONT) avoids the generation of PCR duplicates common to short-read sequencing, which avoids the corresponding biases during taxonomic profiling. Third, portable long-read sequencing sequencers, such as ONT Flongle and MinION, enable cost-effective, in-field and real-time metagenomic sequencing. This can be used in scenarios where speed of identification of microbial/viral components matters, such as, for example, during pandemics (Quick et al. 2016; Brinkmann et al. 2021). Urgency may also be a factor in scenarios where samples are difficult to culture or preserve (Runtuwene et al. 2019; Wang et al. 2021), or when analyzing samples raised in hospitals (Chng et al. 2020).

Still, the majority of taxonomic classifiers are based on short-read sequencing data. Because the corresponding built-in algorithms are not suitable for querying sequences that are long and/or affected by high error rates, their underlying technology cannot be straightforwardly adapted to processing long reads. Therefore, several short-read-based tools, such as Kraken2, KrakenUniq, KMCP, and Ganon, can successfully process long read. However, they may encounter challenges in yielding satisfactory results.

The difficulties involved in the adaptation of tools from short-read sequencing to long-read sequencing, as just outlined, explains why developing novel approaches for long-read-based taxonomic classification is imperative. So far, only few related tools have been designed, such as Melon (marker-based) (Chen et al. 2024), MEGAN-LR (DNA-to-protein) (Huson et al. 2018), and MetaMaps (DNA-to-DNA) (Dilthey et al. 2019). Just as for the tools already listed above, the characteristic drawbacks of the above-mentioned three categories apply also here. When raising a summarizing overall account of the characteristics of tools (Supplemental Table S1), Centrifuge and Centrifuger emerge as the methods that can handle both short-read sequencing and long-read sequencing data and perform strain-level taxonomic profiling for multiple species at a time, so proves to be the tools already available to address all currently driving issues in a universal manner. In addition, from a methodological perspective, existing strain-level profiling methods rely solely on plain linear reference genomes. This common limitation prevents them from effectively capturing and representing genetic variations among strains within a species, resulting in ambiguous read assignments and inaccurate abundance estimates.

To overcome the shortcomings of existing taxonomic metagenome classifiers, we suggest Pangenome graph-based Taxonomic classifier (PanTax), as a new method to perform strain-level taxonomic profiling for both short-read and long-read metagenomic data.

From a methodological point of view, PanTax’s innovation is the systematic integration of pangenome graphs, instead of plain linear sequences, as an algorithmic foundation for taxonomic classification. The particular type of pangenome graphs that PanTax relies on are variation graphs, as originally described in Paten et al. (2017). In the meantime, variation graphs have proven effective in addressing a wide range of computational genomics challenges. Examples are primary read mapping and variant calling (Garrison et al. 2018; Martiniano et al. 2020; Sirén et al. 2021), haplotype modeling (Rosen et al. 2017), as well as the refined correction of errors in long reads (Luo et al. 2022). For the latter, greatest improvements were observed for long reads stemming from metagenomes. The favorable usage of variation graphs in the assembly of genomes from mixed samples (Baaijens et al. 2019, 2020) is an additional hint to the particular advantages of variation graphs in the analysis of metagenomes. The implementation of PanTax is fundamentally enabled by the topology of variation graphs. As a specific form of genome graph, variation graphs collapse homologous regions and represent them as alternative paths within the graph. When constructed from closely related genomes (e.g., within a species), such graph-based alignments, compared with alignments to linear reference genomes, substantially reduce ambiguous read assignments. Importantly, strain-specific nodes within the graph capture unique genomic sequences of individual strains. Unlike isolated *k*-mers assigned via lowest common ancestor (LCA) strategies, as in tools such as Kraken2, these nodes are embedded in a broader topological context through their connections to neighboring nodes. This structural context confers two major advantages: (1) It enables abundance estimation via linear optimization directly on alignment results and (2) it enhances the discrimination of authentic strain-specific signals, thereby enabling more accurate strain recall and abundance estimation. To the best of our knowledge, PanTax is the first approach that employs pangenome graphs as whole genome-wide reference systems for the taxonomic profiling of metagenomes.

To provide evidence of PanTax’s superiority, we have conducted various challenging experiments and compared PanTax with the state-of-the-art taxonomic classifiers on both simulated and real data sets that relate to questions of actual interest in research. The corresponding benchmarking experiments consistently demonstrate that PanTax achieves the top-tier performance rates in taxonomic profiling, across all popular sequencing platforms.

## Results

We have developed and implemented PanTax, a novel method for classifying the contents of metagenomes. PanTax is universal insofar as it (1) evaluates whole genomes, (2) supports either short-read sequencing or long-read sequencing data, (3) can process multiple species simultaneously, (4) refers to a custom database, which avoids the biases of external databases, and (5) provides estimates on the taxonomic abundances of the involved strains. As was pointed out on plenty of occasions, all of these points are of critical relevance in the analysis of metagenomes.

The foundation for PanTax’s superiority is to employ pangenome graphs, instead of linear genome sequences as a basis for the required reference systems. Unlike linear genome sequences, pangenome graphs, and here variation graphs in particular, have the capability to capture the full range of genetic variability characteristic of an evolutionarily or environmentally related collection of genomes (Paten et al. 2017). PanTax exploits this quality of variation graphs for capturing the genetic variation that distinguishes the different strains of a species in particular. In addition to this principled quality, pangenome graphs simply offer a considerably more compact way of representing multiple genomes in an encompassing context (Garrison et al. 2018; Sirén et al. 2021). The practical advantages of pangenome graphs are the preservation of the strain-level diversity of metagenomes and the lack of ambiguity inducing redundancies by which read binning-based approaches are affected.

In the following, we first present a comprehensive summary of the workflow of PanTax. Subsequently, we assess its performance at the level of strains in comparison with all prominent state-of-the-art approaches, by experiments using both simulated and real data.

### Approach

**Figure 1.**
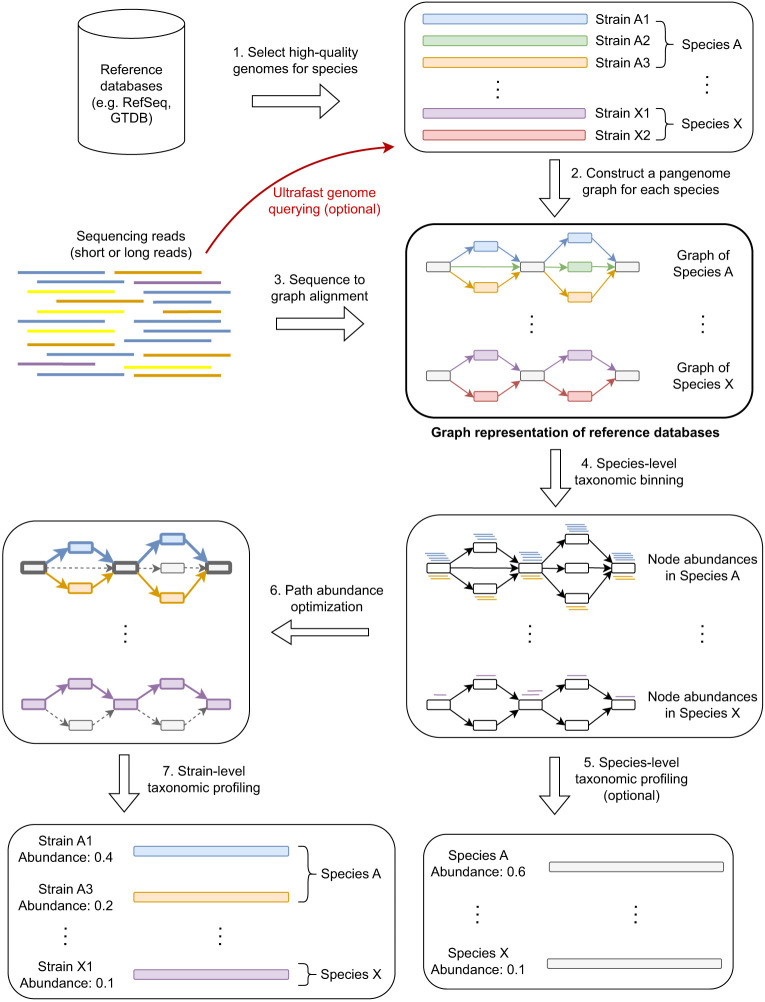
Workflow of PanTax. Different colors indicate different strains. The gray and colored rectangles in the pangenome graphs indicate the shared and unique genomic segments among strains, respectively. Arrows with the same color spell the path of a strain. The bold paths and dashed paths in the pangenome graphs indicate the existent and nonexistent strains in a sample, respectively. For simplicity, only two species (A and X) are shown in the figure. Note that Step 5 is optional unless one requires to output the species-level taxonomic profile. The “ultrafast genome querying” step (see Methods for details) marked with the red arrow and red text indicate the optional way to proceed when running the accelerated version of PanTax (= PanTax (fast)).

Figure 1 illustrates the workflow of PanTax. Here, we provide an overview of the workflow. For full details, see the Methods section.

PanTax consists of three stages. The first stage is to construct a pangenome graph-based reference database (Steps 1 and 2 in Fig. 1), where each species that makes part of the metagenome corresponds to a species-specific pangenome graph. The second stage (Steps 3–5 in Fig. 1) yields species-level taxonomic classification results, by evaluating the amount of reads that get aligned to each of the species graphs. The third stage eventually determines the strains and the corresponding relative abundances in a species (Steps 6 and 7 in Fig. 1), based on the fact that the individual strains of a species correspond to the individual paths in a species pangenome graph.

From a technical point of view, the first stage reflects to construct a pangenome graph from a collection of high-quality strain-level genomes as stored in reference genome databases such as NCBI’s RefSeq (O’Leary et al. 2016) and Genome Taxonomy Database (GTDB) (Parks et al. 2022), or, if available, in more specific, customized databases. The choice of genome collection can be determined by the user. The more refined collections there are available, the more accurate the results. Note that the flexibility in terms of database usage accounts for the fact that microbe genome databases are filling up rapidly; so, PanTax does not depend on a static snapshot of available microbial sequences, but is explicitly tailored to keep up with the rapid progress in this area of research.

Subsequently, for each read, one needs to determine the species-level graph that gives rise to the optimal read-to-graph alignment of that read. To considerably enhance this step, all species-level pangenome graphs are virtually merged into a large “pangenome super-graph” that captures all species that one has recorded in the first stage. The advantage of merging individual species graphs into one large graph is the fact that one has to align each read with this one large graph, instead of having to align it with each species graph. Apart from drastically decreasing the number of alignment operations, this also facilitates the evaluation of the possibly several alignments of an individual read with different species in a statistically unifying context.

PanTax supports alignment operations for both long and short reads. Based on the resulting alignments, the reads are classified at the level of species. Subsequently, the relative abundance of each of the species is estimated by evaluating the resulting (appropriately normalized) read coverages. Eventually, strain-level profiling can be performed by solving the path abundance optimization problem in each species-specific graph using linear programming (see Methods for definitions). Upon having estimated the abundance of each strain, potentially false positive strains are discarded based on the coverage information of nodes in graphs.

### Tasks

In this study, we focus on two computational tasks, namely, strain-level taxonomic profiling for multiple species and for single species. Strain-level taxonomic profiling for multiple species provides a detailed view of strain diversity and abundance across different species, enhancing our understanding of microbial dynamics. Additionally, strain-level profiling for a single species allows for in-depth analysis of strain variation within that species. To comprehensively benchmark performance, we evaluated PanTax alongside other state-of-the-art methods using both short-read (Illumina) and long-read (PacBio HiFi/CLR, ONT R10.4/9.4.1) data sets. We also evaluated the robustness of PanTax. See below for the details.

### Data sets

Here, we briefly describe the data sets used for the two computational tasks: strain-level taxonomic profiling for multiple species and for a single species. Complete details, including simulation procedures and real data access, are provided in the Supplemental Methods S1. The data sets used for evaluating PanTax’s robustness are described in the corresponding evaluation sections.

#### Data sets for multispecies strain-level taxonomic profiling evaluation tasks

##### Simulated data sets (sim-low, sim-high)

We made use of CAMISIM (Fritz et al. 2019) to generate 10 metagenomic data sets (2 × 5) with different complexities and different sequencing read types (i.e., Illumina, PacBio HiFi/CLR and ONT R10.4/R9.4.1 reads). The low complexity data set (named “sim-low”) comprises 60 strains from 30 species (two strains per species) with relative abundances ranging from 0.3% to 6.43%, corresponding to sequencing coverage of 0.96× to 20.3× per strain. The high complexity (named “sim-high”) data set comprises 1000 strains from 373 species (average two to three strains per species) with relative abundances from 0.04% to 0.30% and sequencing coverage of 1.0× to 8.8× per strain. All strains in the data sets follow a log-normal distribution. To mimic real-world scenarios with novel strains, some strains in our simulated data sets are absent from the pangenome reference databases (RefSeq:13404). For all data sets, the genomes used were retrieved from RefSeq (Supplemental Data).

##### Simulated data sets with introduced mutations (sim-low-mut1, sim-high-mut1, sim-low-mut2, sim-high-mut2)

To reflect natural strain variation, we generated mutant data sets by introducing random mutations (0.1% and 1%) into the previously simulated genomes. Specifically, sim-low-mut1 and sim-low-mut2 were derived from sim-low, whereas sim-high-mut1 and sim-high-mut2 were derived from sim-high, resulting in a total of 20 data sets across different sequencing technologies.

##### Simulated data sets for GTDB (sim-high-gtdb)

The high-complexity data set sim-high-gtdb comprises 1000 strains from the GTDB:206273 database, with sequencing coverage ranging from 1.1× to 9.0× . Illumina, PacBio HiFi/CLR, and ONT R10.4/R9.4.1 reads were simulated using CAMISIM.

##### Real data sets (Zymo1 and Zymo2, PD human gut, omnivorous human gut, healthy human gut)

*Zymo1 and Zymo2*. The ZymoBIOMICS Microbial Community Standard data sets D6300 and D6310, here referred to as Zymo1 and Zymo2, have defined bacterial compositions (Nicholls et al. 2019). Zymo1 contains evenly distributed bacteria (12% each) and two yeasts (2% each), whereas Zymo2 contains the same species with a logarithmic abundance distribution from 0.000089% to 89.1%. Sequencing reads for Zymo1 include Illumina, ONT R10, and ONT R9.4.1, whereas Zymo2 includes Illumina and ONT R9.4.1. Bacterial relative abundances were calculated based on genome copy number and used as ground truth.

*PD human gut (Illumina).* The Illumina sequencing data for individuals with Parkinson’s disease (PD) were obtained from the study by Wallen et al. (2022).

*Omnivorous human gut (PacBio HiFi)*. The PacBio HiFi metagenomic sequencing data from the fecal sample of a healthy Korean omnivorous individual were sourced from the study by Kim et al. (2022).

*Healthy human gut (ONT)*. The ONT sequencing data from the fecal sample of a healthy individual, free of any observable disease or infection, were obtained from the study by Chen et al. (2022).

##### Spiked-in data sets

To enable performance evaluation under realistic conditions with known ground truth, we generated spiked-in metagenomic data sets. Eight *Vibrio cholerae* strains were simulated using CAMISIM for five sequencing technologies, including Illumina, PacBio HiFi, PacBio CLR, and ONT R10.4 and R9.4.1. The average nucleotide identity (ANI) of strains ranged from 95.98% to 99.48%, and their sequencing coverage ranged from 2.4× to 6.0× . The simulated reads were spiked into real metagenomic data sets obtained from previous studies for each corresponding sequencing technology.

#### Data sets for single species strain-level taxonomic profiling evaluation tasks

##### Simulated data sets: Staphylococcus epidermidis strain mixtures (three strains, five strains, and 10 strains)

Three Illumina metagenomic data sets were simulated using CAMISIM with three, five, and 10 *S. epidermidis* strains. The ANI ranged from 99.20% to 99.33%, 98.62% to 99.33%, and 96.96% to 99.47% for the three-, five-, and 10-strain data sets, respectively, with sequencing coverages between 1× and 10× .

##### Real data sets: two cultured S. epidermidis strain mixtures

In the study by Emiola et al. (2020), the authors cultured two *S. epidermidis* strains (species taxid: 1282), NIHLM001 (RefSeq assembly accession: GCF_000276145.1) and NIHLM023 (RefSeq assembly accession: GCF_000276305.1), both isolated from human skin and sharing a 97% ANI. NIHLM001 displayed high resistance to the bacteriostatic antibiotic erythromycin, whereas NIHLM023 was susceptible. The two strains were mixed in equal proportions (1:1) and cultured under two conditions: one with erythromycin treatment (Ery) and the other without antibiotics (no_ATB). Samples were collected at three time points for metagenomic sequencing. For our analysis, we downloaded the Illumina data from the third biological replicate of each condition, resulting in a total of six sequencing data sets.

### Remarks on evaluating alternative methods

To ensure a fair and comprehensive evaluation, we benchmarked PanTax against a selection of representative metagenomic profilers (Supplemental Table S1) covering both short- and long-read sequencing data. The tools were chosen based on criteria including open-source availability, active maintenance, ability to build custom reference databases, and support for strain-level profiling. PanTax was evaluated in both the default and fast modes (see Step 1 in the Methods). The fast mode employs efficient filtering and on-demand pangenome construction to achieve a balance between accuracy and computational efficiency. To avoid systematic biases introduced by default reference databases (Simon et al. 2019), we used customized and unified reference databases across all tools. For strain-level profiling in multispecies experiments, we adopted either RefSeq:13404 or GTDB:206273 as the reference (see Step 1 in the Methods). Full details are provided in Supplemental Note S1 and all commands used for tool evaluations are provided in Supplemental Methods S17.

### Performance evaluation

To evaluate taxonomic classifiers, we rely on metrics like precision, recall, and F1 score, which assess the performance in terms of the identification of taxa (i.e., strains in our case). It is important to realize that these metrics are dominated by performance rates on high-abundance taxa. For the sake of an evaluation that does not neglect performance rates on low-abundance taxa, we also utilize precision-recall curves and the area under the curve (AUPR) (Supplemental Methods S15), which capture the desired effects. In addition, accurate estimation of the abundances of the taxa in metagenomes (i.e., profiling) is essential. We use L1 distance, L2 distance and Bray–Curtis (BC) distance to quantify the similarity of the estimated with the true abundance profiles. However, just as before when evaluating performance in binning, these popular distance metrics do not put performance on high-abundance into correct relative context with performance on low-abundance taxa, so tend to neglect the performance on low-abundance taxa. To appropriately account for performance on low-abundance taxa, we also introduce the complementary metrics absolute frequency error (AFE) and relative frequency error (RFE) so as to capture abundance estimation performance truly comprehensively. See Supplemental Methods S14 for full details.

### Strain-level taxonomic profiling for multiple species

#### Simulated data sets

**Figure 2.**
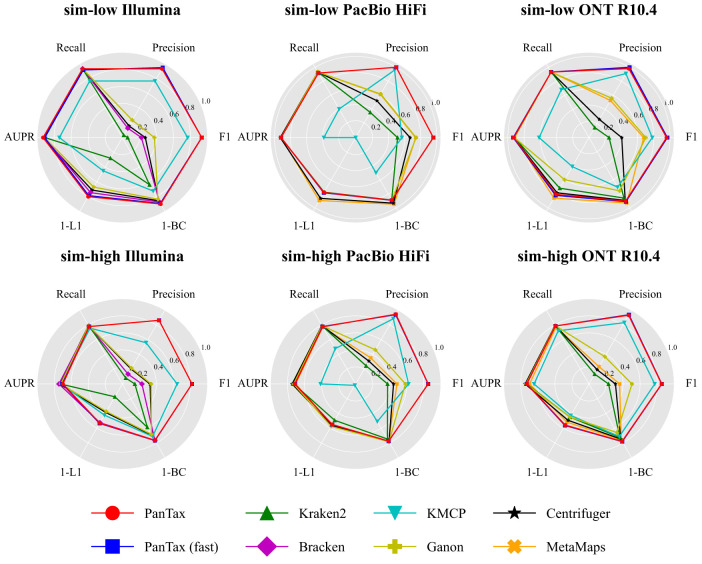
Benchmarking results of strain-level taxonomic profiling on the simulated data sets (sim-low and sim-high). The *upper* and *lower* panels display the sim-low and sim-high data sets, respectively, for each of the three sequencing read types. To visualize all metrics consistently (i.e., with higher values indicating better performance), we present the 1-L1 distance and 1-BC distance. (AUPR) Area under the precision-recall curve.

Figure 2 presents the benchmarking results. For the sake of a clear presentation, Figure 2 only focuses on the most important metrics. For detailed results, referring to all metrics, please see Supplemental Tables S2 and S3, corresponding to the two data sets sim-low and sim-high. In terms of taxon identification, when compared with all other tools, PanTax achieves high precision across all sequencing data types, including Illumina, PacBio HiFi/CLR, and ONT R9.4.1/R10.4. Specifically, for Illumina, PacBio HiFi, PacBio CLR, and ONT R10.4 data, PanTax achieves the highest precision (sim-low/sim-high: 0.933/0.863, 1.000/0.945, 0.982/0.927, 0.982/0.932) across both sim-low and sim-high data sets. On both sim-low and sim-high data sets (ONT R9.4.1), KMCP achieves the highest precision (1.000/0.950) at the cost of very low recall (0.317/0.362), whereas PanTax achieves the second highest precision (0.982/0.924) while maintaining comparable recall with other methods. Overall, PanTax achieves the highest F1 score for both sim-low and sim-high data sets across all sequencing data types, while maintaining comparable AUPR compared to other tools. Notably, PanTax (fast) demonstrates performance comparable to that of PanTax. However, for data sets with high sequencing error rates, such as PacBio CLR and ONT R9.4.1, the recall of PanTax (fast) tends to decrease. This is likely due to the “ultrafast genome querying” step, which applies relatively high ANI filtering, leading to missed detection of certain strains in noisy reads. For evaluating taxonomic abundances, PanTax (0.100/0.235) demonstrates superior performance in terms of BC distance for Illumina data sets, outperforming other methods across both simulated data sets. On sim-low data sets (PacBio HiFi, ONT R10.4), MetaMaps achieves the best BC distance, but it performs worse than PanTax on PacBio CLR and ONT R9.4.1 data. PanTax achieves the second best BC distance on the ONT R10.4 sim-low data sets. Conversely, on sim-high data sets (PacBio CLR, ONT R9.4.1/R10.4), PanTax achieves the best BC distance performance compared with other methods. Ganon performs the best for BC distance on the sim-high data set (PacBio HiFi). Notably, PanTax (fast) achieves performance comparable to PanTax across multiple distance metrics, including BC, L1, and L2 distances, as well as AFE and RFE.

To assess the strain-level performance of sylph, a recent excellent species-level metagenome profiling tool, we ran it on the sim-low and sim-high data sets. Sylph generally exhibits substantially lower recall than other tools (Supplemental Fig. S1), likely because its *k*-mer reassignment strategy lacks sufficient resolution for strain-level profiling.

#### Simulated data sets with introduced mutations

We also benchmarked PanTax alongside other tools on simulated data sets containing 0.1% and 1% mutations (sim-low-mut1, sim-high-mut1, sim-low-mut2, and sim-high-mut2). The overall trends remain consistent with the original simulations. PanTax retains F1 score across all sequencing technologies for both mutation levels, while its BC distance remains highly competitive (Supplemental Fig. S2; Supplemental Tables S4–S7). Detailed descriptions are available in Supplemental Note S2.

#### Real data sets

To assess performance on the Zymo1 and Zymo2 mock community data sets, we specifically constructed a new reference database to encompass most of the strains in Zymo1. This reference database includes only eight species, but each species consists of a greater number of strains selected from 34,697 complete genomes (Supplemental Methods S2) in RefSeq. The number of strains per species is limited to 80 due to aligner performance constraints. All benchmarking tools use this reference database to ensure a fair comparison.

**Figure 3.**
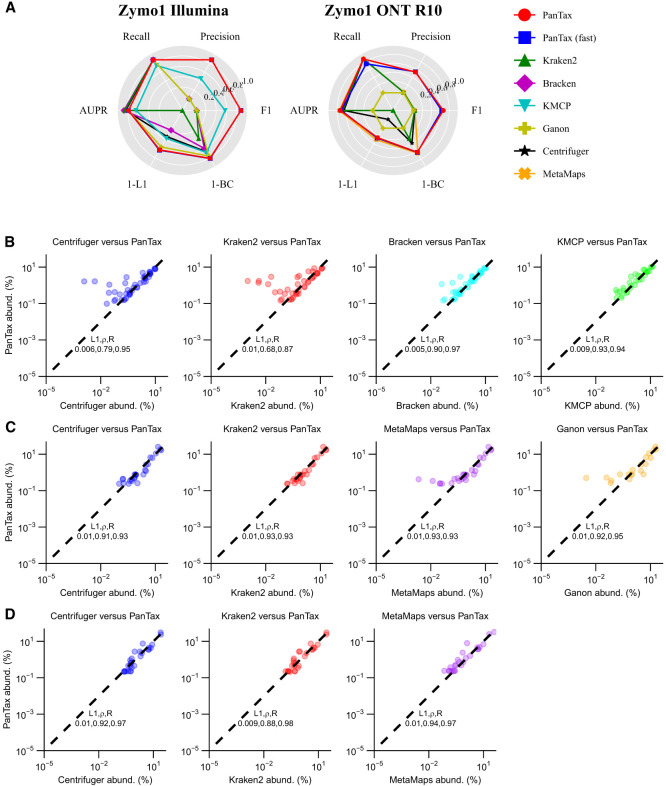
Strain-level taxonomic profiling across multiple species in real data sets. (*A*) Performance on the Zymo1 mock community. To visualize all metrics consistently (i.e., with higher values indicating better performance), we present the 1-L1 distance and 1-BC distance. (*B*–*D*) Performance on real human gut metagenomes: PD human gut (Illumina), omnivorous human gut (PacBio HiFi), and healthy human gut (ONT). For all three data sets, the relative taxon abundance correlation between PanTax and other competitive profilers was computed. The comparisons included mean L1 distance, Spearman's correlation, and Pearson's correlation. Note that we failed to run Ganon and KMCP on the healthy human gut (ONT) data set because it was primarily designed for short reads. (AUPR) Area under the precision-recall curve.

For Zymo1 (Fig. 3A; Supplemental Table S8), the strain-level taxonomic profiling results reveal that PanTax (0.875/0.467/0.538) significantly outperforms other methods in terms of precision on Illumina, ONT R9.4.1, and R10 data. Furthermore, PanTax achieves the highest F1 score across Illumina, ONT R9.4.1, and R10 data. Bracken achieves the best AUPR on Illumina data, whereas Kraken2 obtains the best AUPR (0.858) on ONT R9.4.1 data, and MetaMaps achieves the best AUPR (0.751) on ONT R10 data. Nonetheless, PanTax also achieves the second-best AUPR (0.729) on ONT R10.4 data. Regarding the evaluation of taxonomic abundances, PanTax shows the lowest BC distance on Illumina data, whereas MetaMaps achieves the lowest BC distance on ONT data. Additionally, PanTax demonstrates the lowest L2 distance across various data types, including Illumina and ONT. For Zymo2 (Supplemental Table S8), PanTax achieves the highest precision and F1 score on ONT R9.4.1 data and ranks second in BC distance. On the Illumina data set, PanTax performs competitively, ranking second in F1 score and third in both AUPR and BC distance, closely trailing KMCP and Ganon. Detailed discussions are provided in Supplemental Note S3.

To evaluate the feasibility of PanTax on real metagenomic data, we assessed its performance alongside several leading metagenomic profilers (according to BC distance in our simulated and mock community data sets) using human gut microbiome data sets, including PD human gut (Illumina), omnivorous human gut (HiFi), and healthy human gut (ONT) data sets. PanTax was compared with Centrifuger, Kraken2, Bracken, and KMCP on the Illumina data set, whereas comparisons on the HiFi and ONT data sets included Centrifuger, Kraken2, Ganon, and MetaMaps. Given the absence of ground truth for real human gut metagenomes, we focused on comparing the relative taxon abundances of commonly detected strains across PanTax and other profilers. As shown in Figure 3B–D, PanTax demonstrates a strong correlation with other state-of-the-art metagenomic profilers in terms of taxon abundance. Specifically, the Pearson correlation coefficient reaches 0.97 with Bracken on the Illumina data set, 0.93 with MetaMaps on the HiFi data set, and 0.97 with MetaMaps on the ONT data set. Notably, Bracken and MetaMaps are among the most competitive methods across most simulated and mock community data sets. PanTax (fast) showed similar results (Supplemental Fig. S3). In summary, these results highlight the capability of PanTax to perform strain-level taxonomic profiling on real, complex metagenomic samples.

#### Spiked-in data sets

**Figure 4.**
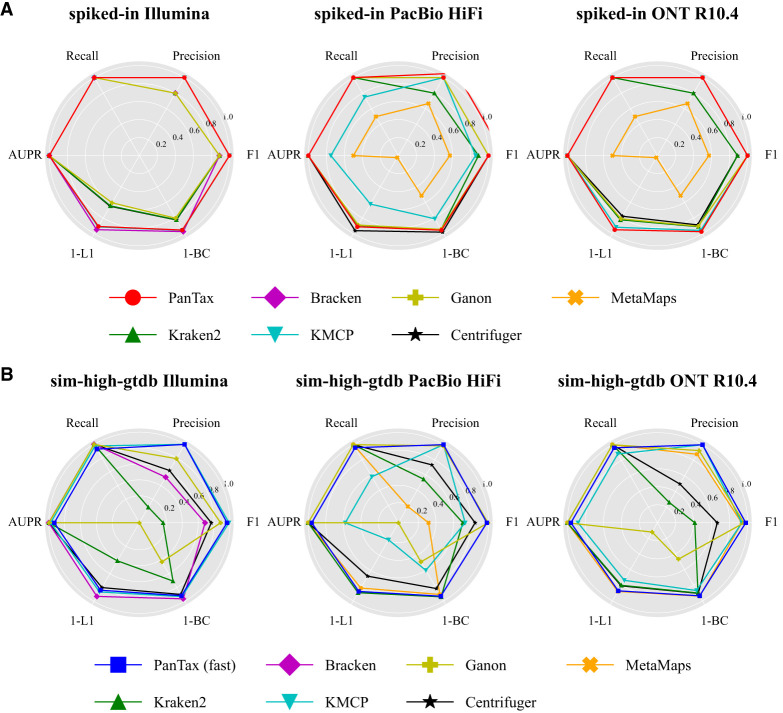
Benchmarking results of strain-level taxonomic profiling. (*A*) Profiling for spiked-in data sets. (*B*) Profiling for simulated data sets (sim-high-gtdb) using the GTDB reference database. To visualize all metrics consistently (i.e., with higher values indicating better performance), we present the 1-L1 distance and 1-BC distance. (AUPR) Area under the precision-recall curve.

Figure 4A presents the benchmarking results for strain-level taxonomic profiling on the spiked-in metagenomic data sets. For Illumina data, PanTax and KMCP achieve the highest precision, recall, F1 score, and AUPR (all equal to 1.0). Bracken performs best in terms of abundance estimation, as indicated by metrics such as BC/L1/L2 distances, AFE, and RFE, although PanTax shows only slightly worse performance than Bracken and KMCP. For HiFi data, PanTax, Ganon, Centrifuge, and Centrifuger achieve the highest precision, recall, F1 score, and AUPR (all equal to 1.0). Centrifuger excels in abundance estimation, but PanTax still outperforms Ganon in this aspect. Most importantly, for handling noisy reads, such as those from PacBio CLR and ONT R9.4.1/R10.4 data, PanTax substantially outperforms all other tools across all metrics. As these spiked-in data sets reflect both strain diversity (with an eight-strain mixture) and the complexity of real metagenomic samples, the results demonstrate the advantages and robustness of PanTax for metagenomic profiling on complex, real-world samples. Detailed results for all metrics can be found in Supplemental Table S9.

### Strain-level taxonomic profiling for single species

We note that some metagenomic tools for strain-level taxonomic profiling, such as Centrifuger, have the capability to perform strain-level profiling across multiple species. In contrast, other tools such as StrainScan, StrainGE, and StrainEst are specifically designed for single-species profiling of short reads. Subsequently, We compared PanTax’s profiling against three other tools in the single-species strain-level profiling tasks.

#### Simulated data sets: S. epidermidis strain mixtures (three strains, five strains, and 10 strains)

**Figure 5.**
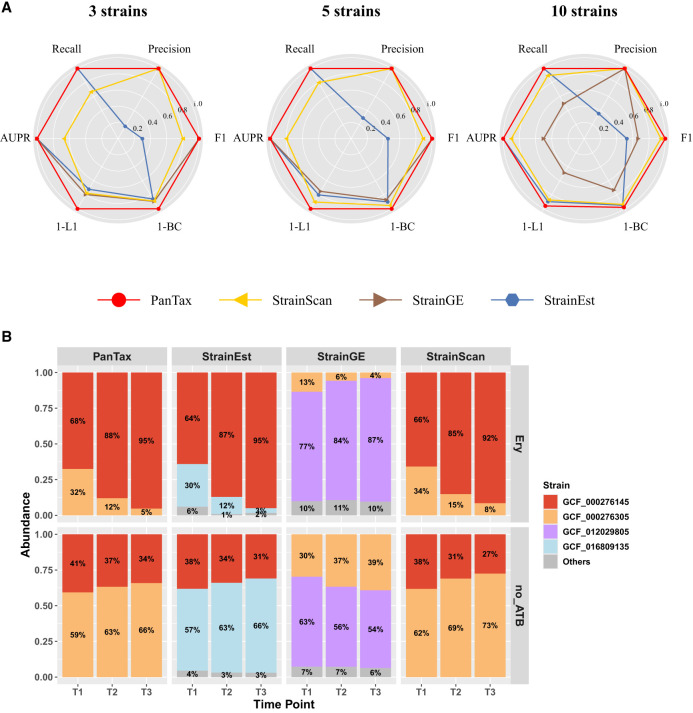
Benchmarking results of single species strain-level taxonomic profiling. (*A*) Simulated data sets (*S. epidermidis* strain mixtures: three strains, five strains, and 10 strains). (AUPR) Area under the precision-recall curve. To visualize all metrics consistently (i.e., with higher values indicating better performance), we present the 1-L1 distance and 1-BC distance. (*B*) Real data sets: two-cultured *S. epidermidis* strain mixtures. The *x* -axis represents time points, and the *y* -axis shows the relative taxon abundance of each identified strain. The two strains (marked in red and yellow) were mixed in equal proportions (1:1) and cultured under two conditions: one with erythromycin treatment (Ery) and one without antibiotics (no_ATB). The *upper* and *lower* panels display the strain abundances predicted by four tools under Ery and no_ATB, respectively.

For the simulated data sets, we selected *S. epidermidis* (species taxid: 1282) as an example. It is important to note that when dealing with a single species, PanTax faces challenges in constructing a pangenome from an excessive number of individual genomes (e.g., >100 ). Therefore, we applied clustering techniques to remove highly similar (redundant) genomes before constructing the pangenome. Other methods also incorporate built-in techniques for eliminating redundant genomes, ensuring the fairness of our comparison. First, we chose all complete genomes (147) of *S. epidermidis* from the RefSeq database and subsequently obtained 70 nonredundant genomes (strains) of this species using single-linkage clustering with a threshold of ANI ≤ 99.9%. These genomes were subsequently utilized to construct the reference pangenome graph for this species. PanTax achieved nearly perfect performance on all three data sets, attaining 100% precision, recall, and AUPR, as well as nearly zero BC distance (Fig. 5A; Supplemental Table S10). This significantly outperforms other state-of-the-art approaches, such as StrainScan, StrainGE and StrainEst.

#### Real data sets: antibiotic-resistant S. epidermidis strain mixtures (two strains)

We selected a total of 1395 *S. epidermidis* genomes (including both complete and incomplete genomes) from the RefSeq database, as these two strains are not included in the complete reference genomes. To handle more reference genomes in this case, we employed a two-stage redundancy removal strategy (Supplemental Note S4) to remove redundant genomes, followed by the construction of the reference database for PanTax.

We compared PanTax with other tools that perform strain-level taxonomic classification for these *S. epidermidis* two-strain mixtures, with the results shown in Figure 5B. These mixtures consist of two *S. epidermidis* strains, among which GCF_000276305.1 is not resistant to the antibiotic erythromycin, whereas GCF_000276145.1 exhibits high resistance to erythromycin, and their relative abundances vary at different time points, as reported in the original study (Emiola et al. 2020). GCF_000276305.1 was consistently the dominant strain in the no_ATB group, whereas GCF_000276145.1 was the dominant strain in the Ery group at each time point. The results demonstrate that both PanTax and StrainScan correctly identified the two strains in all six samples. Furthermore, PanTax and StrainScan showed similar relative abundance estimations. StrainGE and StrainEst returned two main correct representative strains. However, the relative proportions of the two dominant strains detected by StrainGE in the no_ATB group do not match the ground truth. StrainGE identified GCF_012029805 as the dominant strain instead of GCF_000276305 in the no_ATB group. StrainEst consistently detected false-positive minor strains.

### Effects of reducing sequencing coverage

To study the effects of reducing sequencing coverage, we randomly subsampled 1/2 and 1/5 of the reads from the “sim-low” data sets, naming these subsets “sim-low-sub1” and “sim-low-sub2”, respectively. After subsampling, the lowest-coverage strain in sim-low-sub1 data sets is approximately 0.5× , whereas in sim-low-sub2 data sets it is approximately 0.2× . Supplemental Tables S11 and S12 present the strain-level taxonomic profiling results for the sim-low-sub1 and sim-low-sub2 data sets, respectively. For sim-low-sub1, PanTax significantly outperforms other methods (with the exception of KMCP) in strain-level precision across most sequencing data types, including Illumina, PacBio HiFi, and ONT R9.4.1/R10.4. However, KMCP exhibits considerably lower recall, resulting in PanTax or PanTax (fast) achieving the highest F1 score across these sequencing technologies. Additionally, PanTax demonstrates comparable or superior performance in taxonomic abundance estimation, as indicated by metrics such as L2 distance, BC distance, AFE, and RFE. A similar trend is observed for sim-low-sub2 (Supplemental Note S5). As sequencing coverage decreases, PanTax exhibits lower recall but higher precision in strain identification. Additionally, its taxonomic abundance estimation becomes less accurate. In summary, these experiments demonstrate that PanTax or PanTax (fast) is the superior choice of approach in particular when dealing with low coverage (components of) metagenomes. Notably, PanTax shows a decrease in performance for strains with low coverage (<1×).

### PanTax is effective for larger reference metagenome databases

To demonstrate that PanTax is capable of handling larger reference metagenome databases, such as the GTDB, we specifically developed an optimized version, PanTax (fast), as detailed in the Methods section. We benchmarked PanTax (fast) against other approaches using the sim-high-gtdb data sets (as reflecting complex metagenomes) across all five sequencing data types (using GTDB:206273). The results are shown in Figure 4B (detailed values of each metric are provided in Supplemental Table S13). For Illumina data, PanTax (fast) achieves the second-best precision (0.998), which is only slightly lower than KMCP (1.0). Although PanTax (fast) shows lower recall compared to other methods, its BC distance is significantly better than tools like Ganon, Centrifuge, and Kraken2. For the four long-read data sets, KMCP is the only competitor that outperforms PanTax (fast) in precision, albeit with a very slight advantage(0.001 to 0.002). However, PanTax (fast) substantially surpasses KMCP in other metrics, including recall (0.079 to 0.45 higher), AUPR, L1, and BC distances. In summary, PanTax (fast) demonstrates comparable overall performance to KMCP on Illumina data sets but substantially outperforms all other methods on long-read data sets.

### Benchmarking PanTax against long-read metagenome assemblers for strain-level profiling

With the rapid development of long-read metagenome assemblers, such as metaMDBG (nanoMDBG) (Benoit et al. 2024), (meta)flye (Kolmogorov et al. 2020), hifiasm-meta (Feng et al. 2022), and myloasm (Shaw et al. 2025), the quality and efficiency of metagenomic assemblies have been substantially improved. These advances make it feasible to perform strain-level profiling directly from metagenome-assembled genomes (MAGs). To investigate the performance gap between PanTax and assembly-based profiling approaches, we conducted benchmarking on seven representative data sets: sim-low PacBio HiFi, sim-low ONT R10.4, sim-high PacBio HiFi, sim-high ONT R10.4, sim-high-mut2 PacBio HiFi, sim-high-mut2 ONT R10.4, and Zymo1 ONT R10.4. Currently, no dedicated tools exist for performing strain-level profiling directly from MAGs. Therefore, we implemented a custom assembly-based profiling pipeline (Supplemental Methods S10).

The benchmark results are summarized in Supplemental Table S14. On simulated data sets, assemblers achieve recall rates comparable to those of PanTax; however, PanTax consistently outperforms assembly-based approaches in precision and distance-based metrics (e.g., BC distance). On the real data set (Zymo1 ONT R10.4), metaMDBG exhibits poor precision (PanTax 0.538/metaMDBG 0.027), whereas myloasm shows markedly low recall (PanTax 0.875/myloasm 0.250).

These results do not suggest that genome assembly is inherently unsuitable for strain-level analysis; rather, they point to the need for more robust and accurate strategies to interpret MAGs for profiling. Despite the high resolution achieved by long-read assemblies, homologous regions are often collapsed during assembly, which complicates accurate abundance estimation. A dedicated and reliable method is still necessary to deconvolve such regions and support accurate strain-level profiling from assembled genomes.

### Effects of divergence versus ratio of abundances

To explore the impact of divergence and abundance disparities on strain identification in mixed samples, we simulated multiple mixtures comprising two strains. Similarly, we used the reference database for *S. epidermidis* (species taxid: 1282) which was previously constructed for the *S. epidermidis* simulated data sets and selected six strains from it to perform the experiment. These mixtures encompassed various combinations of ANI values of 96.8%, 97%, 98%, 99%, and 99.8% (with 99.8% representing the most challenging scenario and 96.8% the least) and abundance ratios of 1:1, 1:3, 1:5, and 1:10. Our experiments centered on Illumina reads, and we subsequently assessed performance of PanTax using F1 score, AUPR, and L2 distance for each possible combination of divergence and abundance ratio (Supplemental Fig. S4). Analysis of the F1 score and AUPR indicates that PanTax successfully identifies both true strains in all scenarios. Regarding L2 distance, although PanTax accurately estimates the relative abundances of each strain in most cases, it exhibits biased abundance estimations for the most challenging scenario (ANI = 99.8%).

### Robustness of PanTax across reference diversity benchmarks

To evaluate the robustness of PanTax under increasing reference diversity, we performed benchmarks using simulated (SimRef) and real (Zymo1) data sets with reference databases of progressively greater species and strain diversity (Supplemental Methods S11). As the reference expanded from a perfect match set (SimRef1) to full genus-level coverage (SimRef5), only slight changes were observed in both F1 score and BC distance (Supplemental Table S15). For the SimRef data sets across all five sequencing data types, based on simulated reads from 60 *Streptococcus* strains, performance remains stable when the reference was expanded to include up to 596 strains across 83 species. Across all long-read data sets, PanTax achieved >99% of reads with MAPQ 60 (optimal mapping quality), confirming its robustness to increasing reference diversity and graph complexity (Supplemental Methods S12; Supplemental Fig. S5; Supplemental Note S6). Similarly, for the Zymo1 data set, PanTax maintains comparable accuracy across reference sets ranging from reduced strain subsets to the complete Zymo reference. These results demonstrate that PanTax preserves high accuracy and low compositional deviation even when the reference database is scaled to include substantially more species and strains, highlighting its robustness for large and diverse pangenome references in real-world metagenomic analyses.

### PanTax performance under increasing strain quantity and genome similarity

To evaluate the scalability of PanTax (Supplemental Methods S13), we assessed its performance under two distinct scenarios: increasing the number of reference genomes (strain quantity scaling) and increasing genomic similarity among reference strains (genome similarity scaling). These experiments aimed to determine how PanTax handles larger and more similar pangenome references, and to identify potential limits in strain-level profiling accuracy. In the strain quantity scaling experiments (Supplemental Fig. S6A), for Illumina data, the F1 score fluctuates narrowly between 0.982 and 0.988, and the BC distance ranges from 0.075 to 0.085. For PacBio HiFi data, the F1 score remains 0.988–0.990 and BC distance 0.071–0.090. These results primarily reflect the effect of increasing the number of species, and performance changes are minimal. In the genome similarity scaling experiments (Supplemental Fig. S6C), for Illumina data, the F1 score remains 1.0, perfectly predicting all genomes, while BC distance gradually increases with strain number. For PacBio HiFi data, the F1 score stays 1.0 for 1–30 strains, with only minor decreases at 40 (0.974) and 50 (0.980) strains. BC distance also increases with strain count, indicating slightly higher abundance estimation errors. These results suggest that as strain similarity rises, PanTax performance may fluctuate and slightly decline. Overall, in both scaling dimensions, PanTax maintains high robustness when applied to multispecies reference sets with dozens to hundreds of genomes per species. For species with very large numbers of reference genomes (e.g., >100 ), pangenome graph construction requires substantial computational resources, and the presence of “super bubbles” slows alignment significantly, with the exact threshold varying by species. Although only minor performance fluctuations or declines are observed, this scenario does not yet reach the true computational limits of the method. Challenges in pangenome construction and alignment likely constitute the primary constraints when probing PanTax’s scaling boundaries. Further assessment using real data sets is essential to fully characterize these limitations in the future.

### Runtime and memory usage evaluation

Benchmarking results for runtime and memory usage (Supplemental Fig. S7; Supplemental Tables S16–S18) indicate that PanTax generally requires longer database construction and profiling times, as well as higher memory consumption in its default mode. In contrast, PanTax (fast) substantially improves computational efficiency, achieving comparable or superior performance relative to most methods across both short- and long-read data sets. On short-read data sets, PanTax (fast) was slightly slower than Ganon and Bracken but remained competitive in runtime while consuming the least memory. For long-read data sets, it demonstrates a favorable trade-off between speed and memory, requiring minimal RAM (7–30 GB) while operating within 1–10× of the fastest methods depending on the data set. Even for single-species profiling (Supplemental Table S19), PanTax (fast) required moderate computational resources (around 27 CPU hours and 20 GB RAM), highlighting a balanced compromise between accuracy and efficiency. Detailed descriptions are available in Supplemental Note S7.

## Discussion

We have introduced PanTax, an approach that classifies the contents of metagenomes in terms of their taxa. PanTax accepts all classes of popular sequencing reads as input, and determines the organisms that make part of the metagenome at the level of their strains. Although classification of the contents of metagenomes at the level of species had reached a satisfying level of maturity in the literature, determining presence and abundance of strains of species in metagenomes has still remained in its infancy. The high similarity among strains makes accurate profiling at strain-level resolution particularly challenging. Correspondingly, PanTax addresses this important open challenge.

Key to its success is that PanTax, to the best of our knowledge, is the first approach to use pangenome graphs as whole-genome reference systems, rather than linear genomes. Unlike linear references, pangenome graphs capture the full genetic diversity of a collection of genomes at the strain level and organize them in an evolutionarily consistent manner, which eliminates ambiguities during classification. This is particularly advantageous for determining the strain or species origin of individual reads: a read aligned to the graph immediately identifies its strain and species, whereas alignment to multiple linear references requires complex statistical evaluation and may leave unresolved uncertainties. In addition, the unified alignment strategy is applicable to both high-accuracy short reads and noisy long reads, which alignment-free *k*-mer-based approaches cannot reliably guarantee. Based on the alignment results, strain abundance is further formulated as a linear programming problem, enabling computationally tractable strain-level profiling. This optimization problem-based strategy allows for utmost nuanced and accurate distinction between the strains of a species.

PanTax is based on variation graphs, a type of pangenome graph whose topological structure is the key factor that makes PanTax possible. In PanTax, the pangenome graph plays a central role at two critical levels: (1) Efficient alignment and species-level binning. We employ a super pangenome graph constructed by linearly merging pangenome graphs from multiple species. This unified reference structure enables direct species-level binning of reads after alignment and significantly reduces ambiguous alignments commonly associated with linear references. (2) Mathematical formulation for path abundance optimization (see Methods for details). We formulate strain abundance estimation as a path abundance optimization problem. Each strain corresponds to a unique path in the graph, and a linear programming model relates node coverage to path abundance under graph-topological constraints. Furthermore, strain-specific nodes within the graph are leveraged to filter out false positives, improving estimation accuracy.

Beyond delivering decisive progress with respect to the characterization of the strain content of metagenomes, PanTax also delivers relevant practical advantages. Namely, PanTax is not prone to biases relative to preselected genomic regions, PanTax can process all popular types of reads, it can handle multiple species simultaneously, and it can integrate custom databases.

Our extensive benchmarking results demonstrate that PanTax drastically outperforms state-of-the-art approaches, primarily evidenced by its significantly higher F1 score at strain level, while maintaining comparable or better performance in other aspects across various data sets.

The main drawback of the original PanTax is its greater demand of computational resources, both in terms of time and memory, compared to other methods, which makes it challenging to process very large databases, such as GTDB. The key steps involved are the construction of pangenome graphs and the loading of them into memory. To address this limitation, we have also developed a fast version of PanTax by incorporating an ultrafast genome querying step before constructing the pangenome graphs. This approach drastically reduces the number of genomes in the reference database from which the graph is to be constructed, which significantly decreases the time and the memory needed to construct and load the pangenome graphs into main memory. Consequently, PanTax, in its fast version, requires comparable or even fewer computational resources than other approaches. Benchmarking experiments demonstrate that the fast version of PanTax approaches the taxonomic profiling performance rates of the full, default version of PanTax across the full board of data sets; in fact, it even exhibits slight advantages in some cases.

In summary, PanTax is compatible with short reads (e.g., Illumina), both noisy PacBio CLR and accurate PacBio HiFi, as well as ONT, both noisy R9.4 and the more accurate R10.4 (error rate about 2%) sequencing data. It supports classification at the strain level (and, of course, if desired, also at the level of species), for both single and multiple species, so establishes an approach that is universal with respect to all current scenarios of interest in metagenomics type experiments.

Further improvements of PanTax are conceivable, and they are promising. Currently, just like any other reference-based classification approach, PanTax is unable to generate taxonomic profiles for strains that because they are novel, are missing in the reference databases. Instead, PanTax reports the most similar strain recorded in the databases. Although this at least accurately determines the most likely closest relative, a preferable solution would be to dynamically update the species-specific pangenome graphs by incorporating hitherto unobserved genetic variation. From a larger perspective, this would support the detection of unknown strains, beyond just enhancing classification by removing mistaken hits.

## Methods

In the following, we provide the full range of methodological details involved in the steps in Figure 1.

### Pangenome graph–based reference database construction

When constructing our default pangenome-based reference database (“Graph representation of reference databases” in Fig. 1), we focus on bacteria as the primary object of interest in the majority of metagenomics studies. We are aware that in metagenomics studies also viruses, archaea, fungi, or plasmids (and so on) can be in the major focus of attention. To account for this, one can, mutatis mutandis, readily extend our methodology to other organisms. Users can flexibly feed their customized databases as input to PanTax, instead of using the provided default databases. Once databases are provided, everything else follows the identical workflow. In the following, numbers and names of “Steps” correspond to the numbering and naming of procedures in Figure 1.

#### Step 1: Select high-quality genomes for species

We selected high-quality, complete bacterial genomes from NCBI RefSeq (Release 219, July 18, 2023) (O’Leary et al. 2016), with plasmid sequences excluded. To remove redundancy, pairwise average nucleotide identity (ANI) values are calculated for each species using FastANI (Jain et al. 2018), and a graph-based clustering approach (Supplemental Methods S16) is applied at 95% and 99.9% ANI thresholds to identify representative genomes. To balance sensitivity with computational efficiency, a maximum of 10 representative genomes per species was retained, resulting in a reference database (RefSeq:13404) comprising 13,404 strains from 8778 species (Supplemental Fig. S8). Furthermore, genomes were retrieved from the GTDB (Release R220) using genome_updater (https://github.com/pirovc/genome_updater), with a cap of 100 genomes per species to manage computational resources. After applying the same redundancy-reduction procedure, we constructed a refined GTDB reference database (GTDB:206273) containing 206,273 strains. Detailed procedures are described in Supplemental Methods S2.

To handle large-scale databases such as GTDB, we develop a fast mode of PanTax. The only difference between PanTax (fast) and the original PanTax is the inclusion of an additional step, “ultrafast genome querying.” This step is implemented using the query module from sylph, which estimates the ANI between strain genomes in the database and the metagenomic sample. Strains with ANI above the threshold (default: 99%) are retained for graph construction. This enables rapid filtering of strains, reducing both the number and the complexity of pangenome graphs that need to be constructed.

#### Step 2: Construct a pangenome graph for each species

For each species, a variation graph, which is a widely used representation of a pangenome, is constructed from strain genomes using PGGB (Garrison et al. 2024). In this graph, nodes correspond to genomic segments, edges represent adjacencies observed in at least one strain genome, and each strain is encoded as a path. For species represented by a single genome, the genome sequence is divided into 1024-bp fragments and converted into a graph of the same structure.

PanTax employs a two-stage construction strategy. Species-specific pangenome graphs are first generated independently and are subsequently combined into a unified multispecies pangenome graph. This approach markedly reduces computational time and memory requirements compared with constructing a global pangenome directly from all genomes and is conceptually comparable to the chromosome-based construction strategy used in the draft human pangenome reference (Liao et al. 2023a). The unified graph allows each sequencing read to be classified through a single alignment to the entire graph, eliminating the need for separate alignments against thousands of individual species references. Detailed procedures are provided in Supplemental Methods S3.

### Species-level taxonomic classification

#### Step 3: Sequence to graph alignment

Sequencing reads are then mapped to the merged pangenome graph using existing, approved and efficient sequence-to-graph aligners. For short reads, we employ Giraffe (Sirén et al. 2021), as an integral part of the vg toolkit (Garrison et al. 2018). This aligner requires prior construction of graph-based indexes, including the graph Burrows–Wheeler transform (GBWT) index (Supplemental Methods S4). For long reads, we make use of GraphAligner (Rautiainen and Marschall 2020).

#### Step 4: Species-level taxonomic binning

Taxonomic binning involves the allocation of sequencing reads to distinct taxonomic groups. In this procedure, we assign each mapped read to a species-specific pangenome graph, utilizing the results of the sequence-to-graph alignment resulting from aligning reads with the merged pangenome graph. When a read aligns to multiple graphs (within the unified, merged pangenome graph), we only retain the optimal alignment, thereby ensuring that each read corresponds to a single species. We again note that the optimal alignment is easy to determine precisely because the merging of graphs implies that different alignment scores can be put into mutual context. Subsequently, we assess whether individual species need to be flagged as false positives. That is, although the species receives a high score, the species does not make part of the metagenome. To achieve this, we perform a postalignment filtering (Supplemental Methods S5) step based on MAPQ (mapping quality). Only species supported by sufficient high-confidence read alignments are retained. It remains to mention that reads may fail to align with the pangenome graph, which potentially indicates the presence of novel strains (not necessarily from novel species, although that is possible as well) in the metagenomic sample under analysis. We discard such unmapped reads from further consideration in the subsequent analysis.

#### Step 5: Species-level taxonomic profiling (optional)

For species *i* , we divide the total base count of the reads that optimally aligned with species *i* via the read-to-graph alignment by the average length of the strain-specific genomes that contributed to constructing the pangenome graph for species *i* , which we refer to as *normalized read coverage c_*i*_* in the following. Subsequently, we calculate the relative (taxon) abundance of species *i* as the ratio $c_{i}/\sum_{i\in T}c_{i}$, where *T* is the set of species reported in the previous step (e.g., resulting from excluding potentially false positives when dealing with short reads, before outputting all species referred to as *T* here). The output of species-level classification is optional.

### Strain-level taxonomic classification

#### Step 6: Path abundance optimization

To achieve strain-level classification, we formulate a *path abundance optimization (PAO)* problem for each species-specific pangenome graph. Following Baaijens et al. (2020), the strain abundance *a*_*p*_ is estimated by minimizing the difference between the observed node coverages (derived from read alignments) and the coverages implied by strain genome paths. The convex optimization problem is solved using Gurobi (https://www.gurobi.com), although open-source solvers such as HiGHS (Huangfu and Hall 2018), Cbc (Lougee-Heimer 2003), and GLPK (http://www.gnu.org/s/glpk/glpk.html) are also compatible (Supplemental Methods S7; Supplemental Table S20). We perform two iterations of PAO, each preceded by a filtering step to reduce false positives. In the first filtering step, we compute *f*_strain_ , the fraction of strain-specific triplet nodes (unique consecutive node triples) covered by reads, and retain strains with *f*_strain_ ≥ 0.3 for short reads or *f*_strain_ ≥ 0.5 for long reads. In the second filtering step, we introduce the metric *d*_strain_ , which quantifies the divergence between the PAO-predicted strain abundance *a*_*p*_ and the independent triplet-based estimate *a*_*p*,triplet_ . Strains with *d*_strain_ ≤ 0.46 are retained, whereas those with 0.46 < *d*_strain_ ≤ 0.6 are rescued if they satisfy the empirical criterion *r*_strain_ = *b*_strain_ × *f*_strain_ ≥ 0.85 . *b*_strain_ is defined as the base coverage of a strain. Finally, we apply the second PAO iteration to obtain the final strain abundances. For rescued strains, the lower value between *a*_*p*_ and *a*_*p*,triplet_ is reported as their absolute abundance (i.e., the average coverage depth). The complete definitions, parameter settings, and computational details are provided in Supplemental Methods S6, whereas sensitivity analyses of *f*_strain_ , *d*_strain_ , and *r*_strain_ are described in Supplemental Methods S8, with corresponding results shown in Supplemental Figs. S9–S11.

#### Step 7: Strain-level taxonomic profiling

In the strain-level profiling step, we introduce a divergence metric *d*_species_ to assess the consistency between the estimated absolute abundance of each species and the total abundance of its assigned strains. Species with *d*_species_ > 0.2 are filtered out as potential false positives, which typically arise from reads misaligned to nonexistent species through shared genomic regions. If the summed strain abundances exceed the species abundance, they are proportionally scaled to ensure consistency. Finally, all strains are aggregated, and their relative abundances are estimated by normalizing absolute abundances. Detailed definitions are provided in Supplemental Methods S9.

## Simulated data sets

The primary simulated data sets used to reproduce the results in this study are available at Zenodo (https://doi.org/10.5281/zenodo.16885808). For the additional simulated data sets, the corresponding configuration files are also provided at Zenodo and can be used with CAMISIM to regenerate the data.

## Code availability

The source code of PanTax is GPL-3.0 licensed, and publicly available at GitHub (https://github.com/LuoGroup2023/PanTax) and as Supplemental Code. To ensure reproducibility, we provide a Code Ocean capsule with minimal runnable examples of the software’s core functionality (https://codeocean.com/capsule/8284706/tree/v1). All custom scripts necessary to reproduce the benchmarking experiments can be accessed at GitHub (https://github.com/LuoGroup2023/pantax-eval) and as Supplemental Code.

## Competing interest statement

We, the authors, have a patent application (No. 2024110965476) related to this work, and we confirm that there are no patents held by immediate family members that may potentially conflict with the content of this paper. The authors declare no other competing interests.

## Supplemental Material

Supplement 1

Supplement 2

Supplement 3

Supplement 4

Supplement 5

Supplement 6
