# Supplementary material for: Strain-level metagenomic profiling using pangenome graphs with PanTax
Source: Genome Res. 2026 Feb;36(2):405–20. doi: 10.1101/gr.280858.125 (PMC12863173; doi:10.1101/gr.280858.125)
Supplement: Supplement 1 [file Supplemental_Notes.pdf]

Strain-level metagenomic profiling using pangenome graphs with PanTax

Wenhai Zhang[1,2,†], Yuansheng Liu[3,†], Guangyi Li[2,†], Jialu Xu[2], Enlian Chen[2], Alexander Schönhuth[4,*] Xiao Luo[1,2,*]

[1] Hunan Research Center of the Basic Discipline for Cell Signaling, Hunan University, Changsha, China
[2] College of Biology, Hunan University, Changsha, China
[3] College of Computer Science and Electronic Engineering, Hunan University, Changsha, China
[4] Faculty of Technology, Bielefeld University, Bielefeld, Germany
[†]These authors contributed equally to the work.
[*]To whom correspondence should be addressed.
Email: aschoen@cebitec.uni-bielefeld.de
Email: xluo@hnu.edu.cn

Supplemental Notes

S1 Remarks on evaluating alternative methods

To ensure a rigorous comparison, we have comprehensively selected a variety of representative metagenomic profilers (Supplemental Table S1) and evaluated them concurrently. A selection of tools was chosen for evaluation based on the following criteria: open-source availability, active maintenance and/or highly used, acceptable execution time, the ability to create custom databases containing nucleotide sequences, the capability to perform strain-level binning and/or profiling, and good performance in strain classification tasks. Given the principled differences between short and long reads, we conducted comparisons separately for these two types of reads.

For strain-level profiling of multiple species, we ran representative tools such as Centrifuge, Centrifuger, Kraken2, Bracken, Ganon and KMCP on short-read sequencing datasets, while we ran Centrifuge, Centrifuger, Kraken2, Ganon, KMCP and MetaMaps on the long-read sequencing datasets. Note that we excluded CAMMiQ from our benchmarking evaluations because runs ended in segmentation faults in all our attempts. We ran PanTax in two modes: the default mode and the fast mode. The default mode uses a pre-built pangenome, providing a comprehensive strain profiling. However, the pre-built pangenome requires substantial resources in terms of runtime and memory usage. In contrast, the fast mode first uses efficient strategies (i.e. the query module in sylph)

to filter genomes, and then constructs a pangenome tailored to the given samples. This mode greatly reduces resource consumption while still yielding relatively accurate strain profiling results. As for strain-level profiling of single species, we compared with the state-of-the-art methods StrainScan, StrainGE and StrainEst, all of which are specifically designed for this task on short-read metagenomic data.

Note that it was observed that usage of default reference databases can introduce systematic biases when using taxonomic classifiers (Simon et al., 2019). To mitigate this effect, we utilized a unified set of reference genomes for all methods involved. For strain-level profiling in the multiple species experiments, all methods refer to either RefSeq:13404 or GTDB:206273, because one needs to include all strains into the reference databases for strain-level profiling. RefSeq:13404 encompasses 13,404 strains(8778 species), whereas GTDB:206273 contains 206273 strains (107205 species). We use RefSeq:13404 or GTDB:206273 for constructing pangenomes, because they list several strains for many species and represent commonly used taxonomic classification databases.

S2 Strain level taxonomic profiling for multiple species (Simulated datasets with introduced mutations)

For datasets with 0.1% mutations (sim-low-mut1 and sim-high-mut1), PanTax demonstrates notably high precision compared to other tools across all sequencing types, including Illumina, PacBio HiFi/CLR, and ONT R9.4.1/R10.4. Specifically, for Illumina, PacBio HiFi, PacBio CLR and ONT R10.4, PanTax achieves the highest precision (0.932/0.864, 1.000 /0.943, 0.949/0.920, 0.982/0.930) across both simulated datasets, while for ONT R9.4.1 datasets, PanTax achieves the second highest precision (0.966/0.921). In both sim-low-mut1 and sim-high-mut1 datasets (ONT R9.4.1), KMCP reaches the highest precision (1.000/0.957), but with a significant trade-off in recall, which is substantially lower. PanTax, on the other hand, achieves the second-highest precision (0.966/0.921) while maintaining comparable recall to other methods. In terms of overall performance, PanTax achieves the highest F1 score across all sequencing types for both sim-low-mut1 and sim-high-mut1 datasets, while maintaining similar AUPR to other tools. Regarding taxonomic abundance estimation, for Illumina datasets, PanTax (0.110/0.236) performs better in terms of BC distance than the other methods across both simulated datasets. On sim-low-mut1 datasets (PacBio HiFi, ONT R10.4), MetaMaps performs best in terms of BC distance but underperforms compared to PanTax on PacBio CLR and ONT R9.4.1 data. On sim-high-mut1 datasets (PacBio CLR, ONT R9.4.1/R10.4), however, PanTax achieves the best BC distance performance compared to the other methods.

For datasets with 1% mutations (sim-low-mut2 and sim-high-mut2), PanTax maintains the highest precision across all sequencing types for sim-low-mut2 and sim-high-mut2 datasets. In addition, PanTax achieves the highest F1 score for both datasets across all sequencing data types. In evaluating taxonomic abundances, for Illumina, PacBio CLR, and ONT R9.4.1 datasets, PanTax (0.101/0.236, 0.103/0.221, 0.085/0.221) outperforms the other methods in terms of BC distance across both simulated datasets. On sim-low-mut2 datasets (PacBio HiFi, ONT R10.4), MetaMaps achieves the best BC distance.

However, on sim-high-mut2 datasets (Illumina, PacBio CLR, ONT R9.4.1/R10.4), PanTax achieves the best BC distance performance. Ganon achieves the best BC distance on the sim-high-mut2 dataset (PacBio HiFi), but with only a slight advantage over PanTax. It is important to note that the comparison between PanTax (fast) and PanTax reveals similar patterns across most metrics, as observed in the original sim-low and sim-high datasets.

S3 Strain level taxonomic profiling for multiple species (Zymo2)

For Zymo2, KMCP achieves the best F1 score on the Illumina dataset, while PanTax ranks second. In terms of AUPR and BC distance, PanTax ranked third, only behind KMCP and Ganon. Notably, KMCP performs better on Zymo2 than on Zymo1. Its matched reference genomes require at least 10% high-confidence uniquely matched reads with strong k-mers support; we hypothesize that low sequencing depth or genome coverage reduces the number of false-positive k-mers from reference genomes, thereby enhancing KMCP's specificity. On the ONT R9.4.1 dataset of Zymo2, PanTax achieves the highest precision and F1 score, although its recall is slightly lower than other methods. In terms of BC distance, PanTax ranks second, only behind MetaMaps. MetaMaps demonstrates the best performance on BC distance and AUPR, indicating that correctly predicted strains are highly ranked in abundance. However, its precision is substantially lower than PanTax (PanTax: 0.667, MetaMaps: 0.019), suggesting a high false positive rate. These results underscore that while PanTax is affected by extremely low coverage genomes ($< 1\times$), it still achieves strong overall performance, especially in F1 score, on challenging datasets such as Zymo2.

S4 Two-stage redundancy removal strategy for single-species real datasets

In the first stage, we performed complete-linkage clustering using an ANI threshold of 99% to reduce redundancy, resulting in 69 non-redundant genome clusters. Representative genomes from these clusters were then selected as the reference genomes, marking the completion of the first redundancy removal stage.

In the second stage, we ran PanTax with less stringent parameter settings (f_{strain} set to 0.2) to filter out clusters where the representative genome was not identified by PanTax. We then merged all genomes from the remaining clusters and applied a more stringent ANI threshold of 99.9% for graph-based clustering approach (see Algorithm 1 in the Supplemental Methods) to further reduce redundancy, similar to the approach used for the previous simulated datasets. The difference is that all non-redundant genomes were selected, rather than the default number of 10 non-redundant genomes.

After completing these two stages of redundancy removal, we selected the non-redundant genomes as the final reference genomes to construct the pangenome graph.

S5 Effects of reducing sequencing coverage (sim-low-sub2)

PanTax or PanTax (fast) again outperforms other methods (except KMCP) in strain-level precision across all sequencing data types, including Illumina, PacBio HiFi/CLR, and ONT R9.4.1/R10.4. However, KMCP exhibits substantially lower recall, leading to a significantly reduced F1 score, particularly in long-read datasets. Regarding taxonomic abundance estimation, PanTax or PanTax (fast) achieves performance comparable to or slightly below that of the top-performing tools.

S6 PanTax maintains robust alignment accuracy across graph complexities and sequencing platforms

We further examined, using the SimRef references and corresponding simulated datasets, whether increasing reference diversity, and thus graph complexity, could impact read alignment quality in long-read datasets. Graph complexity was represented by the total number of graph nodes, which increased with reference size. Across all long-read sequencing technologies, PacBio HiFi reads consistently achieve a MAPQ score of 60, and over 99% of reads from ONT R10.4, ONT R9.4.1, and PacBio CLR also have MAPQ 60 (optimal mapping quality), with only a small fraction of reads falling between 0 and 60 (Supplemental Figure S5). These results indicate that neither increased graph complexity nor differences in long-read platforms substantially affect PanTax's strain-level profiling accuracy.

S7 Runtime and memory usage evaluation

Supplemental Tables S16–S17 present the runtime and peak memory usage of benchmarking tools using short-read (Illumina) and long-read datasets, respectively, with the RefSeq:13404 reference database. We primarily compare the runtime and memory usage for index construction and read alignment during queries, as these processes dominate the main computational resources. When dealing with short reads, PanTax initially requires index construction for the pangenome graphs, whereas this indexing process is unnecessary for long reads. Notably, the indexing process accounts for the majority of the time compared to the read-to-graph alignment. However, for a given pangenome reference, indexing needs to be performed only once. Notably, here and in the following sections, the taxonomic profiling time for all tools refers to the total time taken to execute the tools, including the time required for database construction. PanTax exhibits longer database construction and profiling times and requires larger peak memory usage compared to other tools. However, PanTax (fast) achieves comparable performance in terms of time and superior performance in terms of memory usage, when compared to other tools. For example (Supplemental Figure S7), on the sim-low dataset, PanTax (fast) requires 10.2 CPU hours, making it faster than tools such as KMCP (19.3 hours), Kraken2 (567 hours), and Centrifuge (152.6 hours), and only slightly slower than Ganon

(7.5 hours) and Bracken (7.8 hours). Additionally, PanTax (fast) requires the lowest peak memory usage (17.9 GB), whereas the second-lowest memory tool, KMCP, requires 18.8 GB. In the case of the real PD human gut dataset, PanTax (fast) consumes 16.6 CPU hours, which is only slower than Ganon (7.2 hours) and Bracken (7.8 hours), but several times faster than other tools such as Kraken2, Centrifuge, and Centrifuger. Furthermore, it requires 15.2 GB of RAM, which is the lowest memory usage among all the tools.

Similar to short-read datasets, in long-read datasets (Supplemental Table S17), PanTax exhibits longer database construction and profiling times and requires higher peak memory usage compared to other tools. However, widely used tools such as Kraken2 require comparable time and memory usage. Despite this, PanTax (fast) delivers performance that is either superior or comparable to other tools. For example (Supplemental Figure S7), on sim-low (PacBio HiFi) data, PanTax (fast) requires 13.9 CPU hours, making it slightly slower than the fastest two tools (Ganon: 6.6 hours, MetaMaps: 10.4 hours), but MetaMaps consumes a substantial amount of memory (around 400 GB) whereas Ganon also requires a moderate amount of memory (67.8 GB). In contrast, PanTax (fast) requires the lowest memory usage (7.3 GB), with the second lowest being KMCP (19.5 GB). On the real Omnivorous human gut dataset (HiFi), PanTax (fast) requires 79.8 CPU hours, which is slower than Ganon (8.2 hours), KMCP (48.8 hours), Centrifuge (53 hours) and MetaMaps (37 hours), but shows 1.9× and 7.1× faster than Centrifuger and Kraken2, respectively. Regarding memory usage, PanTax (fast) requires comparable memory usage to KMCP (20.7 GB and 19.9 GB, respectively), both of which require 5 to 20 times less memory than the alternative methods. On the sim-high-gtdb datasets, which utilize a large-scale reference database (GTDB:206273), PanTax (fast) achieves comparable speed to MetaMaps, being only 1-3 times slower than Ganon, but significantly faster (approximately 4-50 times) than other tools such as KMCP, Kraken2, Bracken, Centrifuge, and Centrifuger (Supplemental Figure S7, Supplemental Table S18). Notably, PanTax (fast) requires the lowest peak memory usage (around 30 GB) across all sim-high-gtdb datasets, while other tools require at least 200 to 1200 GB (Supplemental Figure S7, Supplemental Table S18).

It is worth noting that for profiling a single species, although PanTax requires more time and memory than other tools like StrainScan, StrainGE, and StrainEst, the time (approximately 27 CPU hours) and memory (around 20 GB) required are still acceptable (Supplemental Table S19).

References

Simon HY, Siddle KJ, Park DJ, and Sabeti PC. 2019. Benchmarking metagenomics tools for taxonomic classification. *Cell.* **178**: 779–794.
