# Supplementary material for: Strain-level metagenomic profiling using pangenome graphs with PanTax
Source: Genome Res. 2026 Feb;36(2):405–20. doi: 10.1101/gr.280858.125 (PMC12863173; doi:10.1101/gr.280858.125)
Supplement: Supplement 2 [file Supplemental_Methods.pdf]

Strain-level metagenomic profiling using pangenome graphs with PanTax

Wenhai Zhang[1,2,†], Yuansheng Liu[3,†], Guangyi Li[2,†], Jialu Xu[2], Enlian Chen[2], Alexander Schönhuth[4,*] Xiao Luo[1,2,*]

[1] Hunan Research Center of the Basic Discipline for Cell Signaling, Hunan University, Changsha, China

[2] College of Biology, Hunan University, Changsha, China

[3] College of Computer Science and Electronic Engineering, Hunan University, Changsha, China

[4] Faculty of Technology, Bielefeld University, Bielefeld, Germany

[†]These authors contributed equally to the work.

[*]To whom correspondence should be addressed.

Email: aschoen@cebitec.uni-bielefeld.de

Email: xluo@hnu.edu.cn

Supplemental Methods

S1 Datasets

S1.1 Datasets for multi species strain-level taxonomic profiling evaluation tasks

Simulated datasets (sim-low, sim-high). We made use of CAMISIM (Fritz et al., 2019) to generate 10 metagenomic datasets (2 × 5) with different complexities (i.e. low and high) and different sequencing read types (i.e. Illumina, PacBio HiFi/CLR and ONT R10.4/R9.4.1 reads), which reflect most of application scenarios in current metagenomic research. Here, we applied PBSIM2 (Ono et al., 2021), as a most recent tool to simulate PacBio HiFi and ONT reads instead of the default simulator (PBSIM) in CAMISIM using sampling-based simulation mode. All strains in the datasets follow a log-normal distribution. The PacBio HiFi sample FASTQ file was down-sampled from an anaerobic digester sludge sequencing sample (SRA accession: ERR7015089) (Feng et al., 2022). The ONT R10.4 and R9.4.1 template files are derived from a recent ONT long-read metagenomic study (Sereika et al., 2022). The low complexity dataset (named "sim-low") comprises 60 strains stemming from 30 species (2 strains per species), while the high complexity (named "sim-high") dataset comprises 1000 strains from 373 species (so a little less than 3 strains per species on average). For all datasets, the genomes used were retrieved from RefSeq (O'Leary et al., 2016)(see Supplementary Data for the details).

For the sim-low dataset, the relative abundances of strains vary from 0.30% to 6.43% and corresponding sequencing coverage over different strains varies from 0.96× to 20.3×. The mean sequencing coverage of strains is about 5.3×. For the sim-high dataset, relative abundances of strains vary from 0.04% to 0.30%. The corresponding sequencing coverage over different strains vary from 1.0× to 8.8×, and the mean sequencing coverage of strains is about 2.9×. Notably, in real-world scenarios, some novel strains may be present in a sample, meaning that not all strains can be identified using the reference database. To simulate this situation, we have deliberately included some strains in our simulated samples that are not present in our pangenome reference databases. Specifically, for the sim-low dataset, 56 out of 60 strains are found in the pangenome reference databases, while the remaining 4 strains are not. Similarly, for the sim-high dataset, 795 out of 1000 strains are present in the pangenome reference databases, whereas the other 205 strains are absent.

Simulated datasets with introduced mutations (sim-low-mut1, sim-high-mut1, sim-low-mut2, sim-high-mut2). In general, most microbial communities do not contain strains that exactly match reference genomes, as the dominant strains within these communities are often unique to each specific community. Therefore, to reflect this variability, we generated datasets in which the genomes differ from the reference genomes. Building on the genomes used in the previously simulated datasets, we introduced random mutations (including indels and single nucleotide substitutions) into all strains at mutation rates of 0.1% and 1%, thereby creating mutant strains. Using the same read simulation approach, new datasets were generated. Specifically, mutations at 0.1% and 1% were introduced into strains from the sim-low dataset to produce the sim-low-mut1 and sim-low-mut2 datasets, respectively. Similarly, the sim-high dataset was used to generate sim-high-mut1 and sim-high-mut2. In total, considering five types of sequencing data, we generated 20 new metagenomic datasets ($2 \times 2 \times 5$) with random mutations.

Simulated datasets for GTDB (sim-high-gtdb). To assess the strain profiling capabilities of PanTax using a large-scale reference database, we generated a high-complexity simulated dataset (sim-high-gtdb) using CAMISIM. This dataset incorporates multiple sequencing technologies, including Illumina, PacBio HiFi/CLR, and ONT R10.4/R9.4.1. Unlike previous simulated datasets that modeled complex microbial communities, the sim-high-gtdb dataset was specifically designed to evaluate PanTax's performance with a comprehensive reference database. Strain genomes were exclusively sourced from the GTDB:206273 database. Species containing more than two strains were considered, from which 1,000 strains were randomly selected. The sequencing coverage depth of these strains ranged from 1.1× to 9.0×.

Real datasets (Zymo1 and Zymo2, PD human gut, Omnivorous human gut, Healthy human gut).

Zymo1 and Zymo2. The ZymoBIOMICS Microbial Community Standard dataset (catalog number: D6300), referred to as Zymo1. In Zymo1, all bacteria are equally

distributed at 12%, except for the two yeasts, which are each present at 2% (Nicholls et al., 2019). Sequencing data for Zymo1 include Oxford Nanopore Technologies (ONT) R10 reads (`https://github.com/LomanLab/mockcommunity`, 24 gigabytes FASTQ), ONT R9.4.1 reads (SRA accession: ERR3152364, 27 gigabytes FASTQ), and Illumina reads (SRA accession: ERR2984773, 6.2 gigabytes FASTQ). The ZymoBIOMICS Microbial Community Standard II (catalog number: D6310), hereafter referred to as Zymo2, contains the same microbial species as Zymo1 but with a logarithmic distribution of cell counts ranging from 89.1% to 0.000089% (Nicholls et al., 2019). This community includes ONT R9.4.1 reads (SRA accession: ERR3152366, 31 gigabytes FASTQ) and Illumina reads (SRA accession: ERR2935805, 24 gigabytes FASTQ). Following the protocol recommendations, bacterial relative abundances were calculated based on genome copy number and used as the ground truth, with the two yeasts excluded. Note that we used both ONT R10 and R9.4.1 data from Oxford Nanopore GridION sequencing platform.

PD human gut (Illumina). The Illumina sequencing data for individuals with Parkinson's disease (PD) (SRA accession: SRR19064874) were obtained from the study by (Wallen et al., 2022). In this investigation, DNA was extracted from fecal samples provided by participants and sequenced using the Illumina NovaSeq 6000 platform. Quality control (QC) of the sequencing data was performed using BBDuk and BBSplit, included in the BBMap package (`https://sourceforge.net/projects/bbmap/`).

Omnivorous human gut (PacBio HiFi). The PacBio HiFi metagenomic sequencing data (SRA accession: SRR17687125, 29.6 gigabytes in FASTQ format) from the fecal sample of a healthy Korean omnivorous individual were sourced from the study by (CY Kim et al., 2022), sequenced on the PacBio Sequel II platform. QC was conducted using fastp (version 0.24.0) (S Chen, 2023) and minimap2 (version 2.28-r1209) (Li, 2018). To optimize computational efficiency, 20% of the data were randomly subsampled for analysis using seqtk (version 1.3-r106, https://github.com/lh3/seqtk).

Healthy human gut (ONT). The ONT sequencing data (SRA accession: SRR18490940) from the fecal sample of a healthy individual, free of any observable disease or infection, were obtained from the study by (L Chen et al., 2022). As per the study's methodology, libraries were prepared using the ONT Ligation library preparation kit (SQK-LSK109, EXP-NBD104, and EXP-NBD114). Sequencing was carried out on the ONT PromethION platform utilizing FLO-PRO002 flow cells. QC was performed using NanoFilt (version 2.8.0) (De Coster et al., 2018) and minimap2.

Spiked-in datasets. Since accurately evaluating the performance of various methods on the real datasets mentioned above is challenging due to the absence of a known ground truth, we further generated spiked-in datasets to assess these methods in the context of real scenarios. Eight *Vibrio cholerae* strains were selected from a collection of 13,404 complete genomes (as described in Step 1 of the Methods section), and five metagenomic datasets with different sequencing read types (i.e., Illumina, PacBio HiFi/CLR, and ONT R10.4/R9.4.1) were generated using CAMISIM. The ANI between each pair of *Vibrio cholerae* strains ranges from 95.98% to 99.48%. The sequencing coverage of these eight strains varies from 2.4× to 6.0×. The sequencing reads of this eight-strain mixture

were then introduced into the corresponding real metagenomic data obtained through small-scale sampling for each sequencing read type. The SRA accessions for the real metagenomic data are as follows: Illumina data (SRR10917775) (Jin et al., 2022), PacBio HiFi data (SRR15275213) (CY Kim et al., 2022), PacBio CLR data (SRR10917793) (Jin et al., 2022), ONT R10.4 data (SRR27910580) (Campos-Madueno et al., 2024), and ONT R9.4 data (SRR18490952) (L Chen et al., 2022).

S1.2 Dataset for single species strain-level taxonomic profiling evaluation tasks

Simulated datasets: *S. epidermidis* strain mixtures (3 strains, 5 strains, 10 strains). From a total of 34,697 complete genomes in RefSeq (as detailed in Step 1 of the Methods section), we randomly selected 3, 5, and 10 strains of *Staphylococcus epidermidis* (species taxid: 1282) to simulate three Illumina metagenomic datasets (corresponding to 3, 5, and 10 strains) using CAMISIM. The ANI between each pair of strains across all three datasets ranges from 99.20% to 99.33%, 98.62% to 99.33%, and 96.96% to 99.47%, respectively. The sequencing coverage of the strains across all three datasets varies from 1× to 10×.

Real datasets: two cultured *S. epidermidis* strain mixtures. In the study by (Emiola et al., 2020), the authors cultured two *Staphylococcus epidermidis* strains (species taxid: 1282), NIHLM001 (RefSeq assembly accession: GCF_000276145.1) and NIHLM023 (RefSeq assembly accession: GCF_000276305.1), both isolated from human skin and sharing a 97% ANI. NIHLM001 displayed high resistance to the bacteriostatic antibiotic erythromycin, while NIHLM023 was susceptible. The two strains were mixed in equal proportions (1:1) and cultured under two conditions: one with erythromycin treatment (Ery) and the other without antibiotics (no_ATB). Samples were collected at three time points for metagenomic sequencing. For our analysis, we downloaded the Illumina data from the third biological replicate of each condition, resulting in a total of six sequencing datasets (SRA accessions: SRR10610656, SRR10610665, SRR10610664, SRR10610659, SRR10610658, SRR10610657).

S2 Select high-quality genomes for species

We retrieved high-quality, complete (in particular gap-free) bacterial genomes from NCBI's RefSeq (O'Leary et al., 2016) on July 18, 2023 (RefSeq Release 219). The NCBI's RefSeq (release 219) establishes a comprehensive reference database, consisting of 34,697 complete genomes. In view of plasmid sequences being typically considerably shorter than bacterial genomes, plasmid sequences were excluded from our experiments. We remind that one can put particular attention also to plasmids, if desirable, by augmenting our database with plasmid reference sequence based pangenomes, for example. For each species, we compute a pairwise distance matrix using FastANI (Jain et al., 2018), where rows and columns correspond to the strains of the species under consideration,

and single entries in the matrix indicate the ANI between two strains of that species. Subsequently, we apply a customized graph-based clustering approach (see Algorithm in Supplemental Methods S16) at ANI thresholds of 95% and 99.9%, ensuring that the mutual ANI values of the strain-specific genomes for each species fall within this range. To maintain a balance between sensitivity and computational cost, we determined no more than 10 representative genomes for each species as a reasonable choice. Following these guidelines, we included 8,778 species, amounting to a total of 13,404 strains, in the refined reference database (labeled as RefSeq:13404). Of the 8,778 species included, 1,313 comprised multiple strains (Supplemental Figure S8). In addition, We used genome_updater (https://github.com/pirovc/genome_updater) to download genomes from the GTDB. To account for resource limitations during the execution of taxonomic classification tools, we restricted the download to a maximum of 100 genomes per species, resulting in a total of 343,362 strains. The resulting subset of the GTDB genomes was used as our reference Genome Taxonomy Database. We adopted a similar strategy to select high-quality genomes from the GTDB (Release R220) and ultimately constructed a refined reference database comprising 206,273 strains (labeled as GTDB:206273).

S3 Construction of the large pangenome reference

We construct a variation graph (as the particular, and generally most popular type of pangenome graph) for each species, by using the (efficient and unbiased) pangenome graph builder PGGB (Garrison, Guarracino, et al., 2024). We construct the variation graph $G = (V, E, P)$ from the (multiple) strain genomes pertaining to a species, where V are the nodes, E are the edges and P are the paths in G corresponding to the input strain-specific genomes used for constructing the graph. G is a bidirected sequence graph, where each vertex $v \in V$ corresponds to a sequence $\text{seq}(v)$ that consists of single or multiple nucleotides (A, T, C, G).

Edges $e_{ij} = (v_i, v_j) \in E$ indicate that the sequences $\text{seq}(v_i)$ and $\text{seq}(v_j)$ represented by nodes v_i and v_j appear as a sequential subsequence in one of the genomes that contribute to the graph. Because paths P outline these genomes, paths P cover the graph, or, in other words, each edge is contained in one of the paths from P. Let v_i^* denote the reverse-complement of node v_i. Then the reverse edge e_{ij}^* of e_{ij} is defined as $e_{ij}^* = (v_j^*, v_i^*)$. Both nodes and edges can be traversed in either the forward or the reverse direction in the graph. In general, known strains can be identified as the specific paths $p = (v_1, ..., v_l) \in P$, as part of the definition of the species-specific pangenome graph G.

For species that involve only one strain, the task is to turn its corresponding sequence into a dedicated variation graph, acting as representative counterpart to the pangenome graphs constructed from multiple strains. Here, we achieve this by partitioning the genome sequence into fragments of (the relatively short) length 1024bp, each of which is to correspond to a node in the graph, which, by design, is chain-like.

Subsequently, one merges all species-specific pangenome graphs into a single, large pangenome graph, by relabeling nodes, while never introducing new nodes or edges. Of note, the species-specific pangenome graphs can be computed in paraellel, which avoids

computational bottlenecks in this step. As outlined above, this procedure supports the unified and unambiguous classification of single reads via *one* read-to-graph alignment, instead of having to align a read with each of the (here, by default: 8,778) species-specific pangenome graphs individually. In other words, instead of having to perform and evaluate 8,778 alignments in a statistically involved mutual context, the merging of graphs delivers one encompassing, optimal and statistically sensible alignment that directly indicates the species the read optimally aligns with.

PanTax adopts a two-step approach by first constructing species-specific pangenome graphs and subsequently merging them into a large reference graph, rather than directly building a unified graph from all genomes. This design choice is motivated by computational considerations, as current pangenome construction tools such as PGGB (Garrison, Guarracino, et al., 2024) require extremely high time and memory resources when scaling to large numbers of genomes. To improve practicality, our two-step approach substantially reduces computational burden while retaining biological resolution. This strategy is conceptually similar to the chromosome-partitioned construction described in the draft human pangenome reference (Liao et al., 2023).

S4 Index construction for short-read sequence-to-graph alignment

For short reads, we resort to building the graph Burrows-Wheeler transform (GBWT) index manually, via a 'fast indexing mode', which relies on three commands provided by vg (Garrison, Sirén, et al., 2018), which considerably speeds up index construction. Although the default fully automatic construction of the index via 'vg autoindex' is supposed to be more accurate, classification results did not differ in our experiments, which explains our choice.

S5 MAPQ-based false-positive species filtering

We examine the MAPQ (mapping quality) scores of all the reads whose optimal alignments correspond to the particular species. We raise two key criteria: first, we require at least one read to reach a MAPQ of 60, which indicates a statistically significant unique match of at least one read with that species. We further require that at least one tenth of the reads that optimally align with a species exhibit a MAPQ of at least 2; if less than one tenth of the reads aligned with a species have MAPQ less than 2, the species is flagged as false positive.

S6 Path abundance optimization

Furthermore, we offer strain-level classification, reflecting the main purpose of our approach. Having already constructed a species-specific pangenome graph by collapsing identical sequences from multiple strains (as described in Step 2), we formulate a path

abundance optimization problem for each species-specific pangenome graph, following an approach suggested in (Baaijens et al., 2020). In this step, the abundance we refer to is absolute abundance, which is the average coverage depth. This step aims to resolve strain-level taxonomic classification. Let V be the set of all nodes in a species-specific pangenome graph, P be the set of paths corresponding to all strains used to construct the pangenome graph for a particular species (where a strain's genome sequence corresponds to a path in the pangenome graph in general), and $\widehat{a_v}$ be the absolute abundance of node $v \in V$ in the graph, as estimated by evaluating the reads whose alignments include v (i.e., the average coverage depth of a single base in this node), where we follow the definitions provided in (Baaijens et al., 2020) in a one-to-one fashion. For a path $p \in P$ (again following (Baaijens et al., 2020)), we define $a_p \in \mathbb{R}_{\geq 0}$ to be the absolute abundance of path p, which corresponds to the abundance of the strain that corresponds to p. Here, we would like to predict all a_p by raising them as variables in the program

$$\min \sum_{v \in V} \left| \widehat{a_v} - \sum_{p \ni v} a_p \right| \qquad \text{s.t. } a_p \geq 0 \quad \forall p \in P \tag{1}$$

which we refer to as *path abundance optimization (PAO)* problem. Values $a_p \geq 0$ establishing a solution are output as the abundance values of the strains that correspond to the paths p.

As for an explanation, the objective function of the PAO problem aims to estimate the abundance (i.e. the normalized read coverage) of each strain by minimizing the difference between the abundances of the nodes one has observed (derived from the sequence-to-graph alignments in Step 3) and the abundances of the nodes that correspond to the abundances of the strains whose genomes include the nodes. The PAO problem is convex in nature, and can be efficiently linearized and solved using a linear programming solver of choice. Here we employed the commercial solver Gurobi (Gurobi Optimization, 2023), but alternative open-source solvers such as HiGHS (Huangfu and Hall, 2018), Cbc (Lougee-Heimer, 2003), and GLPK (Makhorin, 2008) are also compatible (Supplemental Methods S7 and Supplemental Table S20). So, from a theoretical perspective, this problem is polynomial-time solvable. In addition, since the size of the candidate set of strain paths P is typically not overly large (usually ≤ 10), the optimization problems can be solved in parallel across the species, which renders this step computationally very efficient.

Note that for each species, we conduct two iterations of path abundance optimization. To further mitigate the odds of raising false positives during classification, we add a filtering operation prior to each iteration of PAO. To illustrate the filtering step, we first define a *triplet node* as a set of three consecutive nodes within a path. A triplet node that is exclusive to a single strain, that is the three nodes only appear in that order in one of the paths $p \in P$, is referred to as *strain-specific triplet node.*

(1) For the filtering step that precedes the first iteration of solving the PAO, let V_{reads} be the consecutive node triples covered by reads aligning with the species-specific pangenome graph and V_{strain} reflect the set of strain-specific triplet nodes that show in

the path corresponding to the strain, as just defined. We then raise the metric f_{strain}, as per the definition

$$f_{\text{strain}} := \frac{|V_{\text{reads}} \cap V_{\text{strain}}|}{|V_{\text{strain}}|} \tag{2}$$

As per its definition, f_{strain} captures the proportion of strain-specific triplet nodes in the strain genome (reference) the concatenation of the three sequences of which appears in the reads of the sequencing sample. As a ratio, the value of f_{strain} naturally ranges from 0 to 1, with 1 indicating that all strain-specific triplet nodes are covered by reads from the sample. In strain-level profiling for single species, read alignment is not affected by interspecies homologous regions, thereby achieving higher accuracy. Consequently, the f_{strain} threshold for each strain is set more stringently and dynamically adjusted based on the default threshold.

Let $\mathbf{v}_{\text{triplet}}$ refer to strain-specific triplet nodes, as defined above, and let $c(\mathbf{v}_{\text{triplet}})$ be the average read coverage of $\mathbf{v}_{\text{triplet}}$, where averaging is across nucleotides in the sequence of $\mathbf{v}_{\text{triplet}}$. Further, we introduce $a_{p,\text{triplet}}$, which, unlike a_p that resulted from the solution of the PAO, estimates the abundance of the strain that corresponds to p by taking the average of the coverages of the triplet nodes, that is the average of the $c(\mathbf{v}_{\text{triplet}})$ in p:

$$a_{p,\text{triplet}} := \frac{\sum_{\mathbf{v}_{\text{triplet}} \in V_{\text{strain}} > 0} c(\mathbf{v}_{\text{triplet}})}{|V_{\text{strain}}|} \tag{3}$$

Subsequently, we solve the PAO problem a first time for only the strains that were not discarded in the filtering procedure (that is whose f_{strain} was found to be ≥ 0.3 for short reads or ≥ 0.5 for long reads), and record the strains, respectively the paths p in the species-specific pangenome graph to which they correspond whose $a_p > 0$, that is we keep all strains that make part of the solution of the PAO.

(2) For the filtering step that precedes the second iteration of solving the PAO, we raise the metric d_{strain}

$$d_{\text{strain}} := \frac{|a_p - a_{p,\text{triplet}}|}{a_p + a_{p,\text{triplet}}} \tag{4}$$

As for a brief explanation, d_{strain} quantifies the divergence between the strain abundance a_p predicted in the first iteration of the PAO and the strain abundance estimate based on the average coverage of all strain-specific triplet nodes.

Before proceeding with the second iteration of the PAO, we filter out further potentially false positive strains by keeping only strains where $d_{\text{strain}} \leq 0.46$.

At this stage, for strains with $0.46 < d_{\text{strain}} \leq 0.6$, we perform a rescue operation for high-confidence strains among them. We define the base coverage of a strain as b_{strain}, and introduce a rescue parameter $r_{\text{strain}} = b_{\text{strain}} * f_{\text{strain}}$. A strain will be retained if it satisfies the condition $r_{\text{strain}} \geq 0.85$. Note that f_{strain}, d_{strain}, and r_{strain} are empirical parameters. The sensitivity analysis of these parameters is provided in Supplemental Methods S8, with results shown in Supplemental Figures S9–S11.

Finally, we solve the PAO a second time. The final output of strains are the ones whose a_p is greater than zero in that second iteration. For the rescued strains, the minimum value between the PAO final solution a_p and $a_{p,\text{triplet}}$ is selected as their absolute abundance.

S7 Comparative performance of gurobi and open-source linear programming solvers

To evaluate the performance of the open-source solvers HiGHS, Cbc, and GLPK alongside Gurobi, we tested them on the sim-low illumina dataset. The results (Supplemental Table S20) indicate that all solvers produce estimates consistent with the ground truth across multiple metrics. While the open-source alternatives provide stable solutions, they generally require longer computational times.

S8 Sensitivity analysis of key parameters in strain level profiling

Strain profiling in PanTax involves three parameters: f_{strain}, d_{strain}, and r_{strain}, among which f_{strain} and d_{strain} are the most important. Their definitions and calculations are provided in the Methods section. We conducted a comprehensive sensitivity analysis of these parameters: 1) With the cutoff $r_{\text{strain}} \geq 0.85$ fixed, we varied f_{strain} from 0 to 0.9 (in steps of 0.1; note that $f_{\text{strain}} = 1$ filters out all strains) and d_{strain} from 0 to 1 (also in steps of 0.1). Experiments were conducted on six datasets: sim-low Illumina, sim-low PacBio HiFi, sim-high Illumina, sim-high PacBio HiFi, Zymo1 Illumina, and Zymo1 ONT R10. We also tested other types of long reads and observed similar patterns across metrics; hence, we used PacBio HiFi as the representative for long reads, and ONT R10 for Zymo1, which lacks PacBio HiFi data. We evaluated performance primarily in terms of precision, recall, F1 score and BC distance. 2) Based on the results of experiment 1, we selected robust parameter values for f_{strain} and d_{strain} (short reads: $f_{\text{strain}} \geq 0.3$; long reads: $f_{\text{strain}} \geq 0.5$; $d_{\text{strain}} \geq 0.46$ for both). We then varied the r_{strain} cutoff from 0.05 to 0.95 in increments of 0.1, and evaluated performance in terms of F1 score and BC distance. 3) To investigate the effect of parameters on low coverage strains ($\leq 3\times$), we repeated the experiments under the same setting as 1), but focused on recall, using four datasets (sim-low Illumina, sim-low PacBio HiFi, sim-high Illumina, sim-high PacBio HiFi). These analyses help clarify the robustness of parameters.

S9 Strain-level taxonomic profiling

We firstly define a metric d_{species} for filtering, which quantifies the divergence between the absolute abundance c_i of a species i (as described in Step 5) and the total absolute

abundance of all its strains $c_{i,strains}$ as determined by the PAO solution:

$$d_{\text{species}} \quad := \quad \frac{2 * |c_i - c_{i,strains}|}{c_i + c_{i,strains}} \tag{5}$$

We filter out further potentially false positive species and its strains with $d_{\text{species}} > 0.2$. The divergence is typically caused by reads originating from one species being aligned to another non-existent species. These reads often come from shared regions, leading to their excessive accumulation in these areas. Our objective is to filter out these non-existent species.

There is one caveat: the sum of the absolute abundances of the strains could exceed the absolute abundance of the most abundant strain within the species. In that case, we scale the absolute abundances of the strains such that their sum equals that of their species.

The final, additional step is to provide relative abundance estimates for any strain predicted to make part of the sample. For that, one determines the absolute abundances of the strains and divides them by the sum of the absolute abundances of all strains in the sample.

S10 Assembly-based profiling

First, each assembler was executed using recommended settings. For myloasm, which could not process degenerate bases in the simulated data, all degenerate bases were replaced with 'A' to ensure successful assembly; all other tools used the original simulated datasets. The resulting MAGs were then aligned to the reference genome database using minimap2 (version 2.28-r1209) (Li, 2018) with the parameter "-x asm5".

For each contig, the best alignment was selected based on a combination of identity, mapping quality, and approximate divergence metrics. Each contig was uniquely assigned to a single reference genome. All reference genomes with at least one mapped contig were considered predicted strains.

To estimate strain abundance, we aggregated all contigs assigned to each predicted strain. For each contig, we calculated the product of its length and coverage depth, summing these values to obtain the total number of mapped bases per strain. This total was then divided by the reference genome length to derive the average coverage depth. Finally, relative abundances were calculated by normalizing the average coverage depths across all predicted strains.

S11 Reference diversity

To explore the effect of increasing reference diversity on PanTax performance, a series of reference databases were constructed with progressively increasing numbers of species and strains. We performed a simulated benchmark using the genus *Streptococcus*, which includes 1,098 complete genomes in RefSeq, spanning 83 species. After applying graph-based clustering for redundancy removal, we retained 596 representative strains. From

these, a set of 60 bacterial strains was simulated with CAMISIM using different sequencing technologies (Illumina, PacBio HiFi, PacBio CLR, ONT R10.4, and ONT R9.4.1) to generate the SimRef datasets, with strain relative abundances identical to those in the sim-low dataset. We then constructed five reference sets of increasing diversity for SimRef: 1) SimRef1: The 60 strains used for simulation (perfect reference). 2) SimRef2: SimRef1 plus 10 additional strains per species (or all strains if < 10). 3) SimRef3: SimRef1 plus 20 additional strains per species. 4) SimRef4: SimRef1 plus strains from other *Streptococcus* species (same genus). 5) SimRef5: All 596 strains (full genus-level reference). Similarly, we conducted the same set of experiments on the Zymo1. The Zymo reference database, constructed as described in the "Strain-level taxonomic profiling for multiple species, Real dataset" section, is referred to as ZymoRef3. For species with more than 10 genomes, we selected one-third and two-thirds of the strains to construct ZymoRef1 and ZymoRef2, respectively.

S12 Benchmarking the impact of graph complexity and long read sequencing technologies on alignment accuracy

We extended the reference diversity experiment using the SimRef datasets. As the number of strains in the reference database increases, the graph complexity grows correspondingly. The number of graph nodes was employed to represent graph complexity. We then quantified the distribution of read mapping qualities across datasets with varying graph complexities and sequencing technologies.

S13 Benchmarking PanTax under strain quantity and genome similarity scaling

To explore the scaling limits of PanTax, we conducted benchmark tests in two directions based on the built reference database: 1) strain quantity scaling. From the RefSeq:13404 database (restricted to species with more than two strains), we randomly selected 1,000, 2,000, 3,000, and 4,000 strains to simulate four datasets: sim-high1000, sim-high2000, sim-high3000, and sim-high4000. Illumina and PacBio HiFi datasets were simulated using CAMISIM, with strain abundances following a log-normal distribution. Notably, the sim-high1000 dataset differs from sim-high in that all strains in sim-high1000 were drawn exclusively from the RefSeq:13404 database. 2) genome similarity scaling. From the 70 non-redundant genomes of *Staphylococcus epidermidis* (species taxid: 1282), we randomly selected 1, 2, 5, 10, 20, 30, 40, and 50 strains. ANI was calculated to quantify genomic similarity, which increased with strain number (Supplemental Figure S6B). Data simulation followed the same protocol as in genome quantity scaling.

S14 Metrics for evaluation

We evaluate the performance of taxonomic classifiers using metrics that are widely adopted in various relevant studies (Simon et al., 2019; K Zhu et al., 2022; Luo et al., 2022). The primary criteria for assessing metagenomic classification methods are precision and recall. At the level of taxa (strain level that we focus on), precision measures the fraction of correctly identified taxa among all taxa that were identified. Recall, at the level of taxa, evaluates the proportion of correctly identified taxa compared to all taxa present in the sample.

The F1 score, the harmonic mean of precision and recall, is frequently employed as a balanced metric for evaluating classifiers.

However, precision, recall, and the F1 score may not reflect the performance of classifiers in metagenomic samples sufficiently comprehensively, because the F1 score neglects the correct assessment of low-abundance taxa, whose accurate detection, however, can be crucial. A approach to evaluating the contents of metagenomes that takes low-abundance taxa into sufficiently accurate account is to evaluate the metagenomes in terms of curves that plot precision and recall across varying abundance thresholds. Calculating the area under this curve ("AUPR") yields a metric that does not neglect the performance of a classifier also with respect to taxa of lower abundance (Simon et al., 2019). For the calculation of AUPR, refer to (Simon et al., 2019). Classifiers that fail to recall all ground-truth taxa are penalized with a zero AUPR score from their greatest recall achieved up to 100% recall. Conversely, classifiers that achieve 100% recall are not further penalized in their AUPR score for additional false positive taxon calls. Please see "AUPR calculation example" in the Supplemental Methods S15 for illustrative examples.

Beyond the accurate identification of taxa only, it can be imperative to provide accurate estimates of the abundances of the taxa that make part of a metagenome. An example for the necessity of such practice are metagenome-wide association studies ("MetaGWAS") where fluctuations in the proportions of the participating taxa can have profound implications in terms of the phenotypes supported by the metagenome.

The relative abundances of taxa across various samples are comparable, making them a primary focus in MetaGWAS studies.

The ultimate output of taxonomic profiling at the strain level (Steps 7) is precisely these relative abundances. Notably, in this study, we propose a set of metrics to assess the accuracy and reliability of these relative abundance estimates.

To determine abundance profiles for tools that can output read assignment, we follow the exact same protocol employed for PanTax.

Namely, all tools use relative taxonomic abundance for evaluation, where the genome length of a strain is used to normalize read coverage. If methods offer taxonomic abundance profiling as output directly, we make use of that.

The L1 distance, L2 distance and BC distance, commonly used metrics, serve as a quantitative evaluation of abundance profiles (Sun et al., 2021; Simon et al., 2019). Given a sample, let I represent the intersection set of estimated and true taxa. For a taxon $i \in I$, let $\widehat{a_i}$ and a_i represent the estimated and true abundance of taxon i, respectively.

Then, the L1 distance is computed as follows: $\mathrm{L1} := \sum_{\mathrm{i}\in\mathrm{I}} |\mathrm{a_i} - \widehat{\mathrm{a_i}}|$. The L2 distance is computed as follows: $\mathrm{L2} := \sqrt{\sum_{\mathrm{i}\in\mathrm{I}}(\mathrm{a_i} - \widehat{\mathrm{a_i}})^2}$. The calculation of the BC distance is performed using the *braycurtis* function from the *SciPy* Python package, and it is defined as $\mathrm{BC} := \frac{\sum_{\mathrm{i}\in\mathrm{I}}(\mathrm{a_i}-\widehat{\mathrm{a_i}})}{\sum_{\mathrm{i}\in\mathrm{I}}(\mathrm{a_i}+\widehat{\mathrm{a_i}})}$. A smaller distance indicates a higher similarity between the estimated and true abundances, while greater distance suggests greater divergence. However, it is worth noting that abundance profile distance is particularly sensitive to the accurate quantification of highly abundant taxa within a sample, as these taxa exert a significant influence on the overall similarity between abundance profiles. To address this, we introduce two complementary metrics: the absolute frequency error (AFE) and the relative frequency error (RFE). AFE is defined as: $\mathrm{AFE} := \frac{1}{|I|}\sum_{i\in I} |a_i - \widehat{a_i}|$, whereas RFE is defined as: $\mathrm{RFE} := \frac{1}{|I|}\sum_{i\in I} |a_i - \widehat{a_i}|/a_i$. These two metrics have been adapted from previous studies on viral haplotype abundance evaluation (Luo et al., 2022; Baaijens et al., 2020). The AFE and RFE metrics provide insights into the deviation of estimated abundances from their true values. Notably, the RFE metric balances the influence of low-abundance species or strains, because it scales divergences relative to the true abundance a_i of the taxa.

S15 AUPR calculation example

We present a straightforward example to elucidate the methodology for calculating AUPR (Area Under the Precision-Recall Curve). Notably, we refrain from utilizing the sklearn Python package for AUPR computation due to its reliance on a Boolean vector that merely indicates the correctness of taxa classification. This approach may lead to biased comparisons as it disregards the ground truth taxa present in the dataset when calculating both precision and recall.

Consider a predicted Boolean vector [1, 0, 1, 0], where '1' signifies a true positive (TP) taxon and '0' denotes a false positive (FP) taxon. The corresponding relative abundance vector, sorted in descending order, is [0.5, 0.3, 0.15, 0.05], and the total number of ground truth strains (TP+FN) is 3.

To calculate precision and recall, we consider taxa above various cutoffs. At a cutoff of 0.05, the updated predicted vector and relative abundance vector are [1, 0, 1, 0] and [0.5, 0.3, 0.15, 0.05], respectively. This yields a precision of 0.5 (2/4) and a recall of 0.67 (2/3), resulting in the first point on the diagram (0.67, 0.5).

Increasing the cutoff to 0.15 updates the vectors to [1, 0, 1] and [0.5, 0.3, 0.15], respectively, leading to a precision and recall of 0.67 (2/3), marking the second point (0.67, 0.67).

At a cutoff of 0.3, the vectors become [1, 0] and [0.5, 0.3], resulting in a precision of 0.5 (1/2) and a recall of 0.33 (1/3), placing the third point at (0.33, 0.5).

Finally, at a cutoff of 0.5, the vectors are [1] and [0.5], yielding a precision of 1 (1/1) and a recall of 0.33 (1/3), marking the fourth point (0.33, 1).

To ensure a smooth diagram, we initiate the plot at (0, 1), where 1 represents the precision of the fourth point. Since the recall of the first point is less than 1, we penalize

the AUPR between the greatest recall and 100% recall by setting the endpoint to (0.67, 0), where 0.67 is the recall of the first point. The precision and recall vectors are [1, 1, 0.5, 0.67, 0.5, 0] and [0, 0.33, 0.33, 0.67, 0.67, 0.67], respectively. We utilize the trapz function from the NumPy Python package (version 1.26.3) to approximate the AUPR by applying the trapezoidal rule.

S16 Algorithm

Algorithm 1 graph-based clustering

Input: The genome set $G = \{G_1, G_2, ..., G_N\}$ with N genomes from M species $S = \{S_1, S_2, ..., S_M\}$
Output: The non-redundant genome set $R = \{R[S_1], R[S_2], ..., R[S_M]\}$ from M species

for $S_i \in S$ do
  $L[S_i] = ComputeGenomeN50(S_i)$
  $B[S_i] = GetRepresentaiveGenome(S_i)$ // If the species has a reference genome or representative genome in NCBI RefSeq
for S_1 to S_M do
  $C_i = SelectGenomes(S_i, number)$ // default number 100
  for pair strains x, y in C_i do
    $A[x][y] = ComputeANI(x, y)$
// main
for $S_i \in S$ do
  if S_i has only one strain h then
    $R[S_i] = h$
  else
    for pair strains $x, y \in C_i$ do
      $E = [A[x][y] >= 99.9]$
    $Node = C_i$
    $Edge = E$
    $Graph = Graph(Node, Edge)$
    $H = ConnectedComponents(G)$
    for $H_j \in H$ do
      for $h \in H_j$ do
        if $L(h)$ is max then
          add h_i to O
    for pair strains $x, y \in O$ do
      $D = [A[x][y] >= 95]$
    $Node = O$
    $Edge = D$
    $Graph = Graph(Node, Edge)$
    $K = FindCliques(G)$
    $K = Sort(K)$
    $R[S_i] = K_1$ // K_1 has the maximum number of strains
    for $K_j \in K$ do
      for strain $x \in K_j$ do
        if $x == B[S_i]$ then
        $R[S_i] = K_j$
    if S_i all $A[x][y] < 95$ then
      if $B[S_i] \in C_i$ then
        $R[S_i] = B[S_i]$
      else
        for $h \in C_i$ do
          if $L(h)$ is max then
            $R[S_i] = h$
$R[S_i] = SelectGenomes(R[S_i], number)$ // default number 10

S17 Commands and versions of tools used for comparison

- PanTax v2.0.0
  Database construction
  default mode: `pantax -f $genomes_info -db $pantax_db --create -t 64`
  fast mode for short read: `pantax -f $genomes_info -s -p -r $read.fq --create --fast 1 -t 64`
  fast mode for long read: `pantax -f $genomes_info -l -r $read.fq --create --fast -t 64`

  Index construction for short read
  ```
  pantax -f $genomes_info -db $pantax_db --index -t 64
  ```

  Query
  (1) Short read
  ```
  pantax -f $genomes_info -s -p -r $read.fq --species-level --strain-level -db $pantax_db -t 64
  ```
  (2) Long read
  ```
  pantax -f $genomes_info -l -r $read.fq --species-level --strain-level -db $pantax_db -t 64
  ```
  The tool */usr/bin/time* is used to record the runtime and memory usage during the database construction, index construction and query.

- Kraken2 v2.1.3
  Database construction
  (1) Download taxonomy files
  ```
  kraken2-build --download-taxonomy --db $Kraken2_DB --threads 32
  ```
  We added custom strain-level taxids to the nodes.dmp and names.dmp files in the taxonomy file, enabling classification at the strain level.
  (2) Prepare all genomes in Kraken2 format and repeat this step to add all genomes to library.
  ```
  kraken2-build --add-to-library $genome --db $Kraken2_DB
  ```
  (3) Build index with 64 threads , with default parameters.
  ```
  kraken2-build --build --db $Kraken2_DB --threads 64
  ```
  The database construction for Kraken2 includes steps (2) and (3). The tool */usr/bin/time* is used to record the runtime and memory usage during the database construction process.

  Query
  (1) Short read
  ```
  kraken2 --db $Kraken2_DB --output kraken2_query_reads --report kraken2_query_report
   --threads 64 --paired $read1.fq $read2.fq
  ```
  (2) Long read
  ```
  kraken2 --db $Kraken2_DB --output kraken2_query_reads --report kraken2_query_report
   --threads 64 $read.fq
  ```
  Since the kraken_query_report file provides sequence abundance, we use the kraken_query_reads file which provides the assignment of each read, to estimate taxonomic abundance for evaluation. All other parameters are default. The kraken2_query_report is used as input for Bracken. The tool */usr/bin/time* is also used to record the runtime and memory usage during the query.

- Bracken v2.9
  Database construction
  ```
  bracken-build -d $Kraken2DB -t 64 -l <125/150/151>
  ```
  The tool */usr/bin/time* is used to record the runtime and memory usage during the database construction process. In addition, Bracken database construction is based on Kraken2 database. It requires read length(-l) for its index construction, so it is not suitable for long read.

Query

```
bracken -d $KrakenDB -i kraken2_query_report -o bracken_query -r <125/150/151>
```

Bracken is generally used for short read because it needs to specify the read length. Unlike other tools, Bracken does not take a fastq file as input, but post-processes based on the results of kraken2 report file(kraken2_query_report). We estimate the taxonomic abundance by parsing bracken_query. All other parameters are default. The tool */usr/bin/time* is also used to record the runtime and memory usage during the query.

- Centrifuge v1.0.4

  Database construction

```
centrifuge-build -p 64 --conversion-table seqid2taxid.map --taxonomy-tree nodes.dmp
--name-table names.dmp reference_genomes.fna $centrifugeDB
```

The seqid2taxid.map is a two columns, tab-separated file, which maps each contig to taxonomic ID(mapping sequence IDs to taxonomic IDs). The nodes.dmp and names.dmp files are the raw files obtained from the first step of Kraken2 (i.e., without the addition of custom strain-level taxids). We attempted to construct the database using the modified nodes.dmp and names.dmp files, which included custom strain-level taxids. However, after a week of execution without success, we decided to abandon the use of custom nodes.dmp and names.dmp files. The reference_genomes.fna is a fasta file that concatenates all input genomes. The seqid2taxid.map and reference_genomes.fna need to be created and provided to centrifuge-build. The tool */usr/bin/time* is used to record the runtime and memory usage during the database construction process.

Query

(1) Short read

```
centrifuge -k 1 -x $centrifugeDB -1 $read1.fq -2 $read2.fq -S centrifuge_query_reads
--report-file centrifuge_query_report --threads 64
```

(2) Long read

```
centrifuge -k 1 -x $centrifugeDB -U $read.fq -S centrifuge_query_reads
--report-file centrifuge_query_report --threads 64
```

Since the lowest taxid in the nodes.dmp file is at the species level, the report file cannot give the strain level abundance. So we use the centrifuge_query_reads file which provides the assignment of each read, to estimate taxonomic abundance for evaluation. All other parameters are default.

- Centrifuger v1.0.6-r175

  Database construction

```
centrifuger-build -t 64 --conversion-table seqid2taxid.map --taxonomy-tree nodes.dmp
--name-table names.dmp -l input_genomes.txt -o $centrifugerDB
```

The nodes.dmp and names.dmp files are the modified files obtained from the first step of Kraken2. The input_genomes.txt file contains the paths to all reference genomes, with one path per line, and needs to be manually created for input into centrifuger-build. The seqid2taxid.map is a two-column, tab-separated output file that maps sequence IDs to taxonomic IDs, and does not need to be provided.The tool */usr/bin/time* is used to record the runtime and memory usage during the database construction process.

Query

(1) Short read

```
centrifuger -k 1 -x $centrifugerDB -1 $read1.fq -2 $read2.fq -t 64 > cls.tsv
centrifuger-quant -x $centrifugerDB -c cls.tsv > centrifuger_report.tsv
```

(2) Long read

```
centrifuger -k 1 -x $centrifugerDB -u $read.fq -t 64 > cls.tsv
centrifuger-quant -x $centrifugerDB -c cls.tsv > centrifuger_report.tsv
```

Taxonomic abundance(column abundance) in centrifuger_report.tsv is directly used for evaluation. All other parameters are default.

- KMCP v0.9.4

  Database construction

  (1) `kmcp compute -i input_genomes.txt --kmer 21 --split-number 10 --split-overlap 150 --ref-name-regexp "^(.+)_genomic\.fna(?:\.gz)?$" --out-dir $kmcpDB/kmcp_refs_k21 -j 64`

  (2) `kmcp index --in-dir $kmcpDB/kmcp_refs_k21/ --num-hash 1 --false-positive-rate 0.3 --out-dir $kmcpDB/kmcp_refs_k21.kmcp -j 64`

  The input_genomes.txt file contains the paths to all reference genomes, with one path per line, and needs to be manually created for input into KMCP. The database construction for Kraken2 includes steps (1) and (2). The tool */usr/bin/time* is used to record the runtime and memory usage during the database construction process.

  Query

  (1) Short read

  Search: `kmcp search --db-dir $kmcpDB -1 $read1.fq -2 $read2.fq --out-file result.kmcp.gz --log result.kmcp.gz.log -j 64`

  Profile: `kmcp profile --taxid-map $taxid.map --taxdump $strain_taxonomy result.kmcp.gz --out-file result.kmcp.profile --metaphlan-report result.metaphlan.profile --sample-id 0 --cami-report result.cami.profile --binning-result result.binning.gz --log result.kmcp.profile.log --level strain`

  (2) Long read

  Search: `kmcp search --db-dir $kmcpDB $read.fq --out-file result.kmcp.gz --log result.kmcp.gz.log -j 64`

  Profile: `kmcp profile --taxid-map $taxid.map --taxdump $strain_taxonomy result.kmcp.gz --out-file result.kmcp.profile --metaphlan-report result.metaphlan.profile --sample-id 0 --cami-report result.cami.profile --binning-result result.binning.gz --log result.kmcp.profile.log --level strain`

  The strain_taxonomy is a directory that contains the modified files nodes.dmp and names.dmp obtained from the first step of Kraken2. The taxid.map is a two-column, tab-separated output file that maps to taxonomic ID to genome file name, and need to be provided. Taxonomic abundance (column percentage) in result.kmcp.profile is directly used for evaluation. All other parameters are default. The tool */usr/bin/time* is also used to record the runtime and memory usage during the query.

- Ganon v2.1.0

  Database construction

  `ganon build-custom --input-file input_genomes.txt --taxonomy-files nodes.dmp names.dmp --db-prefix $ganonDB --level strain -t 64`

  The input_genomes.txt file contains the paths to all reference genomes, with one path per line, and needs to be manually created for input into Ganon. The nodes.dmp and names.dmp files are the modified files obtained from the first step of Kraken2. The tool */usr/bin/time* is used to record the runtime and memory usage during the database construction process.

  Query

  (1) Short read

  `ganon classify --db-prefix $ganonDB --paired-reads $read1.fq $read2.fq --output-prefix results --report-type abundance -t 64`

  `ganon report -i results.rep --db-prefix $ganonDB --output-prefix tax_profile`

```
--report-type abundance -r all
```

(2) Long read

```
ganon classify --db-prefix $ganonDB -s $read.fq --output-prefix results --report-type
abundance -t 64
ganon report -i results.rep --db-prefix $ganonDB --output-prefix tax_profile
--report-type abundance -r all
```

Taxonomic abundance in tax_profile.re is directly used for evaluation. All other parameters are default. The tool */usr/bin/time* is also used to record the runtime and memory usage during the query.

- MetaMaps v0.1

  Database construction

```
perl combineAndAnnotateReferences.pl --inputFileList taxid2seq.txt
--outputFile reference_genomes.fa --taxonomyInDirectory taxonomy
--taxonomyOutDirectory taxonomy_out
perl buildDB.pl --DB $metamapsDB --FASTAs reference_genomes.fa --taxonomy taxonomy_out
```

  The taxid2seq.txt file is a two columns, tab-separated file, which maps taxonomic ID to each genome(fasta file). The taxonomy downloaded from NCBI.

  Query

```
metamaps mapDirectly -r reference_genomes.fa -q $read.fq -t 64 -o classification_results
metamaps classify --mappings classification_results --DB $metamapsDB -t 64
```

  Since the classification_results.EM.WIMP file provides sequence abundance, we use the classification_results.EM which provides the assignment of each read, to estimate taxonomic abundance for evaluation. All other parameters are default. The tool */usr/bin/time* is also used to record the runtime and memory usage during the query.

- StrainEst v1.2.4

  Database construction

  The StrainEst does not provide a script for automating the construction of the database, so we implement it step by step according to the description of its paper.

```
strainest mapgenomes A1.fasta A2.fasta... SR.fasta MA.fasta
strainest map2snp SR.fasta MA.fasta snp.dgrp
strainest snpdist snp.dgrp snp_dist.txt hist.pdf
strainest snpclust snp.dgrp snp_dist.txt snp_clust.dgrp clusters.txt
bowtie2-build MA.fasta MA
```

  The specific description of commands and parameters is in the paper, which is only briefly described here. SR.fasta is the reference genome or representative genome of specified species in NCBI RefSeq. Here we select all the genomes as the representative genomes of the tool, rather than only some for metagenome alignment. The tool */usr/bin/time* is used to record the runtime and memory usage during the database construction process.

  Query

```
sickle pe -f $read1.fq -r $read2.fq -t sanger -o reads1.trim.fastq -p reads2.trim.fastq
-s reads.singles.fastq -q 20
bowtie2 --very-fast --no-unal -x MA -1 reads1.trim.fastq -2 reads2.trim.fastq -S reads.sam
samtools view -b reads.sam > reads.bam
samtools sort reads.bam -o reads.sorted.bam
samtools index reads.sorted.bam
strainest est snp_clust.dgrp reads.sorted.bam $outputdir
```

  All other parameters are default. The abundance output of all genomes is in abund.txt. The tool */usr/bin/time* is also used to record the runtime and memory usage during the query.

- StrainGE v1.3.9
  Database construction
  For every genome, firstly run
  `straingst kmerize -o $strainge_db/genome.hdf5 $genome`
  `straingst kmersim --all-vs-all -t 64 -S jaccard -S subset $strainge_db/*.hdf5 > similarities.tsv`
  `straingst cluster -i similarities.tsv -d -C 0.99 -c 0.90 --clusters-out clusters.tsv $strainge_db/*.hdf5 > references_to_keep.txt`
  `straingst createdb -f references_to_keep.txt -o pan-genome-db.hdf5`
  All genomes are prepared in specified directory as strainge_db. The tool */usr/bin/time* is used to record the runtime and memory usage during the database construction process.
  Query
  `straingst kmerize -k 23 -o result.hdf5 $read1.fq $read2.fq`
  `straingst run -O -o result pan-genome-db.hdf5 result.hdf5`
  All other parameters are default. We only run the first step of StrainGE, StrainGST, to obtain the abundance. The abundance output of possible genomes is in result.strains.tsv. The tool */usr/bin/time* is also used to record the runtime and memory usage during the query.

- StrainScan v1.0.14
  Database construction
  `strainscan_build -i $input_fasta_dir -o $strainscan_db -t 64`
  All genomes are prepared in specified directory as $input_fasta_dir The tool */usr/bin/time* is used to record the runtime and memory usage during the database construction process.
  Query
  `strainscan -i $read1.fq -j $read2.fq -d $strainscan_db -o strainscan_result`
  All other parameters are default. The abundance output of possible genomes is in final_report.txt. Possible genomes abundance is obtained by normalized the `Predicted_Depth (Ab*cls_depth) column`. The tool */usr/bin/time* is also used to record the runtime and memory usage during the query.

- metaMDBG v1.1
  For PacBio HiFi data:
  `metaMDBG asm --out-dir metaMDBG_res --in-hifi $read.fq --threads 64`

  For ONT R10 data:
  `metaMDBG asm --out-dir metaMDBG_res --in-ont $read.fq --threads 64`

- hifiasm-meta v0.3-r074
  For PacBio HiFi data:
  `hifiasm_meta -t 64 -o hifiasm_res $read.fq`

- myloasm v0.1.0
  For PacBio HiFi data:
  `myloasm $read.fq -o myloasm_res -t 64 --hifi`

  For ONT R10 data:
  `myloasm $read.fq -o myloasm_res -t 64`

- (meta)flye v2.9.6-b1802
  For PacBio HiFi data:

```
flye --pacbio-hifi $read.fq --out-dir flye_res --threads 64 --meta
```

For ONT R10 data:

```
flye --nano-hq $read.fq --out-dir flye_res --threads 64 --meta
```

References

Baaijens JA, Stougie L, and Schönhuth A 2020. Strain-aware assembly of genomes from mixed samples using flow variation graphs. In: *International Conference on Research in Computational Molecular Biology*. Springer, pp. 221–222.

Campos-Madueno EI, Aldeia C, and Endimiani A. 2024. Nanopore R10. 4 metagenomic detection of bla CTX-M/bla DHA antimicrobial resistance genes and their genetic environments in stool. *Nature Communications*. **15**: 7450.

Chen L, Zhao N, Cao J, Liu X, Xu J, Ma Y, Yu Y, Zhang X, Zhang W, Guan X, et al. 2022. Short-and long-read metagenomics expand individualized structural variations in gut microbiomes. *Nature Communications*. **13**: 3175.

Chen S. 2023. Ultrafast one-pass FASTQ data preprocessing, quality control, and deduplication using fastp. *Imeta*. **2**: e107.

De Coster W, D'hert S, Schultz DT, Cruts M, and Van Broeckhoven C. 2018. NanoPack: visualizing and processing long-read sequencing data. *Bioinformatics*. **34**: 2666–2669.

Emiola A, Zhou W, and Oh J. 2020. Metagenomic growth rate inferences of strains in situ. *Science Advances*. **6**: eaaz2299.

Feng X, Cheng H, Portik D, and Li H. 2022. Metagenome assembly of high-fidelity long reads with hifiasm-meta. *Nature Methods*. **19**: 671–674.

Fritz A, Hofmann P, Majda S, Dahms E, Dröge J, Fiedler J, Lesker TR, Belmann P, DeMaere MZ, Darling AE, et al. 2019. CAMISIM: simulating metagenomes and microbial communities. *Microbiome*. **7**: 1–12.

Garrison E, Guarracino A, Heumos S, Villani F, Bao Z, Tattini L, Hagmann J, Vorbrugg S, Marco-Sola S, Kubica C, et al. 2024. Building pangenome graphs. *Nature Methods*. **21**: 2008–2012.

Garrison E, Sirén J, Novak AM, Hickey G, Eizenga JM, Dawson ET, Jones W, Garg S, Markello C, Lin MF, et al. 2018. Variation graph toolkit improves read mapping by representing genetic variation in the reference. *Nature biotechnology*. **36**: 875–879.

Gurobi Optimization L 2023. Gurobi Optimizer Reference Manual.

Huangfu Q and Hall JJ. 2018. Parallelizing the dual revised simplex method. *Mathematical Programming Computation*. **10**: 119–142.

Jain C, Rodriguez-R LM, Phillippy AM, Konstantinidis KT, and Aluru S. 2018. High throughput ANI analysis of 90K prokaryotic genomes reveals clear species boundaries. *Nature communications*. **9**: 1–8.

Jin H, You L, Zhao F, Li S, Ma T, Kwok LY, Xu H, and Sun Z. 2022. Hybrid, ultra-deep metagenomic sequencing enables genomic and functional characterization of low-abundance species in the human gut microbiome. *Gut Microbes*. **14**: 2021790.

Kim CY, Ma J, and Lee I. 2022. HiFi metagenomic sequencing enables assembly of accurate and complete genomes from human gut microbiota. *Nature communications.* **13**: 6367.

Li H. 2018. Minimap2: pairwise alignment for nucleotide sequences. *Bioinformatics.* **34**: 3094–3100.

Liao WW, Asri M, Ebler J, Doerr D, Haukness M, Hickey G, Lu S, Lucas JK, Monlong J, Abel HJ, et al. 2023. A draft human pangenome reference. *Nature.* **617**: 312–324.

Lougee-Heimer R. 2003. The common optimization interface for operations research: Promoting open-source software in the operations research community. *IBM Journal of Research and Development.* **47**: 57–66.

Luo X, Kang X, and Schönhuth A. 2022. Strainline: full-length de novo viral haplotype reconstruction from noisy long reads. *Genome biology.* **23**: 1–27.

Makhorin A. 2008. GLPK (GNU linear programming kit). *http://www. gnu. org/s/glpk/glpk. html.*

Nicholls SM, Quick JC, Tang S, and Loman NJ. 2019. Ultra-deep, long-read nanopore sequencing of mock microbial community standards. *Gigascience.* **8**: giz043.

O'Leary NA, Wright MW, Brister JR, Ciufo S, Haddad D, McVeigh R, Rajput B, Robbertse B, Smith-White B, Ako-Adjei D, et al. 2016. Reference sequence (RefSeq) database at NCBI: current status, taxonomic expansion, and functional annotation. *Nucleic acids research.* **44**: D733–D745.

Ono Y, Asai K, and Hamada M. 2021. PBSIM2: a simulator for long-read sequencers with a novel generative model of quality scores. *Bioinformatics.* **37**: 589–595.

Sereika M, Kirkegaard RH, Karst SM, Michaelsen TY, Sørensen EA, Wollenberg RD, and Albertsen M. 2022. Oxford Nanopore R10. 4 long-read sequencing enables the generation of near-finished bacterial genomes from pure cultures and metagenomes without short-read or reference polishing. *Nature methods.* **19**: 823–826.

Simon HY, Siddle KJ, Park DJ, and Sabeti PC. 2019. Benchmarking metagenomics tools for taxonomic classification. *Cell.* **178**: 779–794.

Sun Z, Huang S, Zhang M, Zhu Q, Haiminen N, Carrieri AP, Vázquez-Baeza Y, Parida L, Kim HC, Knight R, et al. 2021. Challenges in benchmarking metagenomic profilers. *Nature methods.* **18**: 618–626.

Wallen ZD, Demirkan A, Twa G, Cohen G, Dean MN, Standaert DG, Sampson TR, and Payami H. 2022. Metagenomics of Parkinson's disease implicates the gut microbiome in multiple disease mechanisms. *Nature communications.* **13**: 6958.

Zhu K, Schäffer AA, Robinson W, Xu J, Ruppin E, Ergun AF, Ye Y, and Sahinalp SC. 2022. Strain level microbial detection and quantification with applications to single cell metagenomics. *Nature Communications.* **13**: 6430.
