# Supplementary material for: Strain-level metagenomic profiling using pangenome graphs with PanTax
Source: Genome Res. 2026 Feb;36(2):405–20. doi: 10.1101/gr.280858.125 (PMC12863173; doi:10.1101/gr.280858.125)
Supplement: Supplement 3 [file Supplemental_Tables.pdf]

Strain-level metagenomic profiling using pangenome graphs with PanTax

Wenhai Zhang[1,2,†], Yuansheng Liu[3,†], Guangyi Li[2,†], Jialu Xu[2], Enlian Chen[2], Alexander Schönhuth[4,*] Xiao Luo[1,2,*]

[1] Hunan Research Center of the Basic Discipline for Cell Signaling, Hunan University, Changsha, China
[2] College of Biology, Hunan University, Changsha, China
[3] College of Computer Science and Electronic Engineering, Hunan University, Changsha, China
[4] Faculty of Technology, Bielefeld University, Bielefeld, Germany
[†]These authors contributed equally to the work.
[*]To whom correspondence should be addressed.
Email: aschoen@cebitec.uni-bielefeld.de
Email: xluo@hnu.edu.cn

Supplemental Tables

Methods	Target References	Data Types	Strain Level	Broadly Multiple Species	Custom Database	Taxonomic/ Sequence Abundance
PanTax	WG	SR; LR	Yes	Yes	Yes	Yes/No
Sylph	WG	SR; LR	No	Yes	Yes	Yes/Yes
KMCP	WG	SR*; LR	Yes	Yes	Yes	Yes/No
Ganon	WG	SR*; LR	Yes	Yes	Yes	Yes/Yes
Centrifuge	WG	SR; LR	Yes	Yes	Yes	Yes/Yes
Centrifuger	WG	SR; LR	Yes	Yes	Yes	Yes/Yes
KrakenUniq	WG	SR*; LR	Yes	Yes	Yes	No/Yes
Kraken2	WG	SR*; LR	Yes	Yes	Yes	No/Yes
Bracken	WG	SR	Yes	Yes	Yes	No/Yes
Metamaps	WG	LR	Yes	Yes	Yes	No/Yes
CAMMiQ	WG	SR	Yes	Yes	Yes	Yes/No
Qmatey	WG	SR	Yes	Yes	Yes	Yes/Yes
CLARK	WG	SR; LR	No	Yes	Yes	No/Yes
StrainScan	WG	SR	Yes	No	Yes	Yes/No
StrainGE	WG	SR	Yes	No	Yes	Yes/No
StrainEst	WG	SR	Yes	No	Yes	Yes/No
MetaPhlAn4	Markers	SR;LR	No	Yes	No	Yes/No
MMseqs2	CDS	SR; LR	No	Yes	Yes	No/Yes
Kaiju	CDS	SR	No	Yes	Yes	No/Yes

Supplemental Table S1. Characteristic summary of representative taxonomic classifiers. WG: whole genome. CDS: coding sequence. * indicates that the tool was primarily developed and tested for short reads (SR) but can also be successfully ran on long reads (LR).

Methods	Precision	Recall	F1 score	AUPR	AFE	RFE	L1 distance	L2 distance	BC distance
Illumina									
PanTax	*0.933*	**0.933**	**0.933**	**0.930**	*0.003*	*0.188*	**0.200**	*0.047*	**0.100**
PanTax (fast)	**0.948**	*0.917*	*0.932*	0.913	*0.003*	0.212	*0.213*	0.048	*0.107*
Ganon	0.236	**0.933**	0.377	0.913	0.005	0.382	0.329	0.053	0.165
KMCP	0.767	0.767	0.767	0.739	0.008	0.598	0.550	0.100	0.275
Kraken2	0.033	**0.933**	0.063	0.908	0.011	0.693	0.721	0.113	0.361
Bracken	0.128	**0.933**	0.224	0.914	**0.002**	**0.151**	0.254	**0.043**	0.127
Centrifuge	0.104	**0.933**	0.187	*0.918*	0.010	0.646	0.664	0.109	0.332
Centrifuger	0.158	**0.933**	0.270	**0.930**	0.004	0.356	0.289	0.074	0.144
PacBio HiFi									
PanTax	**1.000**	*0.917*	**0.957**	0.917	0.004	0.212	0.222	0.063	0.111
PanTax (fast)	**1.000**	*0.917*	**0.957**	0.917	0.004	0.213	0.218	0.062	0.109
Ganon	0.615	**0.933**	*0.742*	0.919	0.003	0.205	0.220	0.040	0.110
KMCP	*0.960*	0.400	0.565	0.385	0.016	1.257	1.011	0.161	0.505
Kraken2	0.352	**0.933**	0.511	0.918	0.003	0.239	0.226	0.071	0.113
Centrifuge	0.549	**0.933**	0.691	**0.930**	*0.002*	0.124	0.181	0.047	0.091
Centrifuger	0.519	**0.933**	0.667	*0.928*	**0.001**	*0.117*	*0.137*	*0.035*	*0.068*
MetaMaps	0.609	**0.933**	0.737	0.920	**0.001**	**0.072**	**0.106**	**0.031**	**0.053**
PacBio CLR									
PanTax	*0.982*	**0.933**	**0.957**	**0.927**	**0.003**	*0.184*	**0.163**	**0.038**	**0.081**
PanTax (fast)	**1.000**	0.800	*0.889*	0.800	0.005	0.350	0.288	0.067	0.144
Ganon	-	-	-	-	-	-	-	-	-
KMCP	-	-	-	-	-	-	-	-	-
Kraken2	0.200	**0.933**	0.329	0.907	*0.004*	0.320	0.375	0.072	0.187
Centrifuge	0.033	**0.933**	0.064	*0.922*	**0.003**	**0.168**	0.268	0.057	0.134
Centrifuger	0.293	**0.933**	0.446	0.919	**0.003**	0.204	0.242	0.056	0.121
MetaMaps	0.679	*0.917*	0.780	0.893	**0.003**	0.247	*0.221*	*0.046*	*0.111*
ONT R9.4.1									
PanTax	*0.982*	**0.933**	**0.957**	*0.927*	*0.003*	0.195	**0.176**	*0.039*	**0.088**
PanTax (fast)	**1.000**	*0.900*	*0.947*	0.900	0.004	0.254	0.228	0.056	0.114
Ganon	0.675	**0.933**	0.783	0.925	0.016	0.938	0.936	0.151	0.877
KMCP	**1.000**	0.317	0.481	0.308	0.021	1.337	1.262	0.208	0.631
Kraken2	0.114	**0.933**	0.203	0.918	0.004	0.283	0.301	0.070	0.151
Centrifuge	0.062	**0.933**	0.115	**0.929**	*0.003*	**0.154**	0.221	0.052	0.111
Centrifuger	0.194	**0.933**	0.322	0.922	*0.003*	0.233	0.257	0.055	0.129
MetaMaps	0.533	**0.933**	0.679	0.917	**0.002**	*0.177*	*0.177*	**0.038**	*0.089*
ONT R10.4									
PanTax	*0.982*	**0.933**	*0.957*	0.927	*0.003*	0.188	0.192	0.045	0.096
PanTax (fast)	**1.000**	**0.933**	**0.966**	**0.933**	*0.003*	0.187	*0.178*	*0.044*	*0.089*
Ganon	0.560	**0.933**	0.700	0.920	0.006	0.408	0.400	0.060	0.243
KMCP	0.911	*0.683*	0.781	0.615	0.008	0.592	0.588	0.112	0.294
Kraken2	0.143	**0.933**	0.248	0.919	*0.003*	0.268	0.280	0.069	0.140
Centrifuge	0.170	**0.933**	0.288	*0.930*	**0.002**	*0.139*	0.202	0.050	0.101
Centrifuger	0.258	**0.933**	0.404	0.925	**0.002**	0.207	0.214	0.051	0.107
MetaMaps	0.528	**0.933**	0.675	0.918	**0.002**	**0.125**	**0.139**	**0.033**	**0.069**

Supplemental Table S2. Benchmarking results of strain-level taxonomic profiling on the sim-low datasets. Note that the best score is marked in bold, and the second best score is marked in italics. AUPR: area under the precision-recall curve. Note that we failed to run Ganon and KMCP on sim-low PacBio CLR dataset. KMCP failed because all reference genomes were filtered out, while Ganon failed as no reads matched any reference genome.

Methods	Precision	Recall	F1 score	AUPR	AFE	RFE	L1 distance	L2 distance	BC distance
Illumina									
PanTax	**0.863**	0.778	**0.818**	0.698	**0.000**	0.372	*0.470*	*0.021*	*0.235*
PanTax (fast)	**0.863**	0.778	**0.818**	0.705	**0.000**	*0.368*	**0.466**	*0.021*	**0.233**
Ganon	0.215	*0.791*	0.339	0.721	*0.001*	0.513	0.626	0.024	0.318
KMCP	*0.559*	0.757	*0.643*	0.718	**0.000**	0.398	0.578	*0.021*	0.289
Kraken2	0.081	0.788	0.147	0.725	*0.001*	0.685	0.826	0.027	0.413
Bracken	0.134	0.786	0.230	**0.744**	**0.000**	**0.279**	0.475	**0.018**	0.237
Centrifuge	0.075	0.788	0.137	0.727	*0.001*	0.647	0.794	0.026	0.397
Centrifuger	0.204	**0.792**	0.325	*0.737*	**0.000**	0.463	0.613	0.022	0.307
PacBio HiFi									
PanTax	**0.945**	0.775	**0.852**	0.714	**0.000**	0.404	0.451	0.021	0.226
PanTax (fast)	*0.940*	0.778	*0.851*	0.712	**0.000**	0.401	0.450	0.021	0.225
Ganon	0.463	*0.789*	0.584	0.728	**0.000**	0.302	**0.425**	*0.019*	**0.217**
KMCP	0.884	0.479	0.621	0.412	*0.001*	0.900	0.982	0.038	0.491
Kraken2	0.246	0.787	0.375	0.727	**0.000**	0.319	0.508	*0.019*	0.254
Centrifuge	0.298	0.787	0.432	*0.739*	**0.000**	0.284	0.451	**0.018**	0.225
Centrifuger	0.310	**0.792**	0.445	**0.745**	**0.000**	**0.274**	*0.437*	**0.018**	*0.218*
MetaMaps	0.354	0.772	0.485	0.705	**0.000**	*0.283*	0.447	*0.019*	0.224
PacBio CLR									
PanTax	**0.927**	0.783	**0.849**	0.714	**0.000**	0.392	**0.448**	0.021	**0.224**
PanTax (fast)	*0.915*	0.739	*0.817*	0.673	**0.000**	0.445	*0.507*	0.022	*0.254*
Ganon	-	-	-	-	-	-	-	-	-
KMCP	-	-	-	-	-	-	-	-	-
Kraken2	0.172	*0.788*	0.282	0.719	**0.000**	0.412	0.632	*0.020*	0.316
Centrifuge	0.159	*0.788*	0.264	*0.738*	**0.000**	*0.355*	0.549	**0.019**	0.275
Centrifuger	0.237	**0.795**	0.365	**0.746**	**0.000**	**0.311**	0.519	**0.019**	0.260
MetaMaps	0.399	0.771	0.526	0.682	**0.000**	0.400	0.536	0.024	0.268
ONT R9.4.1									
PanTax	*0.924*	0.785	**0.849**	0.719	**0.000**	0.385	**0.440**	0.020	**0.220**
PanTax (fast)	0.922	0.783	*0.847*	0.721	**0.000**	0.384	*0.441*	0.020	*0.221*
Ganon	0.533	*0.789*	0.636	**0.752**	*0.001*	0.946	0.950	0.031	0.895
KMCP	**0.950**	0.362	0.524	0.347	*0.001*	1.187	1.210	0.046	0.605
Kraken2	0.127	0.788	0.219	0.729	**0.000**	0.364	0.565	0.020	0.283
Centrifuge	0.143	0.788	0.243	0.742	**0.000**	**0.322**	0.499	**0.018**	0.249
Centrifuger	0.176	**0.795**	0.289	*0.749*	**0.000**	*0.339*	0.541	*0.019*	0.271
MetaMaps	0.248	0.772	0.375	0.694	**0.000**	0.371	0.513	0.022	0.257
ONT R10.4									
PanTax	*0.932*	0.786	*0.853*	0.729	**0.000**	0.395	*0.443*	0.021	*0.222*
PanTax (fast)	**0.938**	0.786	**0.855**	0.731	**0.000**	0.392	**0.438**	0.021	**0.219**
Ganon	0.371	*0.790*	0.505	0.739	**0.000**	0.488	0.551	*0.019*	0.342
KMCP	0.831	0.721	0.772	0.644	**0.000**	0.465	0.573	0.024	0.287
Kraken2	0.134	0.788	0.230	0.730	**0.000**	0.359	0.552	*0.019*	0.276
Centrifuge	0.159	0.787	0.264	*0.743*	**0.000**	**0.316**	0.487	**0.018**	0.243
Centrifuger	0.193	**0.794**	0.311	**0.749**	**0.000**	*0.325*	0.508	*0.019*	0.254
MetaMaps	0.235	0.772	0.360	0.697	**0.000**	0.332	0.483	0.021	0.242

Supplemental Table S3. Benchmarking results of strain-level taxonomic profiling on the sim-high datasets. Note that the best score is marked in bold, and the second best score is marked in italics. AUPR: area under the precision-recall curve. Note that we failed to run Ganon and KMCP on sim-high PacBio CLR dataset. KMCP failed because all reference genomes were filtered out, while Ganon failed as no reads matched any reference genome.

Methods	Precision	Recall	F1 score	AUPR	AFE	RFE	L1 distance	L2 distance	BC distance
Illumina									
PanTax	*0.932*	*0.917*	*0.924*	0.912	*0.003*	0.214	*0.219*	0.049	*0.110*
PanTax (fast)	**0.949**	**0.933**	**0.941**	*0.927*	*0.003*	*0.190*	**0.197**	*0.047*	**0.098**
Ganon	0.213	**0.933**	0.347	0.913	0.005	0.379	0.323	0.052	0.163
KMCP	0.780	0.767	0.773	0.742	0.008	0.597	0.555	0.100	0.278
Kraken2	0.031	**0.933**	0.061	0.908	0.011	0.695	0.725	0.113	0.362
Bracken	0.120	**0.933**	0.212	0.913	**0.002**	**0.155**	0.263	**0.043**	0.132
Centrifuge	0.095	**0.933**	0.173	0.911	0.010	0.640	0.666	0.108	0.333
Centrifuger	0.151	**0.933**	0.259	**0.930**	0.004	0.355	0.293	0.074	0.146
PacBio HiFi									
PanTax	**1.000**	**0.933**	**0.966**	**0.933**	0.003	0.212	0.196	0.050	0.098
PanTax (fast)	**1.000**	**0.933**	**0.966**	**0.933**	0.003	0.212	0.186	0.048	0.093
Ganon	0.615	**0.933**	*0.742*	0.919	0.003	0.204	0.218	0.040	0.109
KMCP	*0.962*	*0.417*	0.581	0.411	0.015	1.196	0.944	0.150	0.472
Kraken2	0.350	**0.933**	0.509	0.919	0.003	0.239	0.228	0.071	0.114
Centrifuge	0.523	**0.933**	0.671	*0.930*	*0.002*	0.124	0.182	0.047	0.091
Centrifuger	0.528	**0.933**	0.675	0.928	**0.001**	*0.119*	*0.139*	*0.035*	*0.069*
MetaMaps	0.615	**0.933**	*0.742*	0.918	**0.001**	**0.073**	**0.106**	**0.032**	**0.053**
PacBio CLR									
PanTax	*0.949*	**0.933**	**0.941**	**0.924**	**0.003**	**0.207**	**0.214**	**0.046**	**0.107**
PanTax (fast)	**0.979**	*0.783*	*0.870*	0.783	0.005	0.381	0.314	0.066	0.157
Ganon	-	-	-	-	-	-	-	-	-
KMCP	-	-	-	-	-	-	-	-	-
Kraken2	0.059	**0.933**	0.111	0.901	0.008	0.493	0.571	0.084	0.286
Centrifuge	0.027	**0.933**	0.052	*0.906*	0.006	0.336	0.439	0.070	0.220
Centrifuger	0.100	**0.933**	0.181	0.903	*0.004*	*0.312*	0.375	0.074	0.188
MetaMaps	0.483	**0.933**	0.636	0.899	0.005	0.335	*0.296*	*0.053*	*0.148*
ONT R9.4.1									
PanTax	*0.966*	**0.933**	**0.949**	**0.930**	*0.003*	0.189	**0.174**	**0.038**	**0.087**
PanTax (fast)	**1.000**	*0.900*	*0.947*	0.900	0.004	0.252	0.221	0.054	0.110
Ganon	0.675	**0.933**	0.783	0.921	0.016	0.952	0.950	0.154	0.903
KMCP	**1.000**	0.333	0.500	0.333	0.020	1.303	1.218	0.198	0.609
Kraken2	0.115	**0.933**	0.206	0.916	0.004	0.288	0.308	0.071	0.154
Centrifuge	0.063	**0.933**	0.117	*0.928*	*0.003*	**0.157**	0.223	*0.052*	0.112
Centrifuger	0.192	**0.933**	0.318	0.921	*0.003*	0.228	0.257	0.055	0.128
MetaMaps	0.554	**0.933**	0.696	0.915	**0.002**	*0.182*	*0.181*	**0.038**	*0.091*
ONT R10.4									
PanTax	*0.982*	**0.933**	*0.957*	0.927	*0.003*	0.187	0.188	0.044	0.094
PanTax (fast)	**1.000**	**0.933**	**0.966**	**0.933**	*0.003*	0.187	*0.171*	*0.043*	*0.086*
Ganon	0.577	**0.933**	0.713	0.921	0.007	0.441	0.429	0.064	0.266
KMCP	0.930	*0.667*	0.777	0.650	0.009	0.714	0.583	0.105	0.292
Kraken2	0.151	**0.933**	0.259	0.920	0.004	0.269	0.283	0.069	0.142
Centrifuge	0.166	**0.933**	0.281	*0.930*	**0.002**	*0.140*	0.202	0.050	0.101
Centrifuger	0.255	**0.933**	0.400	0.925	**0.002**	0.201	0.210	0.051	0.105
MetaMaps	0.528	**0.933**	0.675	0.920	**0.002**	**0.128**	**0.140**	**0.033**	**0.070**

Supplemental Table S4. Benchmarking results of strain-level taxonomic profiling on the sim-low-mut1 datasets. Note that the best score is marked in bold, and the second best score is marked in italics. AUPR: area under the precision-recall curve. Note that we failed to run Ganon and KMCP on sim-low-mut1 PacBio CLR dataset. KMCP failed because all reference genomes were filtered out, while Ganon failed as no reads matched any reference genome.

Methods	Precision	Recall	F1 score	AUPR	AFE	RFE	L1 distance	L2 distance	BC distance
Illumina									
PanTax	*0.864*	0.778	*0.819*	0.700	**0.000**	0.377	*0.472*	0.021	*0.236*
PanTax (fast)	**0.867**	0.778	**0.820**	0.697	**0.000**	0.367	**0.467**	0.021	**0.234**
Ganon	0.210	*0.790*	0.332	0.722	**0.000**	0.511	0.621	0.024	0.318
KMCP	0.556	0.761	0.643	0.719	**0.000**	*0.359*	0.532	*0.020*	0.266
Kraken2	0.079	0.788	0.144	0.725	*0.001*	0.684	0.828	0.027	0.414
Bracken	0.131	0.788	0.224	**0.743**	**0.000**	**0.280**	0.478	**0.018**	0.239
Centrifuge	0.074	0.788	0.135	0.725	*0.001*	0.636	0.793	0.025	0.397
Centrifuger	0.198	**0.792**	0.317	*0.738*	**0.000**	0.462	0.615	0.022	0.308
PacBio HiFi									
PanTax	**0.943**	0.777	**0.852**	0.722	**0.000**	0.403	0.448	0.021	0.224
PanTax (fast)	**0.943**	0.776	*0.851*	0.717	**0.000**	0.403	0.447	0.021	0.223
Ganon	0.464	*0.789*	0.585	0.729	**0.000**	0.300	**0.421**	**0.018**	**0.215**
KMCP	*0.902*	0.488	0.633	0.434	*0.001*	0.911	0.968	0.037	0.484
Kraken2	0.244	0.788	0.372	0.727	**0.000**	0.319	0.509	*0.019*	0.254
Centrifuge	0.300	0.788	0.434	*0.740*	**0.000**	*0.282*	0.451	**0.018**	0.225
Centrifuger	0.304	**0.795**	0.440	**0.746**	**0.000**	**0.273**	*0.437*	**0.018**	*0.219*
MetaMaps	0.354	0.772	0.485	0.704	**0.000**	0.287	0.450	0.020	0.225
PacBio CLR									
PanTax	**0.920**	0.786	**0.848**	0.721	**0.000**	0.390	**0.447**	0.021	**0.223**
PanTax (fast)	*0.917*	0.738	*0.818*	0.666	**0.000**	0.443	*0.506*	0.022	*0.253*
Ganon	-	-	-	-	-	-	-	-	-
KMCP	-	-	-	-	-	-	-	-	-
Kraken2	0.169	*0.788*	0.278	0.719	**0.000**	0.414	0.634	*0.020*	0.317
Centrifuge	0.160	0.785	0.266	*0.737*	**0.000**	*0.356*	0.550	**0.019**	0.275
Centrifuger	0.233	**0.795**	0.360	**0.746**	**0.000**	**0.311**	0.520	**0.019**	0.260
MetaMaps	0.396	0.772	0.523	0.682	**0.000**	0.399	0.535	0.023	0.267
ONT R9.4.1									
PanTax	0.921	0.785	**0.848**	0.718	**0.000**	0.385	*0.443*	0.020	*0.221*
PanTax (fast)	*0.926*	0.783	**0.848**	0.720	**0.000**	0.381	**0.438**	0.020	**0.219**
Ganon	0.550	*0.788*	*0.648*	**0.748**	*0.001*	0.958	0.962	0.032	0.918
KMCP	**0.957**	0.352	0.515	0.337	*0.001*	1.224	1.246	0.048	0.623
Kraken2	0.128	*0.788*	0.220	0.728	**0.000**	0.365	0.567	0.020	0.283
Centrifuge	0.141	*0.788*	0.239	*0.743*	**0.000**	**0.323**	0.500	**0.018**	0.250
Centrifuger	0.175	**0.795**	0.287	**0.748**	**0.000**	*0.337*	0.540	*0.019*	0.270
MetaMaps	0.251	0.772	0.379	0.694	**0.000**	0.371	0.514	0.022	0.257
ONT R10.4									
PanTax	*0.930*	0.782	*0.850*	0.725	**0.000**	0.398	*0.449*	0.021	*0.225*
PanTax (fast)	**0.931**	0.784	**0.851**	0.724	**0.000**	0.393	**0.443**	0.021	**0.221**
Ganon	0.374	*0.790*	0.508	*0.742*	**0.000**	0.513	0.569	*0.019*	0.361
KMCP	0.833	0.731	0.778	0.643	**0.000**	0.438	0.556	0.023	0.278
Kraken2	0.135	0.788	0.230	0.731	**0.000**	0.359	0.554	*0.019*	0.277
Centrifuge	0.159	0.787	0.265	*0.742*	**0.000**	**0.317**	0.489	**0.018**	0.244
Centrifuger	0.194	**0.795**	0.312	**0.750**	**0.000**	*0.327*	0.509	*0.019*	0.254
MetaMaps	0.235	0.772	0.360	0.699	**0.000**	0.334	0.484	0.021	0.242

Supplemental Table S5. Benchmarking results of strain-level taxonomic profiling on the sim-high-mut1 datasets. Note that the best score is marked in bold, and the second best score is marked in italics. AUPR: area under the precision-recall curve. Note that we failed to run Ganon and KMCP on sim-high-mut1 PacBio CLR dataset. KMCP failed because all reference genomes were filtered out, while Ganon failed as no reads matched any reference genome.

Methods	Precision	Recall	F1 score	AUPR	AFE	RFE	L1 distance	L2 distance	BC distance
Illumina									
PanTax	*0.933*	**0.933**	**0.933**	**0.930**	**0.003**	*0.196*	**0.203**	*0.047*	**0.101**
PanTax (fast)	**0.980**	*0.833*	*0.901*	0.832	*0.004*	0.361	*0.275*	0.055	*0.138*
Ganon	0.185	**0.933**	0.309	0.914	0.007	0.493	0.426	0.063	0.261
KMCP	0.758	*0.833*	0.794	0.811	*0.004*	0.309	0.318	0.058	0.159
Kraken2	0.029	**0.933**	0.056	0.905	0.011	0.699	0.742	0.114	0.371
Bracken	0.093	**0.933**	0.169	0.912	**0.003**	**0.176**	0.302	**0.045**	0.151
Centrifuge	0.069	**0.933**	0.129	0.885	0.010	0.622	0.689	0.104	0.344
Centrifuger	0.112	**0.933**	0.201	*0.922*	*0.004*	0.346	0.326	0.075	0.163
PacBio HiFi									
PanTax	**1.000**	**0.933**	**0.966**	**0.933**	*0.002*	0.166	0.150	0.038	*0.075*
PanTax (fast)	**1.000**	*0.817*	*0.899*	0.817	0.005	0.312	0.272	0.065	0.136
Ganon	0.622	**0.933**	0.747	0.926	0.003	0.196	0.190	*0.037*	0.101
KMCP	*0.931*	0.450	0.607	0.415	0.013	0.971	0.862	0.139	0.431
Kraken2	0.327	**0.933**	0.485	0.916	0.003	0.242	0.237	0.072	0.119
Centrifuge	0.424	**0.933**	0.583	*0.928*	*0.002*	0.129	0.190	0.049	0.095
Centrifuger	0.505	**0.933**	0.655	0.923	*0.002*	*0.125*	*0.149*	*0.037*	*0.075*
MetaMaps	0.629	**0.933**	0.752	0.919	**0.001**	**0.081**	**0.110**	**0.032**	**0.055**
PacBio CLR									
PanTax	*0.933*	**0.933**	**0.933**	**0.928**	**0.003**	**0.205**	**0.206**	**0.043**	**0.103**
PanTax (fast)	**0.947**	*0.600*	*0.735*	0.593	0.009	0.619	0.542	0.089	0.271
Ganon	-	-	-	-	-	-	-	-	-
KMCP	-	-	-	-	-	-	-	-	-
Kraken2	0.048	**0.933**	0.091	0.899	0.008	0.517	0.600	0.087	0.300
Centrifuge	0.025	**0.933**	0.050	*0.900*	0.006	0.370	0.477	0.073	0.238
Centrifuger	0.078	**0.933**	0.144	0.899	*0.004*	*0.327*	0.400	0.075	0.200
MetaMaps	0.471	**0.933**	0.626	*0.900*	0.005	0.338	*0.300*	*0.053*	*0.150*
ONT R9.4.1									
PanTax	*0.982*	**0.933**	**0.957**	**0.927**	**0.003**	*0.183*	**0.169**	**0.038**	**0.085**
PanTax (fast)	**1.000**	*0.850*	*0.919*	0.850	**0.003**	0.273	0.200	0.041	0.100
Ganon	0.848	0.650	0.736	0.604	0.017	0.999	0.999	0.162	0.998
KMCP	-	-	-	-	-	-	-	-	-
Kraken2	0.111	**0.933**	0.198	0.916	*0.004*	0.298	0.318	0.071	0.159
Centrifuge	0.054	**0.933**	0.102	*0.926*	**0.003**	**0.172**	0.237	0.053	0.119
Centrifuger	0.178	**0.933**	0.299	0.920	**0.003**	0.242	0.271	0.058	0.136
MetaMaps	0.528	**0.933**	0.675	0.915	**0.003**	0.211	*0.196*	*0.040*	*0.098*
ONT R10.4									
PanTax	*0.982*	**0.933**	**0.957**	0.923	*0.003*	0.182	*0.180*	*0.042*	*0.090*
PanTax (fast)	**1.000**	*0.867*	*0.929*	0.867	0.004	0.260	0.213	0.046	0.107
Ganon	0.566	**0.933**	0.704	*0.924*	0.015	0.880	0.875	0.140	0.772
KMCP	0.976	0.683	0.804	0.671	0.009	0.718	0.569	0.115	0.284
Kraken2	0.128	**0.933**	0.226	0.918	0.004	0.277	0.294	0.070	0.147
Centrifuge	0.128	**0.933**	0.224	**0.928**	*0.003*	**0.147**	0.211	0.051	0.105
Centrifuger	0.223	**0.933**	0.360	0.923	*0.003*	0.209	0.224	0.053	0.112
MetaMaps	0.487	**0.933**	0.640	0.917	**0.002**	*0.154*	**0.159**	**0.036**	**0.079**

Supplemental Table S6. Benchmarking results of strain-level taxonomic profiling on the sim-low-mut2 datasets. Note that the best score is marked in bold, and the second best score is marked in italics. AUPR: area under the precision-recall curve. Note that we failed to run Ganon and KMCP on sim-low-mut2 PacBio CLR dataset. KMCP failed because all reference genomes were filtered out, while Ganon failed as no reads matched any reference genome.

Methods	Precision	Recall	F1 score	AUPR	AFE	RFE	L1 distance	L2 distance	BC distance
Illumina									
PanTax	**0.866**	0.777	**0.819**	0.698	**0.000**	0.377	**0.472**	0.021	**0.236**
PanTax (fast)	*0.859*	0.737	*0.793*	0.677	**0.000**	0.439	0.536	0.022	0.268
Ganon	0.204	*0.789*	0.324	0.724	*0.001*	0.563	0.612	0.021	0.382
KMCP	0.570	0.769	0.654	0.734	**0.000**	*0.337*	*0.502*	*0.019*	*0.251*
Kraken2	0.076	0.788	0.138	0.723	*0.001*	0.683	0.841	0.027	0.420
Bracken	0.116	0.788	0.203	**0.738**	**0.000**	**0.287**	0.505	**0.018**	0.252
Centrifuge	0.070	0.787	0.129	0.702	*0.001*	0.611	0.813	0.025	0.406
Centrifuger	0.174	**0.795**	0.285	*0.735*	**0.000**	0.450	0.633	0.021	0.317
PacBio HiFi									
PanTax	*0.939*	0.783	**0.854**	0.723	**0.000**	0.394	*0.441*	0.021	*0.221*
PanTax (fast)	**0.945**	0.738	*0.829*	0.675	**0.000**	0.466	0.512	0.023	0.256
Ganon	0.502	*0.789*	0.613	**0.749**	**0.000**	0.311	**0.377**	**0.017**	**0.209**
KMCP	0.897	0.496	0.639	0.434	*0.001*	0.889	0.954	0.037	0.477
Kraken2	0.238	0.788	0.365	0.724	**0.000**	0.322	0.521	0.019	0.260
Centrifuge	0.290	0.788	0.424	0.740	**0.000**	*0.289*	0.459	*0.018*	0.229
Centrifuger	0.301	**0.795**	0.437	*0.748*	**0.000**	**0.271**	0.442	*0.018*	*0.221*
MetaMaps	0.362	0.772	0.493	0.698	**0.000**	0.307	0.463	0.020	0.232
PacBio CLR									
PanTax	**0.924**	0.786	**0.849**	0.717	**0.000**	0.384	**0.442**	*0.020*	**0.221**
PanTax (fast)	*0.882*	0.516	*0.651*	0.459	*0.001*	0.835	0.930	0.033	0.465
Ganon	-	-	-	-	-	-	-	-	-
KMCP	-	-	-	-	-	-	-	-	-
Kraken2	0.166	*0.787*	0.274	0.718	**0.000**	0.427	0.650	0.021	0.325
Centrifuge	0.152	*0.787*	0.255	*0.738*	**0.000**	*0.365*	0.565	**0.019**	0.282
Centrifuger	0.230	**0.795**	0.357	**0.745**	**0.000**	**0.318**	*0.532*	**0.019**	*0.266*
MetaMaps	0.398	0.770	0.525	0.684	**0.000**	0.415	0.547	0.024	0.273
ONT R9.4.1									
PanTax	*0.925*	0.784	**0.848**	0.722	**0.000**	0.390	**0.442**	0.020	**0.221**
PanTax (fast)	**0.927**	0.747	*0.827*	0.685	**0.000**	0.441	*0.492*	0.022	*0.246*
Ganon	0.788	0.577	0.666	0.488	*0.001*	0.999	0.999	0.033	0.998
KMCP	-	-	-	-	-	-	-	-	-
Kraken2	0.125	*0.788*	0.215	0.726	**0.000**	0.373	0.578	0.020	0.289
Centrifuge	0.135	*0.788*	0.231	*0.743*	**0.000**	**0.329**	0.509	**0.018**	0.254
Centrifuger	0.172	**0.795**	0.283	**0.746**	**0.000**	*0.343*	0.551	*0.019*	0.276
MetaMaps	0.247	0.772	0.374	0.694	**0.000**	0.384	0.523	0.022	0.262
ONT R10.4									
PanTax	*0.929*	0.785	**0.851**	0.730	**0.000**	0.391	**0.443**	0.021	**0.221**
PanTax (fast)	**0.935**	0.739	*0.826*	0.678	**0.000**	0.459	0.514	0.023	0.257
Ganon	0.471	*0.789*	0.590	**0.753**	*0.001*	0.896	0.904	0.030	0.809
KMCP	0.899	0.724	0.802	0.653	**0.000**	0.484	0.552	0.024	0.276
Kraken2	0.128	0.788	0.220	0.727	**0.000**	0.364	0.565	0.020	0.283
Centrifuge	0.153	0.788	0.256	0.743	**0.000**	**0.323**	*0.496*	**0.018**	*0.248*
Centrifuger	0.188	**0.795**	0.303	*0.749*	**0.000**	*0.327*	0.517	*0.019*	0.258
MetaMaps	0.240	0.772	0.366	0.695	**0.000**	0.353	0.499	0.021	0.249

Supplemental Table S7. Benchmarking results of strain-level taxonomic profiling on the sim-high-mut2 datasets. Note that the best score is marked in bold, and the second best score is marked in italics. AUPR: area under the precision-recall curve. Note that we failed to run Ganon and KMCP on sim-high-mut2 PacBio CLR dataset. KMCP failed because all reference genomes were filtered out, while Ganon failed as no reads matched any reference genome.

Methods	Precision	Recall	F1 score	AUPR	AFE	RFE	L1 distance	L2 distance	BC distance
Zymo1 Illumina									
PanTax	**0.875**	**0.875**	**0.875**	0.788	*0.030*	*0.233*	*0.374*	0.207	*0.187*
PanTax (fast)	**0.875**	**0.875**	**0.875**	0.788	**0.029**	**0.227**	**0.367**	*0.206*	**0.183**
Ganon	0.014	**0.875**	0.027	0.788	0.039	0.273	0.438	**0.202**	0.238
KMCP	*0.462*	*0.750*	*0.571*	0.643	0.045	0.329	0.630	0.259	0.315
Kraken2	0.013	**0.875**	0.026	*0.858*	0.129	1.073	1.247	0.487	0.623
Bracken	0.013	**0.875**	0.026	**0.875**	0.051	0.350	0.810	0.209	0.405
Centrifuge	0.013	**0.875**	0.027	*0.858*	0.126	1.056	1.113	0.519	0.557
Centrifuger	0.013	**0.875**	0.026	0.839	0.044	0.301	0.666	0.210	0.333
Zymo1 ONT R9.4.1									
PanTax	*0.467*	**0.875**	*0.609*	0.751	*0.040*	0.309	0.515	0.230	0.258
PanTax (fast)	**0.583**	**0.875**	**0.700**	*0.816*	**0.039**	**0.295**	*0.474*	**0.215**	*0.237*
Ganon	0.294	*0.625*	0.400	0.283	0.125	1.000	1.000	0.378	1.000
KMCP	-	-	-	-	-	-	-	-	-
Kraken2	0.013	**0.875**	0.026	**0.858**	0.091	0.708	1.275	0.288	0.638
Centrifuge	0.013	**0.875**	0.026	0.789	0.076	0.601	0.939	0.266	0.469
Centrifuger	0.013	**0.875**	0.026	0.713	0.075	0.647	1.083	0.248	0.542
MetaMaps	0.016	**0.875**	0.032	0.788	**0.039**	*0.297*	**0.465**	*0.220*	**0.233**
Zymo1 ONT R10									
PanTax	*0.538*	**0.875**	**0.667**	*0.729*	*0.068*	*0.587*	0.755	0.280	0.377
PanTax (fast)	**0.545**	*0.750*	*0.632*	0.708	*0.068*	0.602	*0.744*	**0.262**	*0.372*
Ganon	0.000	0.000	0.000	0.000	0.125	1.000	1.000	0.378	1.000
KMCP	-	-	-	-	-	-	-	-	-
Kraken2	0.013	**0.875**	0.026	0.674	0.108	0.896	1.458	0.327	0.729
Centrifuge	0.013	**0.875**	0.026	0.680	0.095	0.827	1.133	0.318	0.566
Centrifuger	0.013	**0.875**	0.026	0.645	0.088	0.751	1.228	0.278	0.614
MetaMaps	0.016	**0.875**	0.031	**0.751**	**0.065**	**0.570**	**0.701**	*0.271*	**0.350**
Zymo2 Illumina									
PanTax	*0.625*	0.625	0.625	0.602	0.013	0.761	0.105	*0.069*	0.053
PanTax (fast)	0.600	*0.750*	*0.667*	0.697	0.013	0.737	0.110	0.072	0.055
Ganon	0.036	**0.875**	0.069	**0.761**	*0.010*	*0.365*	*0.082*	0.074	*0.042*
KMCP	**1.000**	*0.750*	**0.857**	*0.750*	**0.002**	**0.337**	**0.013**	**0.009**	**0.007**
Kraken2	0.017	**0.875**	0.033	0.397	0.087	1.953	1.191	0.607	0.595
Bracken	0.025	*0.750*	0.049	0.285	0.046	0.401	0.729	0.363	0.364
Centrifuge	0.016	**0.875**	0.032	0.430	0.058	2.533	0.601	0.327	0.300
Centrifuger	0.024	*0.750*	0.046	0.327	0.017	0.407	0.264	0.137	0.132
Zymo2 ONT R9.4.1									
PanTax	*0.667*	0.500	*0.571*	*0.454*	*0.002*	0.562	*0.020*	*0.011*	*0.010*
PanTax (fast)	**0.714**	*0.625*	**0.667**	0.432	0.009	*0.449*	0.135	0.082	0.068
Ganon	0.000	0.000	0.000	0.000	0.125	1.000	1.000	0.951	1.000
KMCP	-	-	-	-	-	-	-	-	-
Kraken2	0.017	**0.875**	0.034	0.174	0.105	0.698	1.667	0.821	0.833
Centrifuge	0.015	**0.875**	0.029	0.246	0.059	0.997	0.928	0.485	0.464
Centrifuger	0.016	**0.875**	0.031	0.168	0.072	0.639	1.155	0.575	0.577
MetaMaps	0.019	**0.875**	0.037	**0.696**	**0.001**	**0.266**	**0.008**	**0.004**	**0.004**

Supplemental Table S8. Benchmarking results of strain-level taxonomic profiling on the Zymo datasets. Note that the best score is marked in bold, and the second best score is marked in italics. AUPR: area under the precision-recall curve. Note that we failed to run KMCP on Zymo1 ONT and Zymo2 ONT dataset. KMCP failed because all reference genomes were filtered out, while Ganon failed as no reads matched any reference genome.

Methods	Precision	Recall	F1 score	AUPR	AFE	RFE	L1 distance	L2 distance	BC distance
Illumina									
PanTax	**1.000**	**1.000**	**1.000**	**1.000**	*0.011*	0.098	0.092	0.036	0.046
Ganon	*0.800*	**1.000**	*0.889*	**1.000**	0.049	0.484	0.395	0.167	0.198
KMCP	**1.000**	**1.000**	**1.000**	**1.000**	*0.011*	*0.094*	*0.086*	*0.035*	*0.043*
Kraken2	*0.800*	**1.000**	*0.889*	**1.000**	0.044	0.411	0.356	0.138	0.178
Bracken	*0.800*	**1.000**	*0.889*	**1.000**	**0.006**	**0.050**	**0.051**	**0.018**	**0.026**
Centrifuge	*0.800*	**1.000**	*0.889*	*0.985*	0.041	0.400	0.354	0.127	0.177
Centrifuger	*0.800*	**1.000**	*0.889*	**1.000**	0.044	0.412	0.351	0.131	0.175
PacBio HiFi									
PanTax	**1.000**	**1.000**	**1.000**	**1.000**	*0.011*	0.113	0.088	0.036	0.044
Ganon	**1.000**	**1.000**	**1.000**	**1.000**	0.014	0.166	0.110	0.065	0.055
KMCP	**1.000**	*0.750*	0.857	*0.750*	0.047	0.518	0.379	0.166	0.190
Kraken2	*0.800*	**1.000**	*0.889*	**1.000**	0.012	0.118	0.106	0.037	0.053
Centrifuge	**1.000**	**1.000**	**1.000**	**1.000**	**0.005**	*0.049*	*0.041*	*0.015*	*0.020*
Centrifuger	**1.000**	**1.000**	**1.000**	**1.000**	**0.005**	**0.044**	**0.038**	**0.014**	**0.019**
MetaMaps	0.667	0.500	0.571	0.500	0.115	1.034	0.974	0.374	0.487
PacBio CLR									
PanTax	**1.000**	**1.000**	**1.000**	**1.000**	**0.008**	**0.072**	**0.060**	**0.024**	**0.030**
Ganon	-	-	-	-	-	-	-	-	-
KMCP	-	-	-	-	-	-	-	-	-
Kraken2	*0.800*	**1.000**	*0.889*	**1.000**	0.028	0.280	0.252	0.088	0.126
Centrifuge	*0.800*	**1.000**	*0.889*	**1.000**	0.022	0.208	0.177	0.065	0.088
Centrifuger	*0.800*	**1.000**	*0.889*	**1.000**	*0.016*	*0.154*	*0.132*	*0.049*	*0.066*
MetaMaps	0.667	*0.500*	0.571	*0.500*	0.121	1.109	0.974	0.413	0.487
ONT R9.4.1									
PanTax	**1.000**	**1.000**	**1.000**	**1.000**	**0.016**	**0.145**	**0.128**	**0.048**	**0.064**
Ganon	**1.000**	**1.000**	**1.000**	**1.000**	0.026	0.290	0.208	0.112	0.104
KMCP	**1.000**	0.250	0.400	0.250	0.153	1.147	1.223	0.552	0.612
Kraken2	*0.800*	**1.000**	*0.889*	**1.000**	0.021	0.205	0.181	0.064	0.091
Centrifuge	*0.800*	**1.000**	*0.889*	**1.000**	*0.018*	*0.167*	*0.142*	*0.052*	*0.071*
Centrifuger	*0.800*	**1.000**	*0.889*	**1.000**	0.027	0.263	0.228	0.082	0.114
MetaMaps	0.667	*0.500*	0.571	*0.500*	0.120	1.086	0.974	0.399	0.487
ONT R10.4									
PanTax	**1.000**	**1.000**	**1.000**	**1.000**	**0.006**	**0.059**	**0.050**	**0.022**	**0.025**
Ganon	**1.000**	**1.000**	**1.000**	**1.000**	0.024	0.269	0.190	0.096	0.095
KMCP	**1.000**	**1.000**	**1.000**	**1.000**	*0.010*	*0.093*	*0.082*	*0.039*	*0.041*
Kraken2	*0.800*	**1.000**	*0.889*	**1.000**	0.021	0.202	0.177	0.062	0.089
Centrifuge	*0.800*	**1.000**	*0.889*	**1.000**	0.017	0.158	0.133	0.049	0.067
Centrifuger	*0.800*	**1.000**	*0.889*	**1.000**	0.028	0.264	0.223	0.083	0.112
MetaMaps	0.667	*0.500*	0.571	*0.500*	0.118	1.061	0.974	0.381	0.487

Supplemental Table S9. Benchmarking results of strain-level taxonomic profiling on the spiked-in datasets. Note that the best score is marked in bold, and the second best score is marked in italics. AUPR: area under the precision-recall curve. Note that we failed to run Ganon and KMCP on spiked-in PacBio CLR dataset. KMCP failed because all reference genomes were filtered out, while Ganon failed as no reads matched any reference genome.

Methods	Precision	Recall	F1 score	AUPR	AFE	RFE	L1 distance	L2 distance	BC distance
3 strains									
PanTax	**1.000**	**1.000**	**1.000**	**1.000**	**0.000**	**0.000**	**0.000**	**0.000**	**0.000**
StrainScan	**1.000**	*0.667*	*0.800*	*0.667*	0.074	0.427	0.222	0.137	0.111
StrainGE	**1.000**	**1.000**	**1.000**	**1.000**	0.068	0.152	*0.204*	0.152	*0.106*
StrainEst	*0.176*	**1.000**	0.300	**1.000**	*0.047*	*0.137*	0.279	*0.130*	0.140
5 strains									
PanTax	**1.000**	**1.000**	**1.000**	**1.000**	**0.000**	**0.000**	**0.000**	**0.000**	**0.000**
StrainScan	**1.000**	*0.800*	*0.889*	*0.800*	*0.019*	0.287	*0.097*	*0.058*	*0.048*
StrainGE	**1.000**	**1.000**	**1.000**	**1.000**	0.050	0.221	0.249	0.136	0.128
StrainEst	*0.294*	**1.000**	0.455	**1.000**	0.020	*0.155*	0.198	0.071	0.099
10 strains									
PanTax	**1.000**	**1.000**	**1.000**	**1.000**	**0.004**	**0.041**	**0.041**	**0.017**	**0.021**
StrainScan	**1.000**	*0.900*	*0.947*	*0.900*	0.013	0.250	0.127	0.044	0.063
StrainGE	**1.000**	0.500	0.667	0.500	0.051	0.659	0.513	0.186	0.266
StrainEst	*0.357*	**1.000**	0.526	**1.000**	*0.005*	*0.103*	*0.103*	*0.026*	*0.051*

Supplemental Table S10. Benchmarking results of strain-level taxonomic profiling on the *S. epidermidis* strain mixtures datasets. Note that the best score is marked in bold, and the second best score is marked in italics.

Methods	Precision	Recall	F1 score	AUPR	AFE	RFE	L1 distance	L2 distance	BC distance
Illumina									
PanTax	0.667	**0.933**	*0.778*	*0.923*	*0.003*	0.291	0.276	*0.046*	0.138
PanTax (fast)	*0.705*	*0.917*	**0.797**	0.903	*0.003*	*0.288*	*0.267*	*0.046*	*0.134*
Ganon	0.276	**0.933**	0.426	0.913	0.005	0.381	0.329	0.053	0.165
KMCP	**0.786**	0.733	0.759	0.709	0.008	0.573	0.531	0.094	0.266
Kraken2	0.042	**0.933**	0.081	0.908	0.011	0.693	0.721	0.112	0.360
Bracken	0.171	**0.933**	0.289	0.913	**0.002**	**0.150**	**0.252**	**0.043**	**0.126**
Centrifuge	0.129	**0.933**	0.227	0.918	0.010	0.646	0.663	0.109	0.331
Centrifuger	0.159	**0.933**	0.272	**0.930**	0.004	0.356	0.289	0.074	0.144
PacBio HiFi									
PanTax	0.709	**0.933**	*0.806*	*0.929*	0.003	0.287	0.268	0.050	0.134
PanTax (fast)	*0.740*	*0.900*	**0.812**	0.878	0.004	0.306	0.287	0.060	0.143
Ganon	0.636	**0.933**	0.757	0.920	0.003	0.207	0.219	0.040	0.110
KMCP	**1.000**	0.283	0.442	0.283	0.021	1.497	1.231	0.210	0.616
Kraken2	0.368	**0.933**	0.528	0.917	0.003	0.264	0.243	0.072	0.122
Centrifuge	0.554	**0.933**	0.696	**0.931**	*0.002*	*0.136*	0.186	0.047	0.093
Centrifuger	0.544	**0.933**	0.687	*0.929*	*0.002*	0.144	*0.155*	*0.034*	*0.078*
MetaMaps	0.629	**0.933**	0.752	0.920	**0.001**	**0.103**	**0.128**	**0.032**	**0.064**
PacBio CLR									
PanTax	0.615	**0.933**	*0.742*	0.903	*0.004*	0.296	0.334	**0.046**	0.167
PanTax (fast)	*0.671*	0.783	0.723	0.774	0.005	0.390	0.372	0.066	0.186
Ganon	-	-	-	-	-	-	-	-	-
KMCP	-	-	-	-	-	-	-	-	-
Kraken2	0.232	**0.933**	0.372	0.905	0.005	0.338	0.387	0.073	0.194
Centrifuge	0.052	**0.933**	0.099	**0.921**	**0.003**	**0.189**	0.277	0.058	0.138
Centrifuger	0.320	**0.933**	0.477	*0.914*	**0.003**	*0.234*	*0.263*	*0.057*	*0.131*
MetaMaps	**0.730**	*0.900*	**0.806**	0.879	**0.003**	0.258	**0.229**	**0.046**	**0.115**
ONT R9.4.1									
PanTax	0.644	**0.933**	0.762	0.912	**0.003**	0.268	0.292	0.044	0.146
PanTax (fast)	*0.720*	*0.900*	**0.800**	0.886	**0.003**	0.284	0.267	*0.042*	0.133
Ganon	0.667	**0.933**	*0.778*	*0.925*	0.016	0.938	0.937	0.151	0.878
KMCP	**1.000**	0.167	0.286	0.167	0.025	1.478	1.472	0.285	0.736
Kraken2	0.146	**0.933**	0.252	0.920	*0.004*	0.296	0.308	0.070	0.154
Centrifuge	0.093	**0.933**	0.170	**0.928**	**0.003**	**0.173**	*0.234*	0.052	*0.117*
Centrifuger	0.224	**0.933**	0.361	0.923	**0.003**	0.241	0.260	0.056	0.130
MetaMaps	0.560	**0.933**	0.700	0.917	**0.003**	*0.204*	**0.192**	**0.038**	**0.096**
ONT R10.4									
PanTax	0.659	**0.933**	**0.772**	0.922	*0.003*	0.280	0.282	*0.049*	0.141
PanTax (fast)	*0.680*	*0.850*	*0.756*	0.842	0.004	0.321	0.304	0.061	0.152
Ganon	0.602	**0.933**	0.732	0.918	0.006	0.409	0.400	0.060	0.242
KMCP	**0.972**	0.583	0.729	0.574	0.011	0.845	0.682	0.116	0.341
Kraken2	0.177	**0.933**	0.297	0.919	*0.003*	0.273	0.280	0.069	0.140
Centrifuge	0.234	**0.933**	0.375	**0.929**	*0.003*	**0.151**	*0.211*	0.050	*0.105*
Centrifuger	0.295	**0.933**	0.448	*0.924*	*0.003*	0.216	0.221	0.051	0.110
MetaMaps	0.577	**0.933**	0.713	0.918	**0.002**	*0.162*	**0.161**	**0.035**	**0.080**

Supplemental Table S11. Benchmarking results of strain-level taxonomic profiling on the sim-low-sub1 datasets. Note that the best score is marked in bold, and the second best score is marked in italics. AUPR: area under the precision-recall curve. Note that we failed to run Ganon and KMCP on sim-low-sub1 PacBio CLR dataset. KMCP failed because all reference genomes were filtered out, while Ganon failed as no reads matched any reference genome. To account for the presence of low-coverage(0.5×) strains, PanTax adopts a more relaxed threshold on this dataset by setting f_{strain} to 0 and d_{strain} to 1.

Methods	Precision	Recall	F1 score	AUPR	AFE	RFE	L1 distance	L2 distance	BC distance
Illumina									
PanTax	0.720	*0.900*	**0.800**	0.886	0.005	0.536	0.401	0.061	0.200
PanTax (fast)	*0.742*	0.767	0.754	0.763	0.006	0.549	0.397	0.070	0.198
Ganon	0.341	**0.933**	0.500	0.913	0.005	0.383	0.330	*0.053*	0.166
KMCP	**0.863**	0.733	*0.793*	0.710	0.008	0.577	0.520	0.095	0.260
Kraken2	0.062	**0.933**	0.116	0.908	0.011	0.693	0.720	0.113	0.360
Bracken	0.232	**0.933**	0.372	0.913	**0.002**	**0.147**	**0.246**	**0.043**	**0.123**
Centrifuge	0.172	**0.933**	0.290	*0.918*	0.010	0.645	0.660	0.109	0.330
Centrifuger	0.198	**0.933**	0.327	**0.930**	*0.004*	*0.354*	*0.288*	0.074	*0.144*
PacBio HiFi									
PanTax	0.797	*0.850*	*0.823*	0.847	0.006	0.585	0.431	0.066	0.216
PanTax (fast)	*0.850*	*0.850*	**0.850**	0.834	0.006	0.537	0.406	0.063	0.203
Ganon	0.667	**0.933**	0.778	0.917	*0.003*	0.223	0.224	0.040	0.112
KMCP	**1.000**	0.117	0.209	0.117	0.027	1.768	1.615	0.343	0.807
Kraken2	0.412	**0.933**	0.571	0.917	*0.003*	0.290	0.259	0.071	0.130
Centrifuge	0.577	**0.933**	0.713	**0.927**	*0.003*	*0.163*	0.202	0.048	0.101
Centrifuger	0.571	**0.933**	0.709	*0.926*	**0.002**	0.172	*0.175*	*0.035*	*0.088*
MetaMaps	0.659	**0.933**	0.772	0.919	**0.002**	**0.141**	**0.152**	**0.032**	**0.076**
PacBio CLR									
PanTax	**0.783**	*0.900*	**0.837**	0.858	0.006	0.614	0.476	0.069	0.238
PanTax (fast)	*0.771*	0.617	0.685	0.568	0.009	0.719	0.633	0.116	0.317
Ganon	-	-	-	-	-	-	-	-	-
KMCP	-	-	-	-	-	-	-	-	-
Kraken2	0.287	**0.933**	0.439	0.904	0.005	0.345	0.386	0.073	0.193
Centrifuge	0.098	**0.933**	0.177	**0.919**	**0.003**	**0.209**	*0.286*	*0.058*	*0.143*
Centrifuger	0.386	**0.933**	0.546	*0.912*	**0.003**	*0.266*	0.288	0.059	0.144
MetaMaps	0.761	*0.900*	*0.824*	0.881	*0.004*	0.278	**0.245**	**0.048**	**0.123**
ONT R9.4.1									
PanTax	0.697	*0.883*	*0.779*	0.856	0.006	0.544	0.458	0.064	0.229
PanTax (fast)	*0.754*	0.767	0.760	0.762	0.006	0.617	0.437	0.073	0.219
Ganon	0.737	**0.933**	**0.824**	0.923	0.016	0.937	0.937	0.151	0.879
KMCP	**1.000**	0.083	0.154	0.083	0.028	1.438	1.653	0.429	0.826
Kraken2	0.197	**0.933**	0.326	0.920	*0.004*	0.311	0.312	0.070	0.156
Centrifuge	0.154	**0.933**	0.264	**0.930**	**0.003**	**0.189**	*0.244*	*0.053*	*0.122*
Centrifuger	0.283	**0.933**	0.434	*0.924*	**0.003**	0.246	0.261	0.056	0.130
MetaMaps	0.644	**0.933**	0.762	0.914	**0.003**	*0.221*	**0.203**	**0.038**	**0.101**
ONT R10.4									
PanTax	0.761	*0.900*	**0.824**	0.880	0.006	0.532	0.427	0.065	0.214
PanTax (fast)	*0.825*	0.783	*0.803*	0.750	0.006	0.516	0.421	0.073	0.210
Ganon	0.629	**0.933**	0.752	0.917	0.006	0.413	0.402	0.060	0.244
KMCP	**1.000**	0.367	0.537	0.367	0.016	1.173	0.981	0.154	0.490
Kraken2	0.221	**0.933**	0.358	0.919	0.004	0.292	0.293	0.069	0.147
Centrifuge	0.315	**0.933**	0.471	**0.928**	*0.003*	**0.162**	0.221	*0.051*	0.111
Centrifuger	0.368	**0.933**	0.528	*0.923*	*0.003*	0.210	*0.217*	*0.051*	*0.109*
MetaMaps	0.609	**0.933**	0.737	0.913	**0.002**	*0.176*	**0.175**	**0.036**	**0.087**

Supplemental Table S12. Benchmarking results of strain-level taxonomic profiling on the sim-low-sub2 datasets. Note that the best score is marked in bold, and the second best score is marked in italics. AUPR: area under the precision-recall curve. Note that we failed to run Ganon and KMCP on sim-low-sub2 PacBio CLR dataset. KMCP failed because all reference genomes were filtered out, while Ganon failed as no reads matched any reference genome. To account for the presence of low-coverage(0.2×) strains, PanTax adopted a more relaxed threshold on this dataset by setting f_{strain} to 0 and d_{strain} to 1.

Methods	Precision	Recall	F1 score	AUPR	AFE	RFE	L1 distance	L2 distance	BC distance
Illumina									
PanTax (fast)	*0.998*	0.930	*0.963*	0.930	**0.000**	0.172	0.161	0.010	0.081
Ganon	0.792	*0.999*	0.883	0.998	*0.001*	1.156	1.140	0.148	0.572
KMCP	**1.000**	0.976	**0.988**	0.976	**0.000**	*0.148*	*0.136*	*0.008*	*0.068*
Kraken2	0.086	**1.000**	0.159	0.998	*0.001*	0.565	0.588	0.022	0.294
Bracken	0.523	**1.000**	0.687	**1.000**	**0.000**	**0.050**	**0.071**	**0.003**	**0.036**
Centrifuge	0.210	**1.000**	0.347	*0.999*	*0.001*	0.516	0.539	0.021	0.269
Centrifuger	0.619	**1.000**	0.765	**1.000**	**0.000**	0.199	0.199	0.011	0.100
PacBio HiFi									
PanTax (fast)	**1.000**	*0.960*	*0.980*	*0.960*	**0.000**	0.136	0.125	0.007	0.062
Ganon	*0.985*	**1.000**	**0.993**	**1.000**	*0.001*	1.077	1.028	0.128	0.514
KMCP	**1.000**	0.578	0.733	0.578	*0.001*	0.831	0.804	0.033	0.402
Kraken2	0.546	**1.000**	0.706	**1.000**	**0.000**	*0.099*	*0.108*	*0.005*	*0.054*
Centrifuge	0.739	**1.000**	0.850	**1.000**	**0.000**	**0.059**	**0.060**	**0.003**	**0.030**
Centrifuger	0.734	**1.000**	0.846	**1.000**	**0.000**	0.324	0.326	0.013	0.163
MetaMaps	0.187	**1.000**	0.315	**1.000**	**0.000**	0.130	0.170	0.006	0.085
PacBio CLR									
PanTax (fast)	**0.999**	*0.926*	*0.961*	0.925	**0.000**	*0.183*	**0.165**	*0.009*	**0.082**
Ganon	-	-	-	-	-	-	-	-	-
KMCP	-	-	-	-	-	-	-	-	-
Kraken2	0.200	**1.000**	0.334	*0.999*	**0.000**	0.271	0.300	0.011	0.150
Centrifuge	0.057	**1.000**	0.108	**1.000**	**0.000**	**0.153**	0.214	**0.007**	0.107
Centrifuger	0.294	**1.000**	0.454	**1.000**	**0.000**	0.306	0.354	0.013	0.177
MetaMaps	*0.998*	**1.000**	**0.999**	**1.000**	**0.000**	0.190	*0.183*	*0.009*	*0.092*
ONT R9.4.1									
PanTax (fast)	**1.000**	0.961	0.980	0.961	**0.000**	*0.145*	**0.135**	*0.007*	**0.067**
Ganon	*0.980*	*0.995*	*0.988*	*0.994*	*0.001*	0.958	0.958	0.033	0.899
KMCP	**1.000**	0.511	0.676	0.511	*0.001*	0.943	0.917	0.034	0.458
Kraken2	0.193	**1.000**	0.324	**1.000**	**0.000**	0.209	0.230	0.009	0.115
Centrifuge	0.111	**1.000**	0.200	**1.000**	**0.000**	**0.122**	*0.148*	**0.006**	*0.074*
Centrifuger	0.388	**1.000**	0.559	**1.000**	**0.000**	0.215	0.254	0.009	0.127
MetaMaps	*0.980*	**1.000**	**0.990**	**1.000**	**0.000**	0.185	0.180	0.009	0.090
ONT R10.4									
PanTax (fast)	**1.000**	*0.961*	**0.980**	0.961	**0.000**	0.138	0.128	*0.007*	*0.064*
Ganon	*0.925*	**1.000**	*0.961*	*0.999*	*0.001*	0.903	0.881	0.090	0.534
KMCP	**1.000**	0.882	0.937	0.882	**0.000**	0.295	0.263	0.013	0.132
Kraken2	0.261	**1.000**	0.414	**1.000**	**0.000**	0.184	0.199	0.008	0.100
Centrifuge	0.333	**1.000**	0.500	**1.000**	**0.000**	**0.108**	*0.121*	**0.005**	**0.060**
Centrifuger	0.497	**1.000**	0.664	**1.000**	**0.000**	0.171	0.192	*0.007*	0.096
MetaMaps	0.878	**1.000**	0.935	**1.000**	**0.000**	*0.124*	**0.120**	*0.007*	**0.060**

Supplemental Table S13. Benchmarking results of strain-level taxonomic profiling on the sim-high-gtdb datasets. Note that the best score is marked in bold, and the second best score is marked in italics. AUPR: area under the precision-recall curve. Note that we failed to run Ganon and KMCP on sim-high-gtdb PacBio CLR dataset. KMCP failed because all reference genomes were filtered out, while Ganon failed as no reads matched any reference genome.

Methods	Precision	Recall	F1 score	AUPR	AFE	RFE	L1 distance	L2 distance	BC distance
sim-low PacBio HiFi									
PanTax	**1.000**	*0.917*	**0.957**	**0.917**	**0.004**	**0.212**	*0.222*	0.063	*0.111*
PanTax (fast)	**1.000**	*0.917*	**0.957**	**0.917**	**0.004**	*0.213*	**0.218**	*0.062*	**0.109**
hifiasm	*0.771*	0.900	*0.831*	0.829	0.008	0.530	0.529	0.085	0.264
metaMDBG	0.658	0.867	0.748	0.810	*0.006*	0.487	0.454	0.076	0.227
myloasm	0.696	*0.917*	0.791	0.833	*0.006*	0.468	0.451	0.073	0.225
flye	0.700	**0.933**	0.800	*0.889*	**0.004**	0.347	0.274	**0.040**	0.137
sim-low ONT R10.4									
PanTax	*0.982*	**0.933**	*0.957*	*0.927*	**0.003**	*0.188*	*0.192*	*0.045*	*0.096*
PanTax (fast)	**1.000**	**0.933**	**0.966**	**0.933**	**0.003**	**0.187**	**0.178**	**0.044**	**0.089**
metaMDBG	0.659	0.900	0.761	0.865	0.009	0.606	0.564	0.095	0.282
myloasm	0.640	*0.917*	0.753	0.869	*0.006*	0.401	0.401	0.070	0.200
flye	0.675	0.867	0.759	0.784	0.009	0.591	0.571	0.094	0.285
sim-high PacBio HiFi									
PanTax	**0.945**	0.775	**0.852**	*0.714*	**0.000**	*0.404*	*0.451*	*0.021*	*0.226*
PanTax (fast)	*0.940*	0.778	*0.851*	0.712	**0.000**	**0.401**	**0.450**	*0.021*	**0.225**
hifiasm	0.508	*0.793*	0.620	0.690	**0.000**	0.489	0.683	0.027	0.342
metaMDBG	0.422	0.792	0.551	0.671	*0.001*	0.602	0.776	0.029	0.388
myloasm	0.459	0.787	0.580	0.669	*0.001*	0.562	0.746	0.026	0.373
flye	0.458	**0.795**	0.581	**0.720**	**0.000**	0.425	0.580	**0.020**	0.290
sim-high ONT R10.4									
PanTax	*0.932*	0.786	*0.853*	*0.729*	**0.000**	*0.395*	*0.443*	**0.021**	*0.222*
PanTax (fast)	**0.938**	0.786	**0.855**	**0.731**	**0.000**	**0.392**	**0.438**	**0.021**	**0.219**
metaMDBG	0.395	*0.791*	0.527	0.672	*0.001*	0.623	0.802	0.028	0.401
myloasm	0.403	**0.794**	0.534	0.697	*0.001*	0.535	0.704	*0.024*	0.352
flye	0.428	0.786	0.554	0.662	*0.001*	0.665	0.825	0.030	0.413
Zymo1 ONT R10									
PanTax	*0.538*	**0.875**	**0.667**	*0.729*	*0.068*	*0.587*	0.755	0.280	0.377
PanTax (fast)	**0.545**	*0.750*	*0.632*	0.708	*0.068*	0.602	*0.744*	*0.262*	*0.372*
metaMDBG	0.027	**0.875**	0.053	**0.796**	**0.057**	**0.460**	**0.719**	**0.229**	**0.359**
myloasm	0.111	0.250	0.154	0.072	0.108	0.878	1.626	0.561	0.813
flye	0.350	**0.875**	0.500	0.422	0.095	0.744	1.303	0.443	0.652
sim-high-mut2 PacBio HiFi									
PanTax	*0.939*	0.783	**0.854**	*0.723*	**0.000**	*0.394*	**0.441**	*0.021*	**0.221**
PanTax (fast)	**0.945**	0.738	*0.829*	0.675	**0.000**	0.466	*0.512*	0.023	*0.256*
hifiasm	0.487	**0.795**	0.604	0.715	**0.000**	*0.394*	0.579	0.022	0.290
metaMDBG	0.409	**0.795**	0.540	**0.725**	**0.000**	**0.383**	0.554	**0.020**	0.277
myloasm	0.440	*0.794*	0.567	0.698	**0.000**	0.440	0.621	0.022	0.310
flye	0.463	**0.795**	0.585	0.715	**0.000**	0.419	0.580	**0.020**	0.290
sim-high-mut2 ONT R10.4									
PanTax	*0.929*	0.785	**0.851**	**0.730**	**0.000**	**0.391**	**0.443**	**0.021**	**0.221**
PanTax (fast)	**0.935**	0.739	*0.826*	0.678	**0.000**	0.459	*0.514*	*0.023*	*0.257*
metaMDBG	0.371	**0.795**	0.506	*0.728*	**0.000**	0.473	0.641	**0.021**	0.320
myloasm	0.377	**0.795**	0.512	0.720	**0.000**	*0.454*	0.618	**0.021**	0.309
flye	0.431	*0.794*	0.558	0.697	*0.001*	0.541	0.699	0.025	0.349

Supplemental Table S14. Benchmarking results of strain-level taxonomic profiling compared to assembly tools. Note that the best score is marked in bold, and the second best score is marked in italics. AUPR: area under the precision-recall curve.

Methods	Precision	Recall	F1 score	AUPR	AFE	RFE	L1 distance	L2 distance	BC distance
SimRef Illumina									
SimRef1	**1.000**	**1.000**	**1.000**	**1.000**	**0.001**	**0.060**	**0.036**	**0.006**	**0.018**
SimRef2	**1.000**	**1.000**	**1.000**	**1.000**	**0.001**	*0.063*	*0.039*	*0.007*	*0.020*
SimRef3	**1.000**	**1.000**	**1.000**	**1.000**	**0.001**	0.064	0.040	*0.007*	*0.020*
SimRef4	**1.000**	**1.000**	**1.000**	**1.000**	**0.001**	0.064	0.043	0.008	0.022
SimRef5	*0.984*	**1.000**	*0.992*	**1.000**	*0.002*	0.101	0.110	0.035	0.055
SimRef PacBio HiFi									
SimRef1	**1.000**	**1.000**	**1.000**	**1.000**	**0.001**	**0.084**	**0.058**	**0.010**	**0.029**
SimRef2	**1.000**	**1.000**	**1.000**	**1.000**	**0.001**	*0.087*	*0.060*	**0.010**	*0.030*
SimRef3	**1.000**	**1.000**	**1.000**	**1.000**	**0.001**	0.090	*0.060*	**0.010**	*0.030*
SimRef4	**1.000**	**1.000**	**1.000**	**1.000**	**0.001**	0.094	0.065	*0.011*	0.033
SimRef5	**1.000**	*0.983*	*0.992*	*0.983*	**0.001**	0.119	0.070	0.012	0.035
SimRef PacBio CLR									
SimRef1	**1.000**	**1.000**	**1.000**	**1.000**	**0.001**	**0.112**	**0.079**	**0.013**	**0.039**
SimRef2	**1.000**	**1.000**	**1.000**	**1.000**	**0.001**	*0.123*	**0.079**	**0.013**	**0.039**
SimRef3	**1.000**	**1.000**	**1.000**	**1.000**	**0.001**	0.124	*0.080*	**0.013**	*0.040*
SimRef4	*0.984*	**1.000**	*0.992*	**1.000**	**0.001**	*0.123*	0.085	*0.015*	0.042
SimRef5	**1.000**	*0.967*	0.983	*0.967*	*0.002*	0.155	0.096	0.018	0.048
SimRef ONT R9.4.1									
SimRef1	**1.000**	**1.000**	**1.000**	**1.000**	**0.001**	**0.093**	**0.061**	**0.010**	**0.031**
SimRef2	**1.000**	**1.000**	**1.000**	**1.000**	**0.001**	*0.108*	*0.069*	*0.011*	*0.034*
SimRef3	**1.000**	**1.000**	**1.000**	**1.000**	**0.001**	0.117	0.074	0.012	0.037
SimRef4	**1.000**	**1.000**	**1.000**	**1.000**	**0.001**	0.111	*0.069*	*0.011*	0.035
SimRef5	**1.000**	*0.983*	*0.992*	*0.983*	**0.001**	0.121	0.076	0.014	0.038
SimRef ONT R10.4									
SimRef1	**1.000**	**1.000**	**1.000**	**1.000**	**0.001**	0.090	0.062	*0.010*	0.031
SimRef2	**1.000**	**1.000**	**1.000**	**1.000**	**0.001**	**0.075**	**0.053**	**0.009**	**0.026**
SimRef3	**1.000**	**1.000**	**1.000**	**1.000**	**0.001**	*0.079*	*0.059*	0.011	*0.030*
SimRef4	**1.000**	**1.000**	**1.000**	**1.000**	**0.001**	0.081	0.060	0.011	*0.030*
SimRef5	**1.000**	*0.983*	*0.992*	*0.983*	**0.001**	0.117	0.075	0.015	0.037
Zymo1 ONT R10									
ZymoRef1	*0.467*	**0.875**	*0.609*	*0.745*	*0.069*	*0.593*	0.766	**0.277**	0.383
ZymoRef2	**0.538**	**0.875**	**0.667**	**0.765**	0.070	0.607	*0.761*	*0.280*	*0.380*
ZymoRef3	**0.538**	**0.875**	**0.667**	0.729	**0.068**	**0.587**	**0.755**	*0.280*	**0.377**

Supplemental Table S15. Benchmarking results of strain-level taxonomic profiling by PanTax across reference diversity. Note that the best score is marked in bold, and the second best score is marked in italics. AUPR: area under the precision-recall curve.

Method	PanTax	PanTax(fast)	Ganon	KMCP	Kraken2	Bracken	Centrifuge	Centrifuger
**Database build time**								
CPU(h)	791.0+17.7	-	6.5	4.9	567.0	7.8	50.3	151.9
Wall Time(h)	27.4+5.9	-	0.8	1.4	19.6	0.6	6.8	6.1
Memory(G)	516.8	-	67.8	7.1	51.2	63.6	357.9	77.8
**sim-low Illumina**								
CPU(h)	815.1	9.0+0.1+1.1	7.5	19.3	567.1	7.8	51.2	152.6
Wall Time(h)	34.2	0.3+0.0+0.0	0.9	1.8	19.6	0.6	6.8	6.2
Memory(G)	489.8	17.9	36.2	18.8	53.3	0.0	23.8	20.2
**sim-high Illumina**								
CPU(h)	865.9	192.2+2.7+18.7	12.2	115.3	567.6	7.8	59.2	158.7
Wall Time(h)	36.1	4.9+1.2+0.6	1.2	4.2	19.7	0.6	7.0	6.4
Memory(G)	491.7	132.4	41.7	18.9	60.8	0.0	24.7	20.2
**PD human gut Illumina**								
CPU(h)	809.7	15.8+0.1+0.7	7.2	31.9	567.2	7.8	52.1	153.2
Wall Time(h)	33.9	0.6+0.0+0.0	0.9	2.2	19.7	0.6	6.9	6.2
Memory(G)	490.0	15.2	37.7	18.9	53.6	0.0	24.2	20.4

Supplemental Table S16. Runtime and memory usage of benchmarking tools on sim-low, sim-high and PD human gut Illumina datasets. We measured the CPU time, wall time, and maximum RAM usage required by the benchmark tool to build the database (RefSeq: 13404) and perform taxonomic profiling using 64 threads. In the table, some time values are separated by "+". For PanTax's Database build time, it represents the pangenome construction time and index construction time, respectively. For PanTax (fast)'s taxonomic profiling time, the time values correspond to the pangenome construction time, index construction time, and the time required for performing taxonomic profiling on the respective dataset. Notably, for all tools, the taxonomic profiling time includes their Database build time.

Method	PanTax	PanTax(fast)	Ganon	KMCP	Kraken2	Centrifuge	Centrifuger	MetaMaps
**Database build time**								
CPU(h)	791.0	-	6.5	4.9	567.0	50.3	151.9	0.2
Wall Time(h)	27.4	-	0.8	1.4	19.6	6.8	6.1	0.2
Memory(G)	40.0	-	67.8	7.1	51.2	357.9	77.8	5.4
**sim-low PacBio HiFi**								
CPU(h)	855.1	8.2+5.7	6.6	19.3	567.1	51.3	152.7	10.4
Wall Time(h)	31.1	0.3+0.2	0.9	1.8	19.6	6.8	6.2	3.4
Memory(G)	475.5	7.3	37.1	19.5	52.5	23.8	22.5	417.6
**sim-low PacBio CLR**								
CPU(h)	866.9	5.9+4.3	-	-	567.1	54.4	152.5	7.1
Wall Time(h)	31.3	0.3+0.1	-	-	19.6	6.9	6.1	4.4
Memory(G)	475.2	5.0	-	-	52.9	24.3	22.5	416.9
**sim-low ONT R9.4.1**								
CPU(h)	861.4	7.0+5.1	6.5	19.7	567.1	54.6	152.7	9.6
Wall Time(h)	31.1	0.2+0.2	0.9	2.0	19.6	6.9	6.2	4.5
Memory(G)	475.0	6.7	37.1	20.8	52.9	24.4	22.5	418.6
**sim-low ONT R10.4**								
CPU(h)	859.7	8.1+5.8	6.6	25.3	567.1	53.2	152.9	12.3
Wall Time(h)	31.0	0.3+0.2	0.9	2.7	19.6	6.8	6.2	4.4
Memory(G)	474.8	7.5	37.1	20.2	52.8	24.1	22.5	418.6
**sim-high PacBio HiFi**								
CPU(h)	1057.0	186.4+149.6	10.0	131.2	567.5	55.6	158.6	147.2
Wall Time(h)	35.4	4.4+2.5	0.9	4.1	19.7	6.9	6.5	5.5
Memory(G)	475.6	68.8	37.1	19.6	53.6	24.2	28.0	418.1
**sim-high PacBio CLR**								
CPU(h)	1148.1	137.1+134.8	-	-	567.4	62.9	156.6	47.7
Wall Time(h)	35.1	3.3+2.3	-	-	19.7	7.2	6.5	4.1
Memory(G)	475.0	54.2	-	-	56.4	29.1	33.1	417.0
**sim-high ONT R9.4.1**								
CPU(h)	1120.1	180.1+149.7	6.9	156.2	567.5	53.4	158.5	103.0
Wall Time(h)	36.7	4.3+2.5	0.9	4.7	19.6	6.9	6.7	5.5
Memory(G)	475.7	67.3	37.0	20.9	54.0	29.3	35.7	418.7
**sim-high ONT R10.4**								
CPU(h)	1111.6	182.5+162.4	7.4	248.5	567.5	58.5	159.8	147.4
Wall Time(h)	34.0	4.4+2.7	0.9	10.3	19.7	6.9	7.0	6.2
Memory(G)	474.2	67.9	37.1	20.3	54.6	26.8	31.8	418.8
**Healthy human gut ONT**								
CPU(h)	1041.4	48.0+22.1	-	-	567.3	53.0	154.1	16.0
Wall Time(h)	32.6	1.1+0.4	-	-	19.6	6.9	6.2	6.1
Memory(G)	476.9	28.6	-	-	52.9	25.4	26.3	416.0
**Omnivorous human gut PacBio HiFi**								
CPU(h)	1012.4	56.8+23.0	8.2	48.8	567.3	53.0	155.5	37.0
Wall Time(h)	32.1	1.3+0.4	0.8	2.2	19.7	6.9	6.2	5.8
Memory(G)	475.5	20.7	41.6	19.9	52.8	24.0	27.7	417.2

Supplemental Table S17. Runtime and memory usage of benchmarking tools on sim-low, sim-high, Healthy human gut and Omnivorous human gut long-read datasets. We measured the CPU time, wall time, and maximum RAM usage required by the benchmark tool to build the database (RefSeq: 13404) and perform taxonomic profiling using 64 threads. We failed to run Ganon and KMCP on sim-high-gtdb PacBio CLR dataset and Healthy human gut ONT dataset. In the table, some time values are separated by "+". For PanTax (fast)'s taxonomic profiling time, the time values correspond to the pangenome construction time and the time required for performing taxonomic profiling on the respective dataset. Notably, for all tools, the taxonomic profiling time includes their Database build time.

Method	PanTax(fast)	Ganon	KMCP	Kraken2	Bracken	Centrifuge	Centrifuger	MetaMaps
**Database build time**								
CPU(h)	-	40.4	34.5	2672.3	99.2	3797.2	4683.2	3.3
Wall Time(h)	-	20.4	12.7	58.7	2.4	171.0	96.5	91.3
Memory(G)	-	474.5	22.7	557.7	570.3	1175.3	737.4	21.6
**sim-high-gtdb Illumina**								
CPU(h)	74.2+0.6+9.3	55.7	506.1	2672.8	99.2	3806.3	4688.5	-
Wall Time(h)	6.4+0.1+0.8	21.2	22.5	59.4	2.4	171.7	97.1	-
Memory(G)	34.6	415.5	221.1	564.9	0.1	804.5	303.6	-
**sim-high-gtdb PacBio HiFi**								
CPU(h)	75.1+61.0	42.9	525.5	2672.8	-	3801.9	4689.2	138.5
Wall Time(h)	7.5+1.6	20.8	20.7	59.3	-	171.7	96.9	136.0
Memory(G)	29.1	418.6	222.1	557.8	-	804.1	309.7	694.9
**sim-high-gtdb PacBio CLR**								
CPU(h)	50.8+59.8	-	-	2672.9	-	3801.3	4688.5	96.0
Wall Time(h)	2.5+1.7	-	-	59.0	-	171.3	97.0	133.1
Memory(G)	29.1	-	-	562.8	-	805.7	309.5	694.4
**sim-high-gtdb ONT R9.4.1**								
CPU(h)	79.6+66.8	42.2	415.3	2672.9	-	3801.9	4689.2	145.0
Wall Time(h)	4.3+1.5	20.8	22.7	59.3	-	171.7	96.9	144.1
Memory(G)	29.1	416.9	222.5	560.7	-	805.3	306.5	695.3
**sim-high-gtdb ONT R10.4**								
CPU(h)	54.7+63.0	42.2	472.1	2672.9	-	3804.7	4689.5	173.0
Wall Time(h)	5.0+1.9	21.0	20.1	59.3	-	172.6	99.3	139.7
Memory(G)	29.1	416.8	222.0	559.7	-	804.8	312.1	695.3

Supplemental Table S18. Runtime and memory usage of benchmarking tools on sim-high-gtdb datasets. We measured the CPU time, wall time, and maximum RAM usage required by the benchmark tool to build the database (GTDB: 206273) and perform taxonomic profiling using 64 threads. We failed to run Ganon and KMCP on sim-high-gtdb PacBio CLR dataset. In the table, some time values are separated by "+". For PanTax (fast)'s taxonomic profiling time on the Illumina dataset, it represents the pangenome construction time, index construction time, and the time required to perform taxonomic profiling on the respective dataset. For PanTax (fast)'s taxonomic profiling time on long-read datasets, the time values correspond to the pangenome construction time and the time required for performing taxonomic profiling on the respective dataset. Notably, for all tools, the taxonomic profiling time includes their Database build time.

Method	PanTax	StrainScan	StrainGE	StrainEst
**Database build time**				
CPU(h)	26.4+0.1	2.5	0.3	4.4
Wall Time(h)	0.7+0.1	0.8	0.4	1.3
Memory(G)	21.5	19.2	0.7	3.7
**3 strains**				
CPU(h)	26.6	2.5	0.3	6.4
Wall Time(h)	0.8	0.8	0.4	6.9
Memory(G)	2.9	1.3	0.5	0.5
**5 strains**				
CPU(h)	26.6	2.5	0.3	6.6
Wall Time(h)	0.8	0.8	0.4	7.5
Memory(G)	3.2	1.3	0.8	0.5
**10 strains**				
CPU(h)	26.8	2.5	0.3	7.7
Wall Time(h)	0.9	0.8	0.4	10.6
Memory(G)	3.4	1.3	1.4	0.5

Supplemental Table S19. Runtime and memory usage of benchmarking tools on *S. epidermidis* strain mixtures Illumina datasets. We measured the CPU time, wall time, and maximum RAM usage required by the benchmark tool to build the database (Complete genomes of *S. epidermidis* on RefSeq) and perform taxonomic profiling using 64 threads. In the table, some time values are separated by "+". For PanTax's Database build time, it represents the pangenome construction time and index construction time, respectively. Notably, for all tools, the taxonomic profiling time includes their Database build time.

Solvers	Precision	Recall	F1 score	AUPR	BC distance	CPU time(min)	Wall time(min)
sim-low Illumina							
Gurobi	0.933	0.933	0.933	0.930	0.100	0.7	8.0
Cbc	0.933	0.933	0.933	0.930	0.100	1146.5	8563.6
GLPK	0.933	0.933	0.933	0.930	0.100	2363.5	18015.9
HiGHS	0.933	0.933	0.933	0.931	0.100	662.7	6036.4

Supplemental Table S20. Benchmarking results of strain-level taxonomic profiling with different solvers. Except for GLPK, which is restricted to single-thread execution, all other solvers were configured to run with 64 threads.
