# Supplementary material for: Strain-level metagenomic profiling using pangenome graphs with PanTax
Source: Genome Res. 2026 Feb;36(2):405–20. doi: 10.1101/gr.280858.125 (PMC12863173; doi:10.1101/gr.280858.125)
Supplement: Supplement 4 [file Supplemental_Figures.pdf]

Strain-level metagenomic profiling using pangenome graphs with PanTax

Wenhai Zhang[1,2,†], Yuansheng Liu[3,†], Guangyi Li[2,†], Jialu Xu[2], Enlian Chen[2], Alexander Schönhuth[4,*] Xiao Luo[1,2,*]

[1] Hunan Research Center of the Basic Discipline for Cell Signaling, Hunan University, Changsha, Hunan 410082, China
[2] College of Biology, Hunan University, Changsha, Hunan 410082, China
[3] College of Computer Science and Electronic Engineering, Hunan University, Changsha, Hunan 410082, China
[4] Faculty of Technology, Bielefeld University, Bielefeld 33615, Germany
[†]These authors contributed equally to the work.
[*]To whom correspondence should be addressed.
Email: aschoen@cebitec.uni-bielefeld.de
Email: xluo@hnu.edu.cn

Supplemental Figures

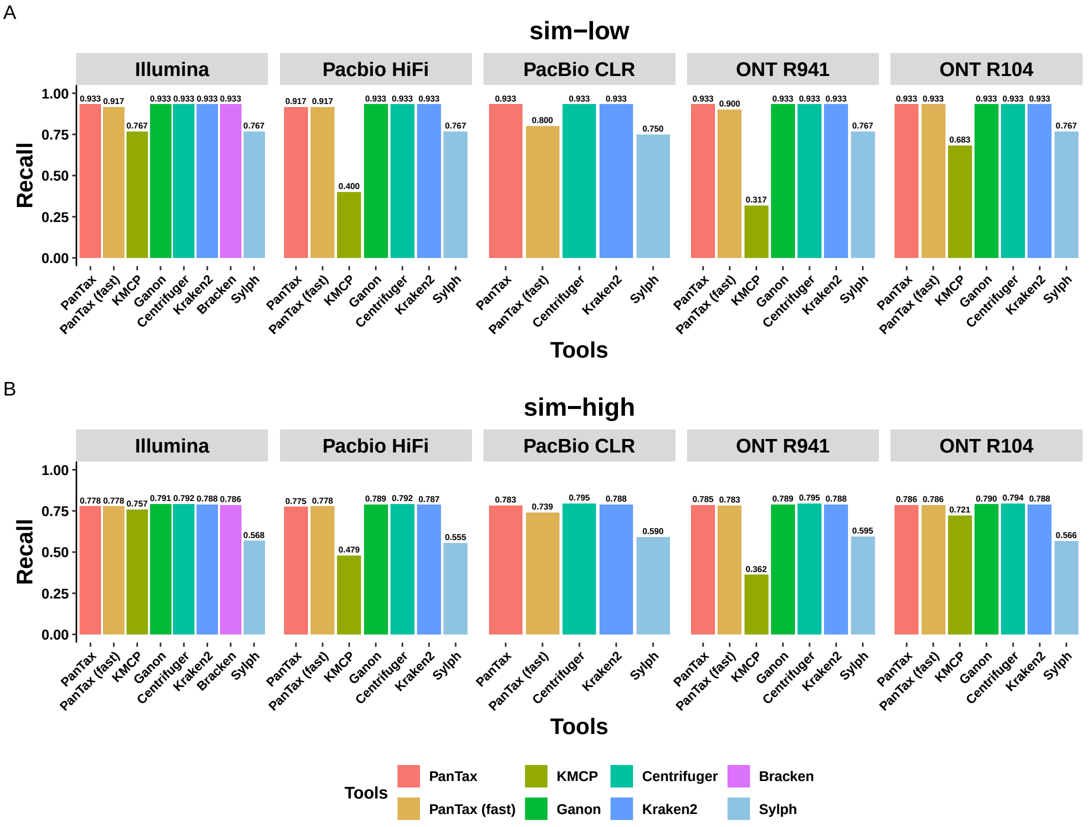

Supplemental Figure S1. Strain-level recall of profiling tools on simulated datasets, compared with the species-level profiling tool sylph. (A) sim–low and (B) sim–high datasets across five sequencing platforms.

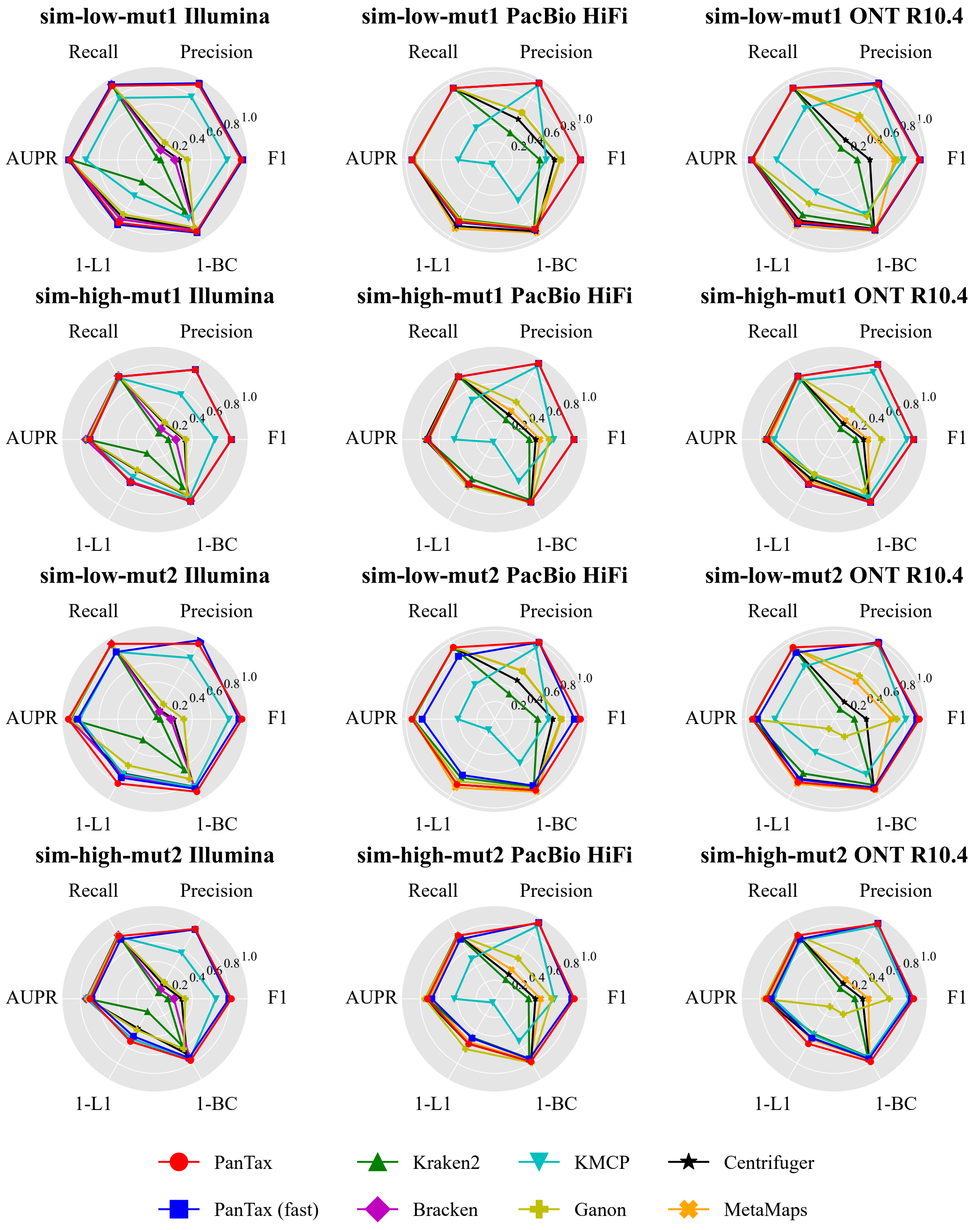

Supplemental Figure S2. Benchmarking results of strain-level taxonomic profiling on the simulated datasets with introduced mutations (sim-low-mut1, sim-high-mut1, sim-low-mut2, sim-high-mut2). AUPR: area under the precision-recall curve. To visualize all metrics consistently (i.e., with higher values indicating better performance), we present the 1-L1 distance and 1-BC distance.

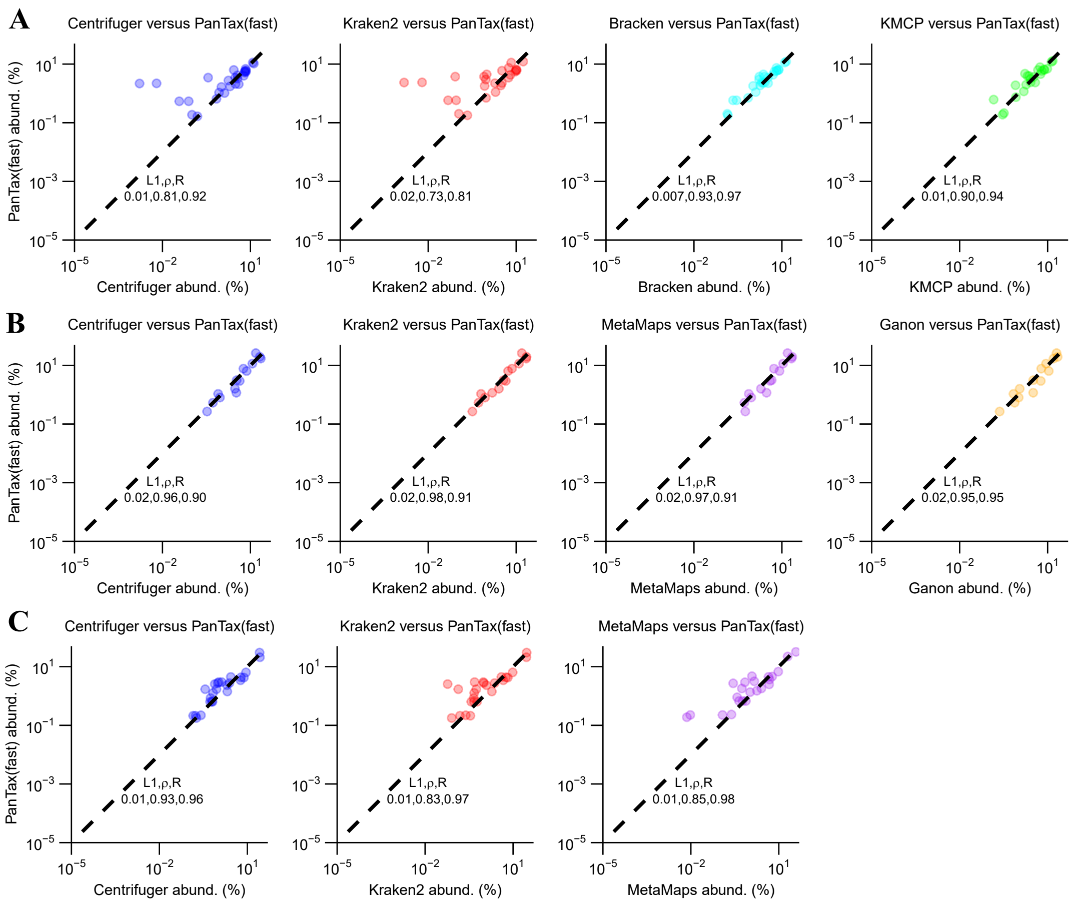

Supplemental Figure S3. Results of strain-level taxonomic profiling for real human gut metagenomes. **(A)** PD human gut (Illumina) dataset, **(B)** Omnivorous human gut (PacBio HiFi) dataset, and **(C)** Healthy human gut (ONT) dataset. For all three datasets, the relative taxon abundance correlation between PanTax (fast) and other competitive profilers was computed. The comparisons included mean L1 distance, Spearman correlation, and Pearson correlation. Note that we failed to run Ganon and KMCP on the Healthy human gut (ONT) dataset because it was primarily designed for short reads.

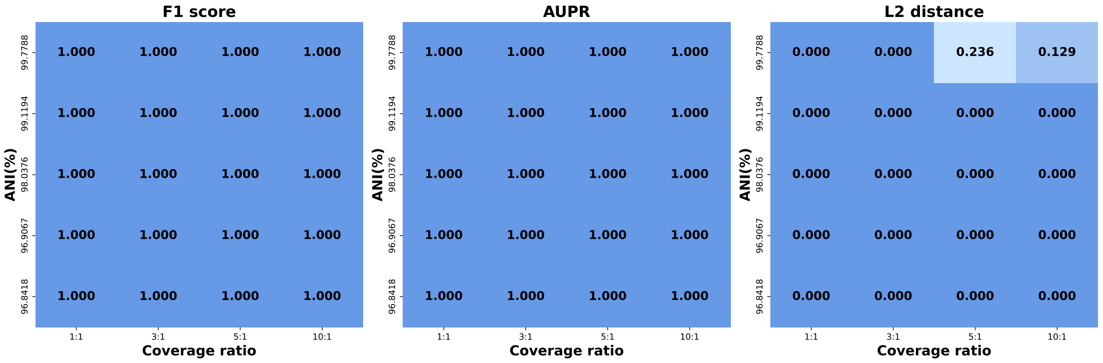

Supplemental Figure S4. Evaluation of PanTax's performance in mixtures of two strains (of the same species) with varying strain coverage and ANI using simulated Illumina reads. The x-axis represents the coverage ratio, while the y-axis depicts the ANI (Average Nucleotide Identity) of the two strains in the mixture. The three panels correspond to the F1 score, AUPR (Area Under the Precision-Recall Curve), and L2 distance of the strain-level taxonomic profiling results, respectively. Darker shades indicate superior taxonomic performance.

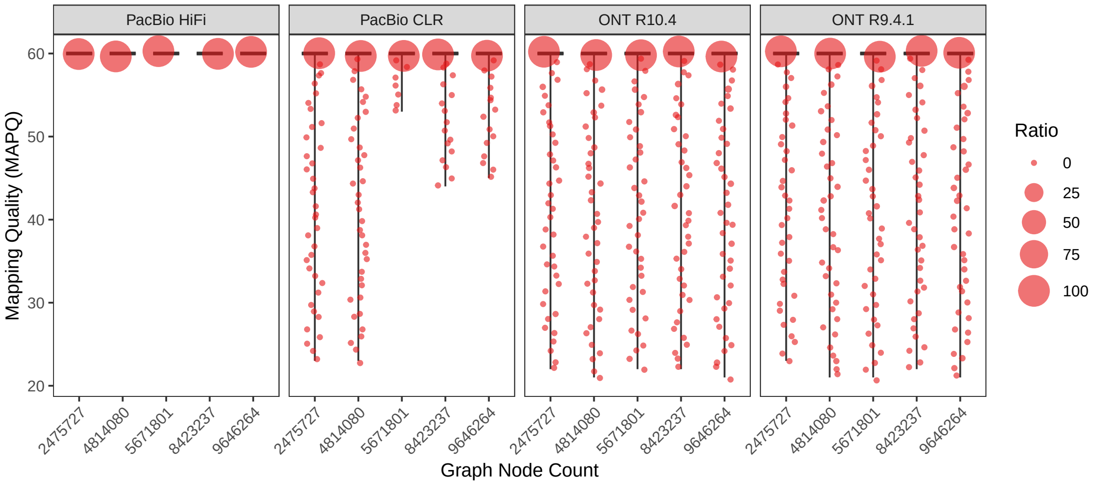

Supplemental Figure S5. Read mapping quality across graph complexity and long read sequencing technologies. Each point represents the proportion of reads at a given mapping quality. The x-axis shows the number of nodes in the reference pangenome graph, and the y-axis indicates the mapping quality of each node.

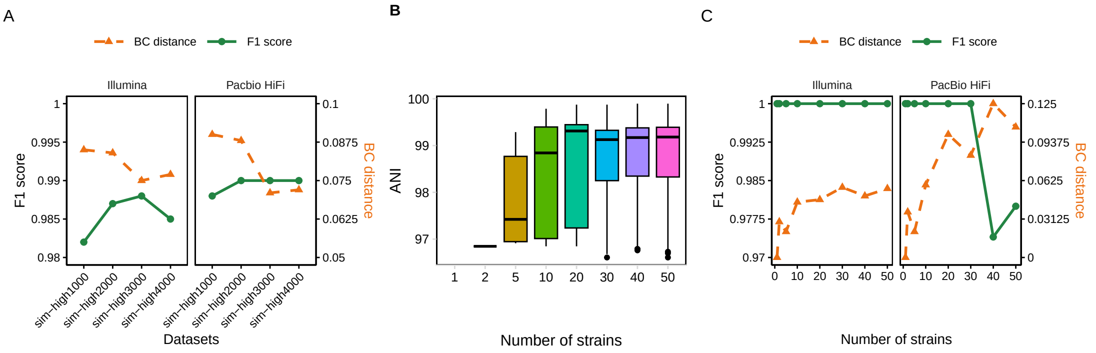

Supplemental Figure S6. Results of PanTax for scaling limit benchmarks. **(A)** F1 score and BC distance of PanTax on Illumina and PacBio HiFi datasets under strain quantity scaling (sim-high1000, sim-high2000, sim-high3000, and sim-high4000). **(B)** Boxplots showing the distribution of average nucleotide identity (ANI) for genome similarity scaling datasets with 1 to 50 strains. **(C)** F1 score and BC distance of PanTax on Illumina and PacBio HiFi datasets under genome similarity scaling with 1 to 50 strains.

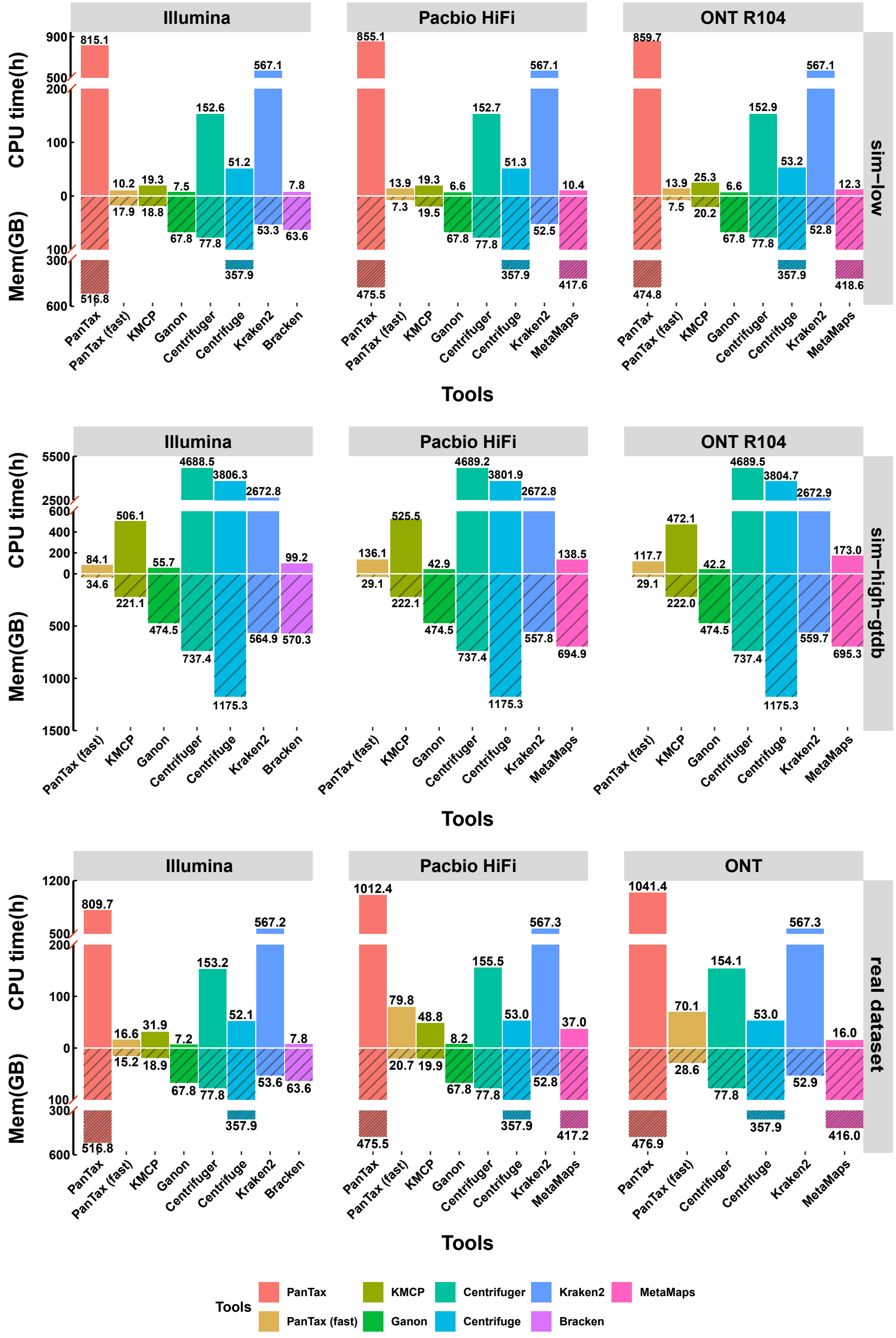

Figure S7. The CPU time and peak memory usage (Mem) of various metagenomic profilers. The upper, middle, and bottom panels represent the sim-low, sim-high-gtdb, and real human gut datasets, respectively. Note that for the real datasets, from left to right, the panels correspond to the PD human gut (Illumina), Omnivorous human gut (PacBio HiFi), and Healthy human gut (ONT) datasets.

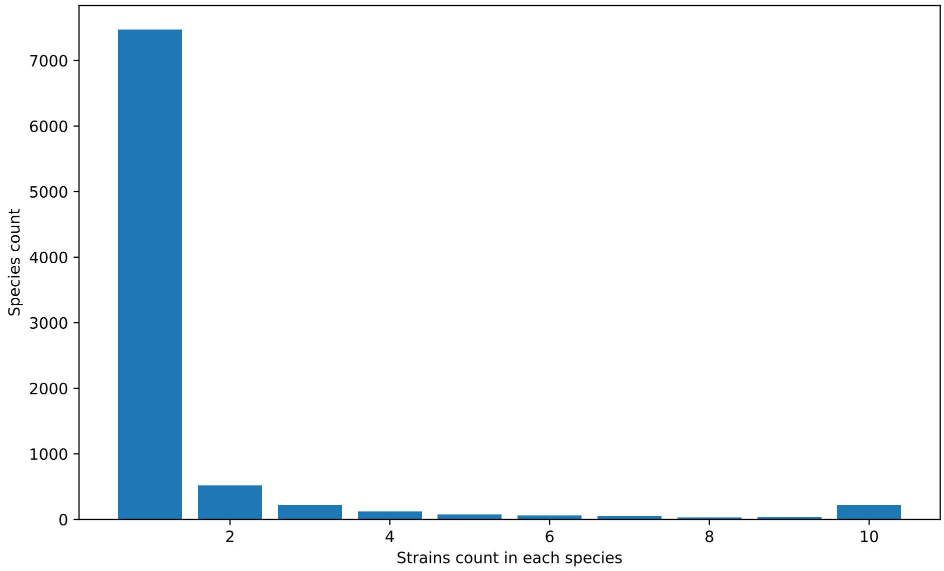

Supplemental Figure S8. Distribution of strain numbers across species. The bar chart depicts the number of strains (genomes) represented for each species in the pangenome reference dataset used in this study. The X-axis indicates the number of strains within each species, while the Y-axis represents the number of species.

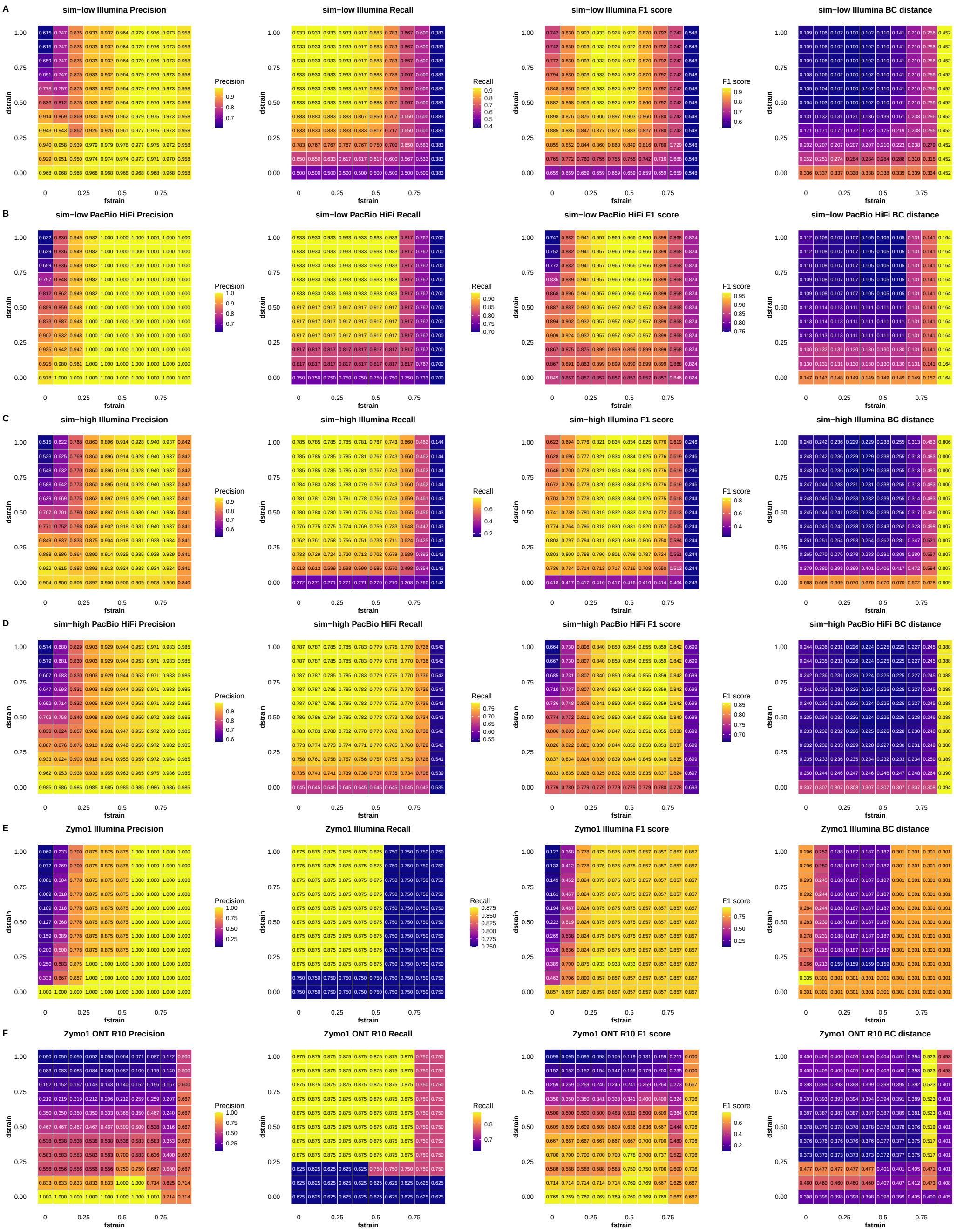

Supplemental Figure S9. Sensitivity analysis of two key parameters (f_{strain} and d_{strain}) in strain level profiling. Performance of PanTax was evaluated on **(A)** sim–low Illumina, **(B)** sim–low PacBio HiFi, **(C)** sim–high Illumina, **(D)** sim–high PacBio HiFi, **(E)** Zymo1 Illumina, and **(F)** Zymo1 ONT R10.4 datasets. Heatmaps show Precision, Recall, F1 score, and BC distance across varying values of f_{strain} and d_{strain}.

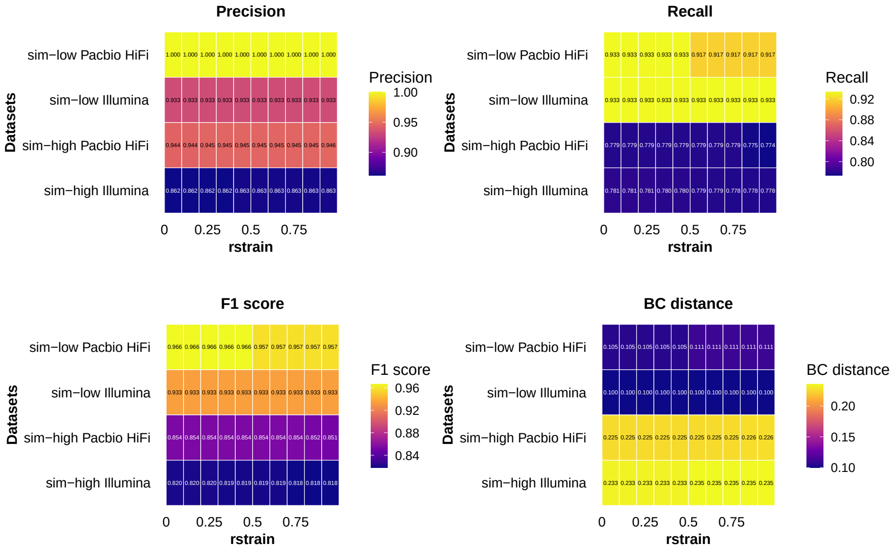

Supplemental Figure S10. Sensitivity analysis of key parameter (r_{strain}) in strain level profiling. Performance of PanTax was evaluated on sim–low PacBio HiFi, sim–low Illumina, sim–high PacBio HiFi, and sim–high Illumina datasets. Heatmaps display Precision, Recall, F1 score, and BC distance across varying r_{strain}, with f_{strain} and d_{strain} held constant.

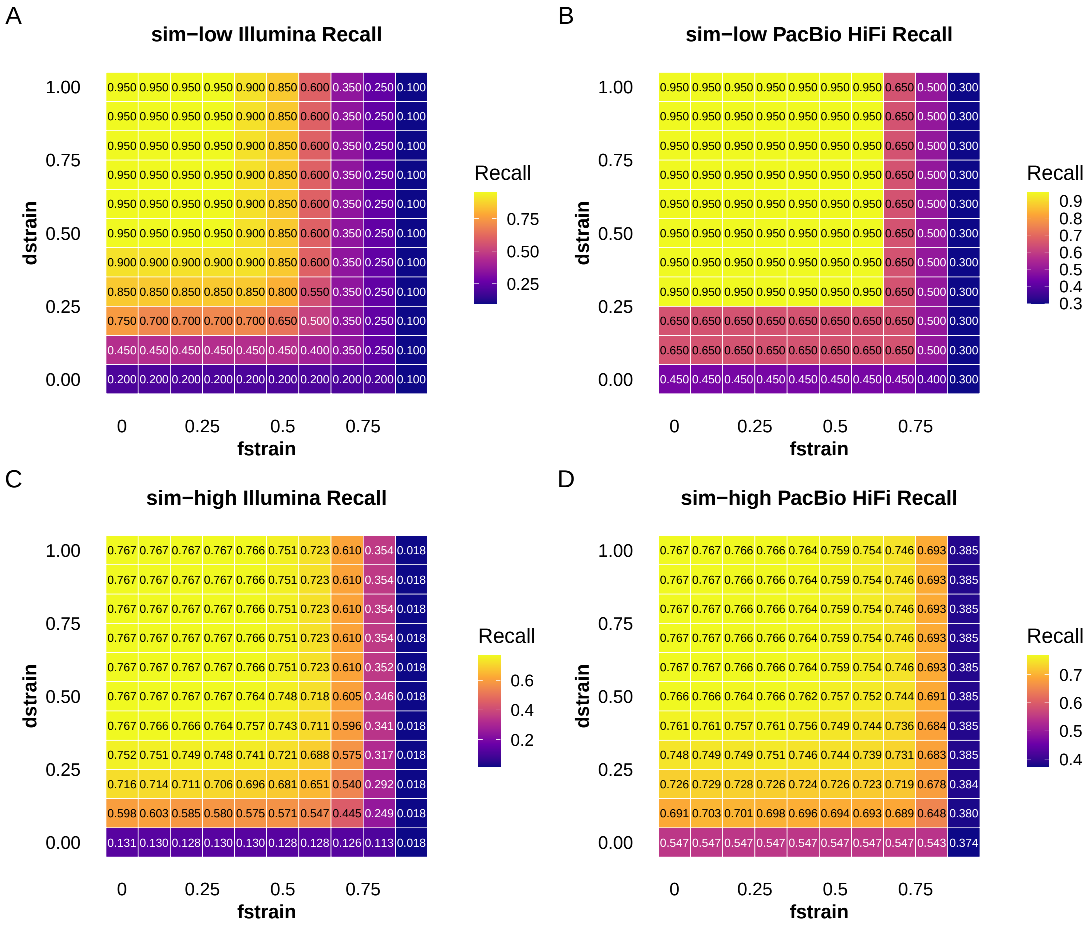

Supplemental Figure S11. Sensitivity analysis of key parameters (f_{strain} and d_{strain}) in strain-level profiling for low coverage ($\leq 3\times$) strains. Performance of PanTax was evaluated on **(A)** sim–low Illumina, **(B)** sim–low PacBio HiFi, **(C)** sim–high Illumina, and **(D)** sim–high PacBio HiFi datasets. Heatmaps show the recall of low coverage strains across varying values of f_{strain} and d_{strain}.
